# Angpt2/Tie2 autostimulatory loop controls tumorigenesis

**DOI:** 10.15252/emmm.202114364

**Published:** 2022-03-10

**Authors:** Ninelia Minaskan Karabid, Tobias Wiedemann, Sebastian Gulde, Hermine Mohr, Renu Chandra Segaran, Julia Geppert, Maria Rohm, Giovanni Vitale, Germano Gaudenzi, Alessandra Dicitore, Donna Pauler Ankerst, Yiyao Chen, Rickmer Braren, Georg Kaissis, Franz Schilling, Mathias Schillmaier, Graeme Eisenhofer, Stephan Herzig, Federico Roncaroli, Jürgen B Honegger, Natalia S Pellegata

**Affiliations:** ^1^ Institute for Diabetes and Cancer Helmholtz Zentrum München Neuherberg Germany; ^2^ Joint Heidelberg‐IDC Translational Diabetes Program Heidelberg University Hospital Heidelberg Germany; ^3^ Istituto Auxologico Italiano IRCCS Laboratory of Geriatric and Oncologic Neuroendocrinology Research Cusano Milanino Milan Italy; ^4^ Department of Medical Biotechnology and Translational Medicine University of Milan Milan Italy; ^5^ Department of Mathematics Technical University Munich Garching Germany; ^6^ Institute for Diagnostic and Interventional Radiology Klinikum Rechts der Isar Technical University Munich Munich Germany; ^7^ Department of Nuclear Medicine Klinikum rechts der Isar Technical University Munich Munich Germany; ^8^ Institute of Clinical Chemistry and Laboratory Medicine University Hospital Carl Gustav Carus Technische Universität Dresden Dresden Germany; ^9^ Division of Neuroscience and Experimental Psychology Faculty of Biology, Medicine and Health University of Manchester Manchester UK; ^10^ Department of Neurosurgery Eberhard Karls University Tübingen Tübingen Germany

**Keywords:** angiopoietin 2, anti‐angiopoietin biologicals, PitNETs, tumor/endothelial cell crosstalk, tumor‐bound Tie2, Cancer, Vascular Biology & Angiogenesis

## Abstract

Invasive nonfunctioning (NF) pituitary neuroendocrine tumors (PitNETs) are non‐resectable neoplasms associated with frequent relapses and significant comorbidities. As the current therapies of NF‐PitNETs often fail, new therapeutic targets are needed. The observation that circulating angiopoietin‐2 (ANGPT2) is elevated in patients with NF‐PitNET and correlates with tumor aggressiveness prompted us to investigate the ANGPT2/TIE2 axis in NF‐PitNETs in the GH3 PitNET cell line, primary human NF‐PitNET cells, xenografts in zebrafish and mice, and in MENX rats, the only autochthonous NF‐PitNET model. We show that PitNET cells express a functional TIE2 receptor and secrete bioactive ANGPT2, which promotes, besides angiogenesis, tumor cell growth in an autocrine and paracrine fashion. ANGPT2 stimulation of TIE2 in tumor cells activates downstream cell proliferation signals, as previously demonstrated in endothelial cells (ECs). *Tie2* gene deletion blunts PitNETs growth in xenograft models, and pharmacological inhibition of Angpt2/Tie2 signaling antagonizes PitNETs in primary cell cultures, tumor xenografts in mice, and in MENX rats. Thus, the ANGPT2/TIE2 axis provides an exploitable therapeutic target in NF‐PitNETs and possibly in other tumors expressing ANGPT2/TIE2. The ability of tumor cells to coopt angiogenic signals classically viewed as EC‐specific expands our view on the microenvironmental cues that are essential for tumor progression.

## Graphical abstract

There is currently no treatment for nonfunctioning pituitary neuroendocrine tumors (NF‐PitNETs), which are intracranial tumors associated with frequent relapse and severe comorbidities. This study establishes the ANGPT2/Tie2 signaling axis as a novel therapeutic target for NF‐PitNETs.

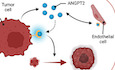

**The paper explained**

**Problem**

Nonfunctioning pituitary neuroendocrine tumors (NF‐PitNETs) are the second most common type of PitNETs and affect patients' functional status and mortality. Given the lack of symptoms linked to hormone hypersecretion, NF‐PitNETs are often diagnosed upon tumor mass detection. At this stage, 50% of the tumors have invaded surrounding structures and cannot be completely removed by surgery. Relapse is thus frequent, causing significant comorbidities. These tumors do not respond to standard‐of‐care treatments, which makes the identification of novel therapeutic targets mandatory to improve patients' management.

**Results**

Circulating levels of the angiopoietin 2 (ANGPT2) cytokine are elevated in NF‐PitNET patients and correlate with tumor aggressiveness. NF‐PitNET cells express and secrete ANGPT2, which stimulates the proliferation/survival of tumor cells *in vitro*, and angiogenesis in PitNET cell xenografts in zebrafish embryos *in vivo*. Noteworthy, NF‐PitNET cells possess a functional TIE2 receptor, which is activated by ANGPT2 and further stimulates downstream mitogenic signals. This establishes an autocrine/paracrine stimulatory loop in NF‐PitNET cells, as demonstrated in endothelial cells. Deletion of TIE2 in PitNET cells suppresses their growth in mouse xenografts *in vivo*. Proof‐of‐principle pharmacological inhibition of ANGPT2/TIE2 signaling antagonizes NF‐PitNETs in primary tumor cultures, as well as in mouse xenografts and in MENX rats, the only model of spontaneous NF‐PitNETs.

**Impact**

Our study identifies an active ANGPT2/TIE2 signaling cascade in NF‐PitNET cells. The role of this axis in sustaining tumor cell growth and mediating a crosstalk between tumor and endothelial cells in the tumor microenvironment makes it an attractive therapeutic target for the treatment of NF‐PitNETs.

## Introduction

Pituitary adenomas are the third most common intracranial neoplasm and considerably impact morbidity and mortality of affected patients (Ntali *et al*, 2016; Lobatto *et al*, 2018). Now renamed pituitary neuroendocrine tumors (PitNETs), these tumors, though usually benign, often invade surrounding structures and cannot be cured using standard therapies (Asa *et al*, 2017). Invasive pituitary tumors, including nonfunctioning (NF) adenomas derived from gonadotroph cells (NFPAs or NF‐PitNETs), cannot be completely resected thereby leading to frequent relapse and significant comorbidities (Tampourlou *et al*, 2018). Dopamine agonists, somatostatin analogues, and temozolomide have all yielded disappointing therapeutic results, and radiotherapy is associated with significant side effects (Greenman & Melmed, 1996). Thus, novel treatment strategies are needed to treat invasive/recurrent NF‐PitNETs.

Tumorigenesis is a highly dynamic process driven by the interplay of cellular and non‐cellular elements of the tumor microenvironment (TME). Among the formers are endothelial cells (ECs), the main elements that drive angiogenesis, a hallmark of tumor progression needed critical for supply of oxygen and nutrients and removal of metabolic waste. Angiogenesis is regulated by various pro‐ and anti‐angiogenic factors, including angiopoietins (ANGPT), a family of cytokines that bind the receptor tyrosine kinase TIE2 on ECs and drive vasculogenesis, vessel homeostasis, and vascular remodeling (Eklund & Olsen, 2006). The best‐known members are Angpt1 and Angpt2. Angpt1 is a Tie2 agonist that triggers receptor autophosphorylation, internalization, and activation of downstream signaling leading to vessel stabilization (Eklund & Saharinen, 2013). In contrast, Angpt2 usually works as Angpt1/Tie2 antagonist and causes endothelium destabilization (Mandriota & Pepper, 1998; Fiedler *et al*, 2004). However, Angpt2 at high concentration and/or in the absence of Angpt1 can function as a Tie2 agonist (Kim *et al*, 2000b; Daly *et al*, 2013). Active Tie2 is phosphorylated at various tyrosine (Tyr) residues. Phosphorylation at Tyr1102 stimulates PI3K and Grb7, which in turn activate focal adhesion kinase p125 (Fak) (Han & Guan, 1999; Kim *et al*, 2000a; Brindle *et al*, 2006). The adaptor Grb2 is also recruited by phosphorylated Tyr1102 and stimulates Erk1/2 (Fukuhara *et al*, 2008). In tumor tissues, ECs secrete high levels of Angpt2, which works as the main Tie2 ligand and together with VEGFA promotes angiogenesis via both autocrine and paracrine mechanisms (Eklund & Saharinen, 2013). Accordingly, elevated circulating levels of Angpt2 have been reported in patients with various solid tumors, including gastro‐enteropancreatic (GEP) NETs (Detjen *et al*, 2010), ovarian cancer (Sallinen *et al*, 2010), and malignant melanoma (Helfrich *et al*, 2009) where they correlate with more advanced disease stage and/or poor prognosis. Interestingly, the biological function and therapeutic potential of angiopoietins in preclinical models of PitNETs or human PitNETs are currently unknown.

## Results

### Circulating ANGPT2 levels in NF‐PitNET patients correlate with tumor aggressiveness

**Figure 1:**
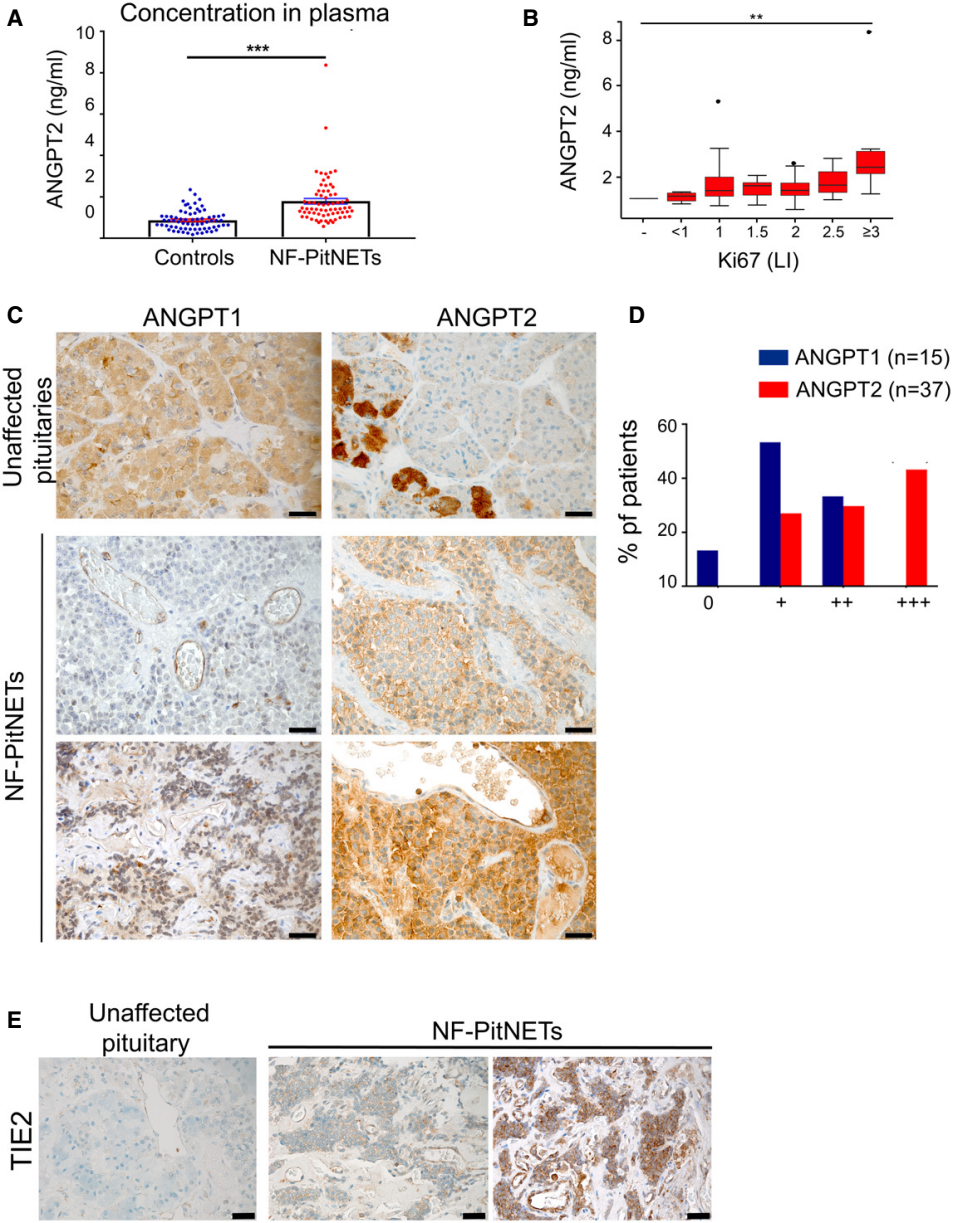
ANGPT2 is elevated in NF‐PitNET patients’ plasma, and is expressed in human primary NF‐PitNETs together with Tie2 and ANGPT1 ANGPT2 plasma concentration in 69 NF‐NF‐PitNET patients and in 69 age‐ and gender‐matched healthy controls. Each dot represents one individual. Healthy controls: mean ± SEM = 0.8698 ± 0.05757; PitNET patients; mean ± SEM = 1.786 ± 0.1383; Difference between means = 0.9158 ± 0.1498; 95% confidence interval = 0.6181 to 1.213. ***P‐value < 0.0001 by unpaired *t*‐test with Welch’s correction.Correlation between circulating ANGP T2 levels and Ki67 LI (i.e., % of Ki67‐positive cells). All box plots show 25^th^ to 75^th^ percentiles (box) and 5^th^ to 95^th^ percentiles (whiskers). The line in the box represents the median. Results are expressed as mean ± SEM. **P = 0.014 by one‐way ANOVA (*P* = 0.007 by Kruskal–Wallis test).Expression of ANGPT1 and ANGPT2 in human unaffected anterior pituitary (control, *n* = 3) and in human NF‐PitNETs. Thirty‐seven human NF‐PitNETs were analyzed for ANGPT2 expression and 14 for ANGPT1. NF‐PitNET panels show representative cases. Original magnification: 400×; scale bar: 20 µm.Summary of the IHC results for human NF‐PitNETs.Expression of Tie2 receptor in human normal pituitary (*n* = 3) and human NF‐PitNET tissues (*n* = 10). Original magnification: 400×; scale bar: 20 µm.

High circulating levels of ANGPT2 have been observed in various human cancers, where they associate with aggressive tumor behaviors and poor prognosis (Helfrich *et al*, 2009; Detjen *et al*, 2010; Sallinen *et al*, 2010). To determine whether a similar situation occurs also in patients with NF‐PitNETs, we used a validated ELISA kit to measure ANGPT2 in the pre‐operative plasma of 69 patients with NF‐PitNETs versus age‐ and gender‐matched healthy controls (*n* = 69). The clinical parameters of the patients are reported in Appendix Table S1. The mean plasma ANGPT2 concentration was 0.87 ng/ml in healthy individuals, and 1.79 ng/ml in patients (*P* < 0.0001) (Fig 1A; Appendix Fig S1A). Circulating ANGPT2 in patients correlated with the Ki67 labeling index (LI) of the tumors (*P* = 0.014; Fig 1B), which represents the most reliable marker of biological behavior in PitNETs (Osamura, 2017), but not with other clinical parameters (Appendix Fig S1B–D). Ki67 immunohistochemistry (IHC) of primary tumors representative of the different labeling index (LI) groups is shown in Appendix Fig S1E.

Following the aforementioned novel finding, we investigated the expression of *ANGPT2* in NF‐PitNET tissues by quantitative (q)RT–PCR. *ANGPT2* expression was elevated (> 2‐fold) in 5/14 samples, including 4/5 invasive tumors (Appendix Fig S2A; Appendix Table S2). For sake of completeness, *ANGPT1* levels were also assessed and found to be significantly reduced in NF‐PitNETs (versus normal tissue) in 13/14 samples (Appendix Fig S2A). Increased *ANGPT2*/*ANGPT1* ratio at the mRNA level has been reported to correlate with neo‐angiogenesis and poor prognosis in many cancer types (Tait & Jones, 2004). In NF‐PitNETs, the *ANGPT2* to *ANGPT1* ratio was shifted toward *ANGPT2* (i.e., > 1) in 13 out of 14 samples (Appendix Fig S2B).

To verify whether changes in gene expression translate into altered protein levels and to determine the cell population expressing these molecules, we performed IHC on human NF‐PitNETs for ANGPT2 (*n* = 37) and ANGPT1 (*n* = 15; Fig 1C and D). In parallel, three unaffected pituitary glands were stained as controls (Appendix Fig S2C). As expected, both angiopoietins were expressed in some endothelial cells (ECs) of both unaffected pituitaries and PitNETs (Fig 1C; Appendix Fig S2C). ANGPT2 was also expressed in the cytoplasm of sparse adenohypophyseal cells in unaffected pituitaries and, more importantly, in tumor cells, where moderate to strong cytoplasmic immunoreactivity was seen in 27/39 cases (Fig 1C and D; Appendix Fig S2C and D). In contrast, cytoplasmic ANGPT1 expression in tumors was weak (*n* = 8) or moderate (*n* = 5), whereas higher levels were observed in adenohypophyseal cells in unaffected pituitaries (Fig 1C and D; Appendix Fig S2C). Rarely, co‐expression of ANGPT1 and ANGPT2 was observed in few tumor cells (Appendix Fig S2E).

In unaffected human pituitary glands, some cells showed positivity for ANGPT2 (Fig 1C). To determine whether they were gonadotroph cells, from which NF‐PitNETs derive, we conducted co‐immunofluorescence (IF) for ANGPT2 and the alpha subunit of gonadotropin hormones (αGSU), a marker of gonadotroph cells, on control pituitary tissue from unaffected individuals (Appendix Fig S2F). The two proteins did not co‐localize. Instead, ANGPT2 was found to co‐localize with the growth hormone (GH), marker of somatotroph cells, another pituitary cell lineage (Appendix Fig S2F). These results indicate that the expression of ANGPT2 is acquired *de novo* by gonadotroph cells during tumorigenesis.

In ECs, angiopoietins exert their function by binding to the TIE2 receptor (Fiedler *et al*, 2004; Eklund & Olsen, 2006; Eklund & Saharinen, 2013). We assessed receptor expression in unaffected human pituitaries and in PitNET tissues. As expected, a positive signal was seen for ECs in control non‐diseased pituitaries and in some tumor‐associated ECs (Fig 1E; Appendix Fig S2C and D). Surprisingly, TIE2 positivity was also observed in the cytoplasm and the plasma membrane of human NF‐PitNET cells (Fig 1E; Appendix Fig S2D), a pattern compatible with published data on HeLa cells with ectopic TIE2 expression (Leppänen *et al*, 2017). Pituitary cells in the control tissues showed no Tie2 staining. The specificity of all antibodies used for IHC and IF was validated on known positive control tissues (Appendix Fig S3A) or on control cells (i.e., rat aortic ECs, RAOECs; Appendix Fig S3B and C).

**Figure EV1:**
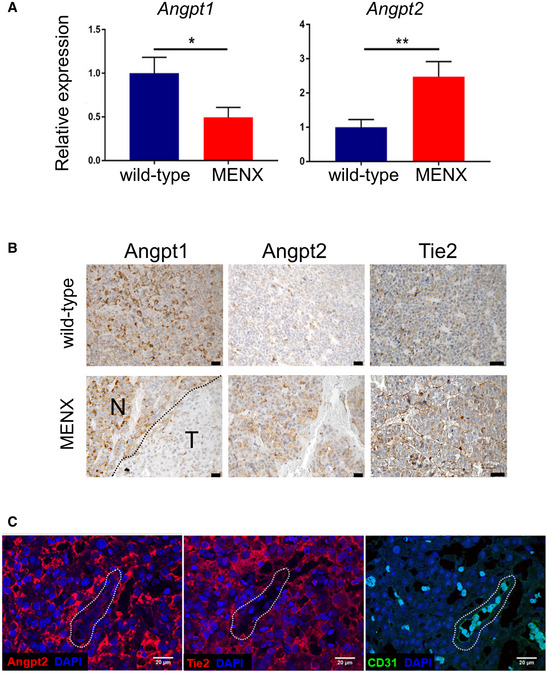
Angpt2 is expressed in rat primary NF‐PitNETs together with Tie2 and Angpt1 qRT–PCR for *Angpt1* and *Angpt2* was performed on pituitary samples from tumor‐bearing MENX mutant rats (*n* = 10, 12, respectively) and wild‐type control rats (*n* = 5, 8). Data are expressed as mean ± SEM. *P = 0.049; **P = 0.008 by *t*‐test.IHC was performed on pituitary tissues from wild‐type (*n* = 5) and MENX mutant rats (*n* = 10) using antibodies against Angpt1, Angpt2. Representative stainings are shown. Original magnification: 400×; scale bar: 20 µm. N, normal area; T, tumor area.Expression of Angpt2, Tie2, and CD‐31 in rat NF‐PitNETs and associated ECs (used as positive control). Consecutive tissue sections of rat NF‐PitNETs (*n* = 4) were stained with the indicated antibodies. One representative tumor is shown. The dashed shape indicates the vessel present in the consecutive slides. Original magnification: 200×; scale bar: 20 µm.

In the laboratory, we work with MENX rats, developing spontaneous, autochthonous NF‐PitNETs. Rat pituitary tumors (frequency 100%) closely resemble human NF‐PitNETs at histo‐pathological and molecular levels (Lee *et al*, 2013; Marinoni *et al*, 2013). We checked whether the rat tumors express angiopoietins and Tie2 receptor similarly to human tumors. Mining previous transcriptome data of rat NF‐PitNETs, we found that *Angpt2* was upregulated (+2.4‐fold), whereas *Angpt1* was downregulated (−3.4‐fold) compared to normal rat pituitary tissues (Lee *et al*, 2013). qRT–PCR confirmed the high levels of *Angpt2* and low levels of *Angpt1* in rat NF‐PitNETs (Fig EV1). We then stained rat pituitaries from wild‐type (WT) rats and MENX tumor‐bearing rats and confirmed that Angpt1 is downregulated whereas Angpt2 is upregulated in the tumors (Fig EV1). Moreover, we found that not only ECs (in both animal groups) but also NF‐PitNET cells express the Tie2 receptor (Fig EV1). IF of consecutive tissue sections of rat PitNET tissues confirmed the expression of both Angpt2 and Tie2 in tumor cells, as well as in CD31‐positive ECs used as the positive control (Fig EV1). Co‐staining of rat tumor tissues for known markers of NF‐PitNETs (Marinoni *et al*, 2013) (i.e., αGSU and the transcription factor SF‐1) and for Tie2 showed that the receptor is co‐expressed with both proteins in the tumor cells (Appendix Fig S4A–F). In contrast, co‐expression of αGSU or SF1 together with Tie2 was not observed in WT rat pituitaries, nor in adjacent non‐tumor areas (Appendix Fig S4C and F), thereby suggesting that Tie2 expression is acquired during tumor progression by the gonadotroph cells. This parallels what we observed in human non‐diseased pituitary samples: ANGPT2 is not present in normal gonadotroph cells (Appendix Fig S2F), but its expression is acquired during tumorigenesis. Thus, altogether, rat NF‐PitNETs express Angpt2 at high levels and further express Tie2.

We also investigated whether rat/human primary NF‐PitNET cultures express Angpt2/ANGPT2 and Tie2/TIE2. Since these cultures contain not only tumor cells but also ECs, which express both molecules, we separated the two cell populations using streptavidin‐coated magnetic beads and a biotinylated anti‐CD31 antibody (Appendix Fig S5A). Of note, primary tumor cultures established from rat and human PitNETs retained the expression of Angpt2/ANGPT2 and Tie2/TIE2 seen in primary tumor tissues (Appendix Fig S5B).

### Endogenous Angpt2 promotes PitNET cell proliferation and survival

**Figure EV2:**
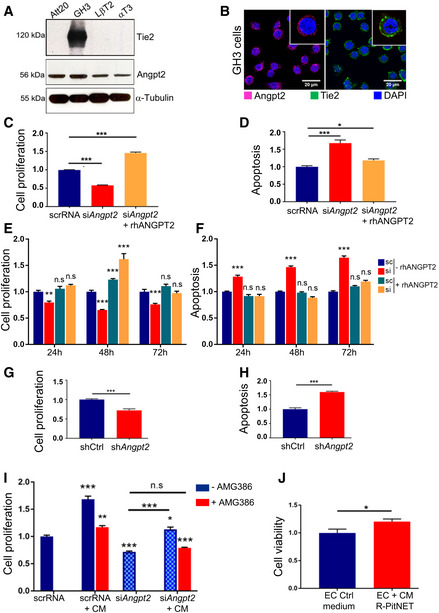
PitNET cells express Angpt2 and Tie2, and intrinsic Angpt2 promotes PitNET cell survival *in vitro* Expression of Angpt2 and Tie2 was assessed in Att20, GH3, LβT2 and αT3 cells by western blotting (WB) using specific antibodies. α‐Tubulin was included as loading control.Immunofluorescence (IF) of GH3 cells for Angpt2 (red) and Tie2 (green). Nuclei were counterstained with DAPI (blue). Original magnification: 400x; scale bar: 20 µm. Panels shown are representative of 3 independent experiments.Cell proliferation of GH3 cells transfected with si*Angpt2* or scrRNA POOLs and incubated with rhANGPT2 (+rhANGPT2) or left untreated (−rhANGPT2) normalized against scrRNA‐transfected cells. ***P < 0.0001 (one‐way ANOVA).In samples parallel to (C), activated caspase‐3/7 was measured to assess for apoptosis. ***P < 0.0001; *P = 0.0411 (one‐way ANOVA).Cell proliferation of GH3 cells transfected with the individual siAngpt2 or scrRNA and incubated with rhANGPT2 (+rhANGPT2) or left untreated (−rhANGPT2) normalized against scrRNA‐transfected, untreated (scr‐rhANGPT2) cells. n.s., not significant; ***P < 0.0001; **P < 0.002 (one‐way ANOVA).In samples parallel to (E), activated caspase‐3/7 was measured to assess for apoptosis. n.s., not significant; ***P < 0.0001 (one‐way ANOVA).Cell proliferation of GH3‐sh*Angpt2* (#2) cells or cells transduced with shCtrl was measured 24h later and normalized against shCtrl. ***P < 0.0001 (*t*‐test).In samples parallel to (G), activated caspase‐3/7 was measured to assess for apoptosis. ***P < 0.0001 (*t*‐test).GH3 cells transfected with si*Angpt2* or scrRNA POOLs were incubated with conditioned medium (CM) from rat primary PitNET cells for 24 h. CM was pre‐incubated with AMG386 (red bars), or left untreated (blue bars). Cell proliferation was measured and normalized against that of scrRNA‐transfected cells. n.s., not significant; ***P < 0.0001; **P = 0.00116; *P = 0.0192 by two‐way ANOVA.Cell viability of isolated rat primary EC cells incubated with CM from isolated rat primary PitNET cells. The experiments was performed independently 2 times, each with 3 technical replicates. Results are expressed as mean ± SEM. *P < 0.0368 (*t*‐test). (C–I) Data are expressed as the mean ± SEM of three biological replicates, each with 3–6 technical replicates. Source data are available online for this figure.

As the function of Angpt2 in PitNETs is unexplored, we set out to investigate its impact on cell proliferation and survival. First, we searched for an *in vitro* model suitable for functional studies. As no human PitNET cell lines, nor NF‐PitNETs lines, currently exist, available rodent pituitary cell lines (GH3, AtT20, LβT2, αT3) were assessed for Angpt2 expression and found to be all positive (Fig EV2). In contrast, only GH3 (PitNET) cells expressed Tie2 (Fig EV2), mainly localized at the plasma membrane as determined by IF (Fig EV2). GH3 cells did not express Angpt1 (Appendix Fig S5C). Antibodies specificity was validated using control ECs for Western blotting (WB; HUVECs, Appendix Fig S5C) and IF (RAOECs, Appendix Fig S3B and C). GH3 cells are not NF‐PitNET cells; however, they are the best available PitNET cell line model as they endogenously express both Tie2 and Angpt2 and thus allow the study of interactions and roles of these proteins in pituitary cells.

To assess the role of endogenous Angpt2 in PitNET cells, we silenced *Angpt2* in GH3 cells transiently with either pools of 30 different si*Angpt2* oligos (siPOOLs; Appendix Table S3), or, to exclude off target effects, with an individual si*Angpt2* oligo having a sequence not included in the siPOOLs. Silencing endogenous *Angpt2* expression in GH3 cells with siPOOLs (Appendix Fig S6A and B) decreased cell proliferation and increased apoptosis (Fig EV2). These effects were rescued by incubation with recombinant human (rh) ANGPT2, thereby confirming that they were Angpt2‐dependent (Fig EV2).

Upon transfection of the individual si*Angpt2* or scramble (scr)RNA in GH3 cells (Appendix Fig S6C), proliferation and apoptosis were assessed at various time points (up to 72 h post‐transfection) in the presence or absence of rhANGPT2 (Fig EV2). These results validated the findings obtained with siPOOLs.

We also knocked down *Angpt2* stably, by transducing GH3 cells with lentiviral vectors containing three different sh*Angpt2* sequences (#1, 2, 3; Materials and Methods), each leading to efficient *Angpt2* knockdown (Appendix Fig S6D). We used cells transduced with sequence #2 (sh*Angpt2*‐GH3) for further *in vitro* and *in vivo* experiments. These cells showed decreased cell proliferation and increased apoptosis *versus* cells transduced with a control unspecific shRNA (shCtrl) (Fig EV2). In conclusion, intrinsic Angpt2 has stimulatory and anti‐apoptotic effects in PitNET cells.

### Tumor‐secreted Angpt2 is bioactive

In order to determine whether the expressed ANGPT2 is also secreted by PitNET cells, we analyzed the released protein in GH3, rat and human primary cultures by ELISA. We detected Angpt2 in the serum‐free, conditioned medium (CM) of GH3 cells (Appendix Table S4). Upon separation of primary tumor cells from primary ECs, as outlined above (Appendix Fig S5A), we found that isolated NF‐PitNET cells from both rat and human cultures secrete Angpt2 (Appendix Table S4). To confirm that PitNET cells indeed secrete Angpt2, we analyzed Angpt2 by WB in CM and cell extracts collected from GH3 cells and HUVECs control cells. Supernatants and lysates from GH3 cells overexpressing ANGPT2 (GH3‐ANGPT2OE) were included as positive controls, as these cells secrete high amounts of Angpt2 into the cell culture medium (Appendix Table S4). A 70 kDa band was detected in the CM of both parental GH3 and GH3‐ANGPT2OE cells by WB, whereas in GH3‐ANGPT2OE cell lysates two bands (56 and 70 kDa) were detected (Appendix Fig S6E). Similarly, two bands were detected in HUVECs lysates (Appendix Figs S5C and S6E). Angiopoietins can be glycosylated (Davis *et al*, 1996). To verify whether the 70 kDa band represents glycosylated Angpt2, we incubated GH3‐ANGPT2OE CM with the enzyme Peptide‐*N*‐Glycosidase F (PNGase), which catalyzes the cleavage of N‐linked oligosaccharides. Upon incubation with PNGAse, the 56 kDa band was detected thereby confirming that the 70 kDa band represents glycosylated Angpt2 (Appendix Fig S6F).

Thus, GH3 cells and primary NF‐PitNET cells not only express Angpt2, but also secrete it.

To prove that Angpt2 secreted by tumor cells is bioactive, we assessed cell proliferation by exposure of scRNA‐ (control) or si*Angpt2*‐transfected GH3 cells (to suppress endogenous Angpt2 secretion) to the serum‐free CM of primary rat NF‐PitNET cells, which predominantly express Angpt2 (Fig EV1). The CM of primary tumor cells significantly increased the proliferation of both scRNA‐ and si*Angpt2*‐GH3 cells (+60% and +57% versus medium alone, respectively; *P* < 0.0001), although silencing *Angpt2* suppressed cell proliferation, as expected (Fig EV2). To confirm that the stimulatory effect of CM was angiopoietin‐dependent, we pre‐incubated CM with an angiopoietin‐neutralizing peptibody (AMG386) before applying it to the cells. The pre‐incubation with AMG386 reduced CM‐dependent stimulation of scRNA‐transfected cells (−17% proliferation *versus* untreated CM; *P* = 0.0012), and completely abolished CM’s effect on si*Angpt2*‐GH3 cells proliferation (n.s. versus untreated CM) (Fig EV2). These results suggest that PitNET cell‐secreted Angpt2 (expressed at much higher levels than Angpt1) may work as a stimulatory factor on tumor cells. Of note, the serum‐free CM of rat NF‐PitNET cells promoted the viability of isolated rat primary ECs (+20%; *P* = 0.036; Fig EV2) indicating that tumor‐borne angiopoietins (in NF‐PitNETs primarily Angpt2) might work as cytokines on ECs within the tumor TME.

**Figure 2:**
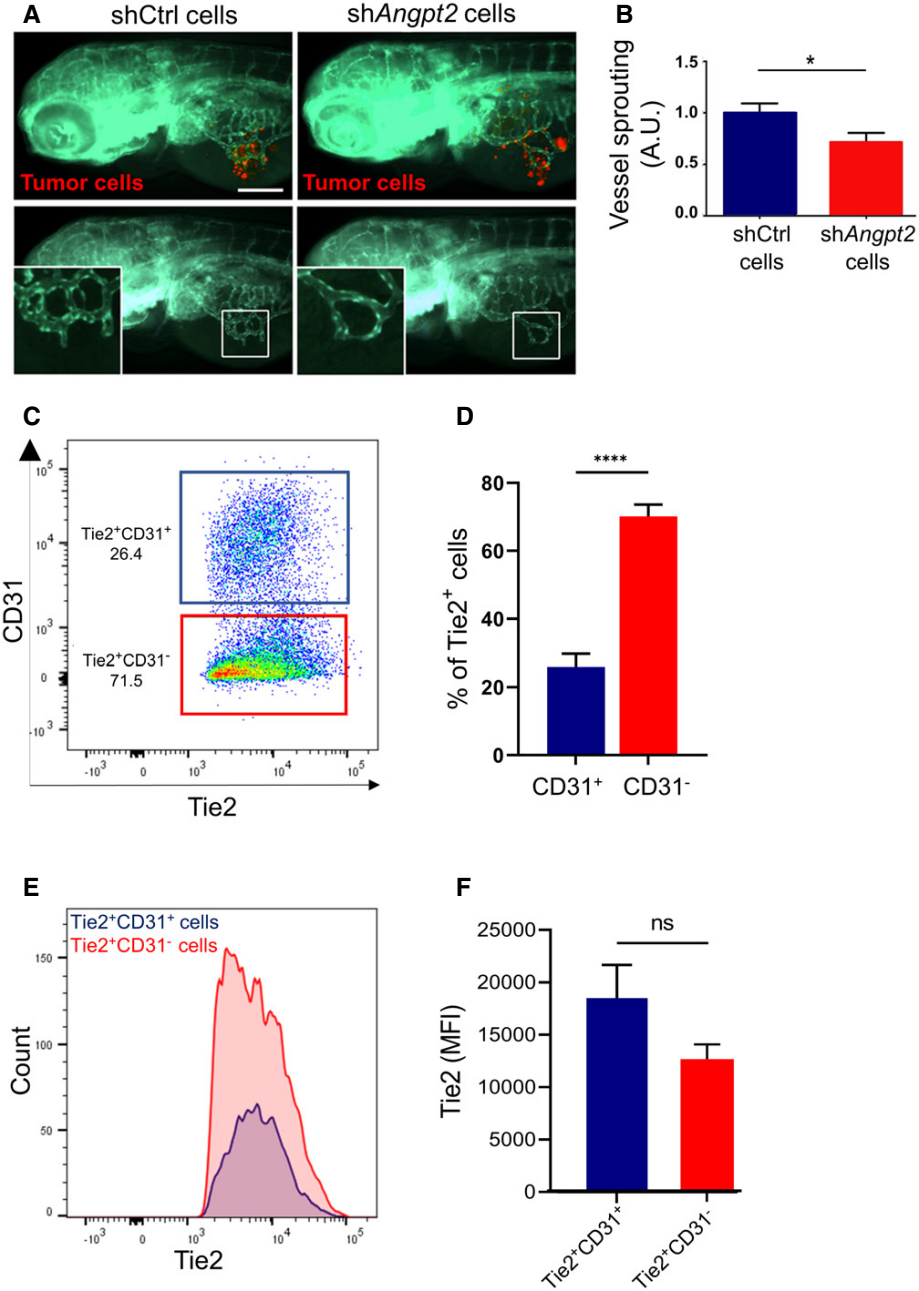
Tumor cells‐secreted Angpt2 induces angiogenesis *in vivo* Zebrafish larvae were implanted with red fluorescence‐stained GH3 cells infected with unspecific shRNA (shCtrl) or with sh*Angpt2* (#2). In the bottom panels, the red channel was omitted to highlight the tumor‐induced microvascular networks. Digital magnifications of graft regions (white boxes) are shown in the bottom left panels. Scale bar: 100 µm.Quantification of tumor‐induced vessel sprouting in zebrafish embryos engrafted with GH3 ‐shCtrl versus shC2 cells (*n* = 20 each). Data were normalized against the mean of the control (shCtrl) group arbitrarily set to 1. A.U. arbitrary Units; *P = 0.0319 (*t*‐test).Example of flow cytometry data of primary rat pituitary cells from a tumor‐bearing MENX rat gated for cell surface Tie2 and CD31 expression (cells were pre‐gated for Tie2).Percentage of Tie2^+^CD31^−^ (PitNET cells) and Tie2^+^CD31^+^ (ECs) in the pituitary glands of 7 age‐matched (8 months) MENX rats. Data are expressed as the mean ± SEM. ****P < 0.0001 (*t*‐test).A representative graph displaying the Tie2 fluorescence intensity and counts of Tie2^+^CD31^−^ and Tie2^+^CD31^−^ cell populations the pituitary of one rat.Fluorescence intensities of the Tie2^+^CD31^−^ and Tie2^+^CD31^−^ cell populations across all 7 pituitary samples. Data are expressed as the mean ± SEM. n.s, not significant (Mann–Whitney test). Source data are available online for this figure.

As EC‐secreted Angpt2 promotes angiogenesis (Eklund & Saharinen, 2013), we investigated whether Angpt2 secreted by tumor cells retains this function by using zebrafish embryos xenografts, an established model to study angiogenesis *in vivo* (Vitale *et al*, 2014). Fluorescently labeled shCtrl‐GH3 cells (control) or sh*Angpt2*‐GH3 cells were injected into the sub‐peridermal space of embryos. Forty‐eight hours later the total cumulative length of vessels sprouting from subintestinal and common cardinal veins toward the tumor cells was measured by high‐resolution live imaging (Fig 2A). sh*Angpt2*‐GH3 cells induced less vessel sprouting than control cells (−30%, *P* = 0.0319; Fig 2B), thereby demonstrating that Angpt2 secreted by tumor cells retains the pro‐angiogenic properties of EC‐secreted Angpt2.

### Tumor cell‐bound Tie2 is functional

The unanticipated discovery that NF‐PitNET cells express Tie2 on their plasma membrane opens a new scenario: if the receptor is functional (i.e., can interact and be activated by its ligands), then it could respond to angiopoietins present in the TME (secreted by both tumor and endothelial cells).

To verify that indeed NF‐PitNET cells express Tie2 on their plasma membrane, we conducted flow cytometry analysis on freshly isolated primary cells from whole pituitary glands of MENX rats (*n* = 7) at the age when they all have large, multifocal NF‐PitNETs (= 8–9 months) (Marinoni *et al*, 2013). Staining was done using antibodies against Tie2 and CD31 (marker of ECs). Only antigens exposed on the cell membrane were detected using a non‐permeabilizing staining protocol. We identified Tie2^+^/CD31^+^ ECs, as expected, as well as Tie2^+^/CD31^−^ tumor cells (Fig 2C and D; Appendix Fig S7A). The identity of the cells was confirmed by qRT–PCR on sorted cells using a marker for ECs (*Pecam1*), and, for the rat tumors cells, two genes (i.e., *Lyn* and *Id2)* previously reported to be overexpressed in these cells (Lee *et al*, 2013) (Appendix Fig S7B). Tie2^+^ cells in MENX rat pituitaries were mostly (average 70.14%) CD31^−^ (= tumor cells), while the remaining cells (average 25.94%) were CD31^+^ (= ECs). The fluorescence intensity of Tie2 in both populations was overlapping (Fig 2E and F). This experiment confirmed that Tie2 is exposed on the plasma membrane of NF‐PitNET cells.

As outlined above, NF‐PitNET cells express high levels of Angpt2 and secrete it as bioactive cytokine, whereas they express *Angpt1* at low level. Moreover, knockdown of *Angpt2* showed that this protein plays a pro‐proliferative role in these cells. Thus, we focused on the functional relation between Angpt2 and Tie2.

**Figure 3:**
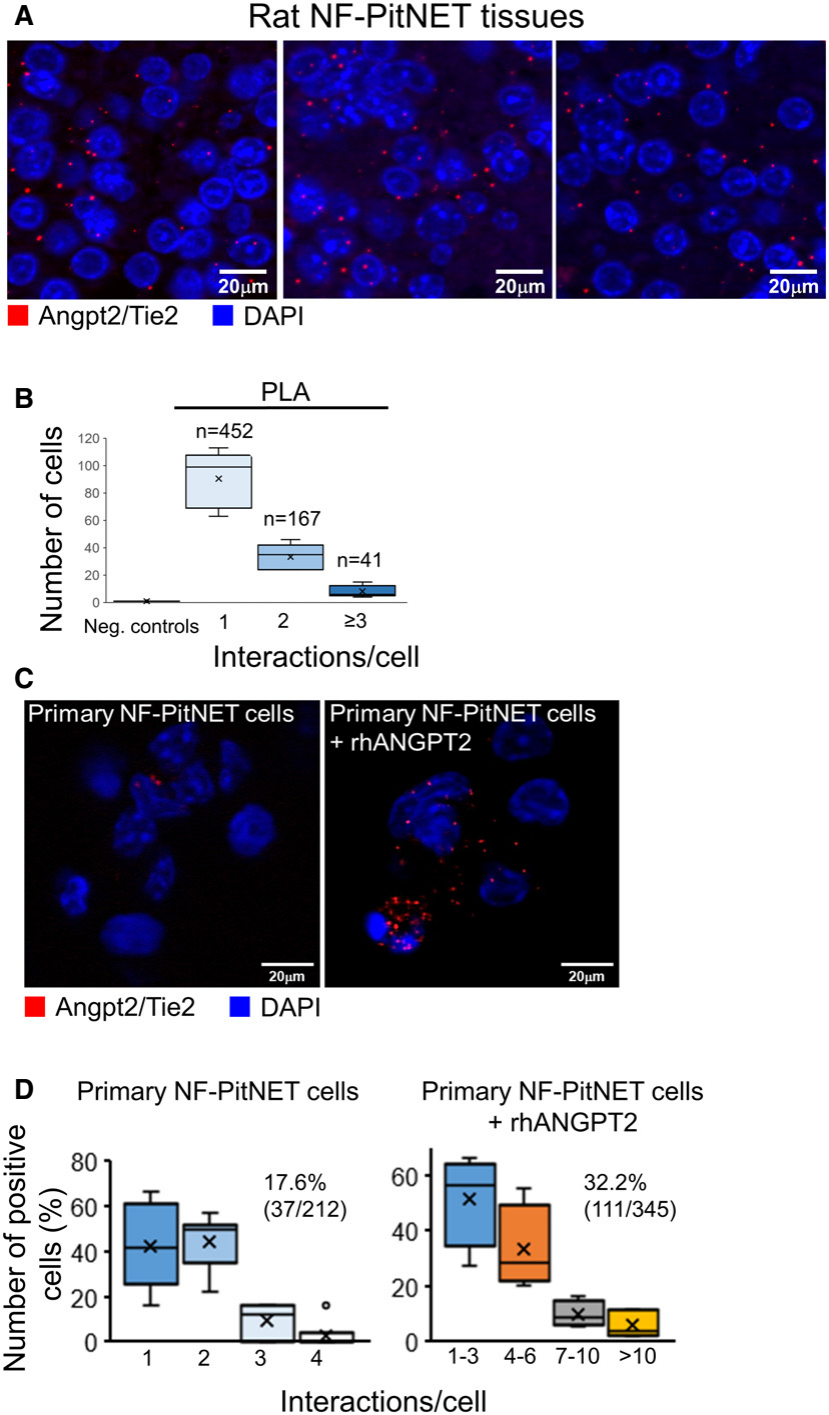
Tie2 on PitNET cells interacts with Angpt2 Proximity ligation assay (PLA) was performed on FFPE sections of rat PitNETs (*n* = 5) using antibodies against both Angpt2 and Tie2. Nuclei were counterstained with DAPI (blue). Original magnification: 400×; scale bar: 20 µm.Quantification of the interactions between Angpt2 and Tie2 in rat PitNET tissues versus negative controls obtained using only 1 antibody (Appendix Fig S7C). Box plots show 25^th^ to 75^th^ percentiles (box) and 5^th^ to 95^th^ percentiles (whiskers). The line in the box represents the median, whereas the “x” represents the mean.PLA was performed as in (A) on isolated rat primary NF‐PitNET stimulated with rhANGPT2.Quantification of Angpt2/Tie2 interactions in primary NF‐PitNET incubated with/without rhANGPT2. The total number of cells counted is reported in parenthesis (i.e., 212 and 345) as well as the number of positive cells (= showing at least one interaction). The graphs show the percentage of cells having the number of interactions reported on the x axis. Box plots show 25^th^ to 75^th^ percentiles (box) and 5^th^ to 95^th^ percentiles (whiskers). The line in the box represents the median, the “x” represents the mean, and the circle outlier points. Data information: (C, D) Pictures were taken with the same exposure time. Results shown are representative of the stainings results across all samples (*n* = 4, *n* = 3, respectively). Source data are available online for this figure.

To verify whether tumor‐bound Tie2 can interact with Angpt2, as it occurs for EC‐bound Tie2, we employed proximity ligation assay (PLA) on rat NF‐PitNET tissues using antibodies against Angpt2 and Tie2. The anti‐Tie2 antibody was raised against the extracellular domain of the receptor. PLA signals were detected only using the two antibodies together (Fig 3A), whereas no signal was detected employing each primary antibody alone (negative controls; Appendix Fig S7C). Discrete PLA spots were counted on rat tumor tissue sections. The results showed that 34.5% of the tumor cells counted had at least one positive interaction between Tie2 and Angpt2, with 68.5% of the positive cells showing only one interaction (Fig 3B). We hypothesized that this low number of interactions was likely due to the variable amount of Angpt2 in the TME and, possibly, to tissue processing. To further explore the interaction between Tie2 and Angpt2 in NF‐PitNET cells, PLA was also conducted on isolated primary NF‐PitNETs from 9‐month‐old MENX rats with very large pituitaries (*n* = 3). Pituitary glands were processed, single cells obtained, and depleted of ECs using CD31‐coated beads. CD31‐ cells (mainly NF‐PitNET cells) in serum‐free medium were incubated with rhANGPT2 for 15 min or left untreated, and then immediately processed for PLA. In the presence of the ligand, isolated primary CD31‐ cells showed abundant Angpt2‐Tie2 interactions, distributed mainly on the cell surface (Fig 3C, quantified in 3D; Appendix Fig S8A). ANGPT2 is not internalized together with Tie2 in ECs (Bogdanovic *et al*, 2006). Thus, PLA detects the interaction between the added hANGPT2 and Tie2 on the plasma membrane of tumors cells. To verify that the antibodies used for PLA assays were suitable to detect the interaction between the two proteins, RAOECs were analyzed in parallel and also showed interactions between ligand and receptor, occurring mainly at the cell membrane as expected (Appendix Fig S8B and C). Altogether, NF‐PitNET cells possess a Tie2 receptor on their plasma membrane, which is able to bind to Angpt2.

In ECs, binding of Tie2 to Angpt2 (at high concentration) promotes receptor phosphorylation, and internalization (Mandriota & Pepper, 1998). In agreement with the hypothesis that Tie2 on PitNET cells is functional and can be activated by its ligands, we found that the receptor is in part phosphorylated in tumor cells. Primary rat/human PitNET cells showed immunoreactivity to the anti‐P‐Tie2 (Tyr1102/1108) antibody, suggesting that they possess a partially phosphorylated Tie2 (Figs EV1 and EV2; Appendix Fig S9A). Control HUVECs were used to validate the specificity of the anti‐P‐Tie2 antibody (Appendix Fig S9A).

**Figure EV3:**
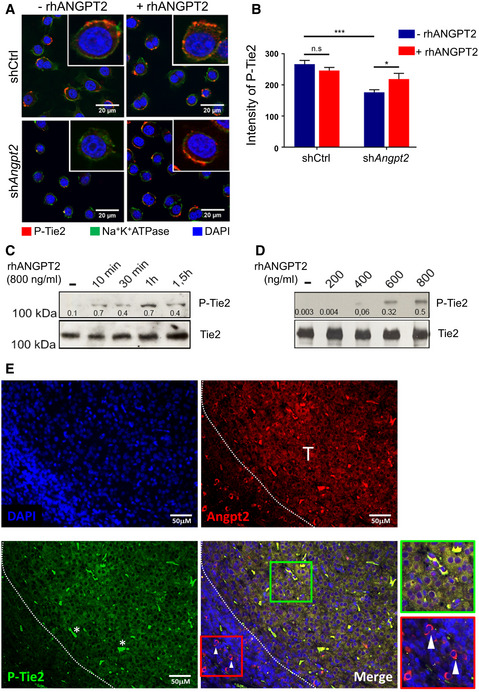
Tie2 on PitNET cells is phosphorylated and can be stimulated by Angpt2 Co‐IF for both P‐Tie2 (Tyr 1102/1108; red) and for Na^+^K^+^ ATPase (green), used as plasma membrane marker, of GH3 cells transduced with unspecific shRNA (shCtrl) and sh*Angpt2* (#2) and incubated with rhANGPT2 or left untreated. Original magnification: 400×; scale bar: 20 μm.Quantification of P‐Tie2 immunostaining intensity in cells shown in (A) shCtrl‐transduced GH3 cells (266.71 ± 12.12); in sh*Angpt2* cells (176.04 ± 8.46); in control cells treated with rhANGPT2: shCtrl + rhANGPT2: 246.33 ± 9.79 (versus shCtrl, not significant by *t*‐test); in treated knockdown cells: sh*Angpt2* + rhANGPT2: 218.68 ± 18.16 (versus shC2; *P = 0.0421 by *t*‐test). The experiment was performed twice each with three technical replicates. Intensities are expressed as arbitrary units ± SEM. ***P < 0.0001 by *t*‐test.Tie2 and P‐Tie2 expression in serum‐starved GH3 cells transfected with siAngpt2 POOLs and stimulated with rhANGPT2 for the indicated times.Tie2 and P‐Tie2 expression in cells as in (C) stimulated with the indicated doses of rhANGPT2 for 30 min.Co‐IF for Angpt2 (red) and P‐Tie2 (green) of a representative rat primary NF‐PitNET. Nuclei were counterstained with DAPI. White arrows point to Angpt2‐positive (P‐Tie2 negative) cells in adjacent non‐tumor area. Scale bars: 50 µm. T, tumor area. Data information: (C, D) Blots shown in all panels are representative of three independent experiments. The numbers represent the ratio phospho/total proteins. Source data are available online for this figure.

Interestingly, the downregulation of endogenous Ang2 (= sh*Angpt2*) in GH3 cells was associated with decreased P‐Tie2 levels (versus parental cells; Fig EV3). This suggests that endogenous Angpt2 secreted by the tumor cells contributes to the activation of tumor cell‐bound Tie2. When sh*Angpt2*‐GH3 cells were treated with rhANGPT2, Tie2 phosphorylation increased (Fig EV3) whereas no increase in P‐Tie2 was seen in shCtrl‐transduced GH3 cells (control). This is likely due to the fact that Angpt2 secreted in the media by the tumor cells activates the receptor located on their plasma membrane, thereby making these cells not responsive to the recombinant protein (Fig EV3).

Although GH3 cells do not express detectable levels of Angpt1, we stimulated them also with rhANGPT1 to verify whether the Tie2 receptor in these cells can be activated by its canonical ligand. We found that Tie2 phosphorylation increased upon treatment with rhANGPT1 (Appendix Fig S9B and C).

To confirm that Angpt2 acts as agonist of tumor cells‐bound Tie2, we assessed receptor phosphorylation upon stimulation also by WB. In parental GH3 cells, incubation with rhANGPT2 in serum‐free medium did not increase Tie2 phosphorylation, possibly because the receptor was activated by endogenous Angpt2 and thus not responsive to rhANGPT2 (as seen in Fig EV3). To circumvent this issue, we silenced endogenous *Angpt2* expression. GH3 cells (in serum‐free medium) were transfected with si*Angpt2* and then incubated with increasing concentrations of rhANGPT2 for 30 min, or with 800 ng/ml rhANGPT2 for 10–90 min, and then Tie2 phosphorylation was assessed. Treatment with rhANGPT2 led to dose‐ and time‐dependent Tie2 phosphorylation (Fig EV3).

We then assessed whether Tie2 is phosphorylated in NF‐PitNET tumor tissues. To this aim, we performed immunofluorescence on rat pituitaries with a phosphor‐specific antibody. Albeit staining tumor tissues with anti‐P‐Tie2 antibodies has technical limitations, we found that P‐Tie2 and Angpt2 co‐localize in the tumors, whereas in adjacent non‐tumor areas Angpt2‐positive pituitary cells do not express P‐Tie2 (Fig EV3; Appendix Fig S10A and B). We then determined the expression of P‐Tie2 by WB in a more representative model of NF‐PitNETs, that is, isolated rat primary tumor cells. We separated tumor cells and ECs from primary rat tumors (*n* = 4) as reported above, and we stimulated the pooled cells with rhANGPT1 and rhANGPT2. In parallel, RAOECs, stimulated with rhANGPT1 or left untreated, were also analyzed (Appendix Fig S11A). We found that, in primary rat tumor and endothelial cells, P‐Tie2 expression is stimulated by both angiopoietins (Appendix Fig S11B).

We have shown so far that Tie2 in PitNET cells can be stimulated by recombinant angiopoietins. Next, we wondered whether Tie2 on tumor cells can also be activated by tumor cell‐secreted angiopoietins. Thus, serum‐free CM of isolated rat primary PitNET cells (devoid of ECs) was added to sh*Angpt2*‐GH3 cells and Tie2 phosphorylation was assessed by IF. Tumor cell‐derived CM increased the levels of P‐Tie2 (Appendix Fig S12A and B). As Angpt1 is expressed at very low levels by PitNET cells (Fig EV1), we speculate that receptor activation is mostly mediated by Angpt2 present in the CM.

In ECs, activated Tie2 stimulates downstream signaling pathways including Fak, PI3K/AKT, and Erk1/2 signaling cascades (Han & Guan, 1999; Kim *et al*, 2000a; Harfouche & Hussain, 2006; Hu *et al*, 2006; Fukuhara *et al*, 2008). In parallel to receptor activation, rhANGPT2 treatment of si*Angpt2*‐GH3 cells increased the phosphorylation of Fak (Y397) and Erk1/2 (Thr202/Tyr204) in a dose‐ and time‐dependent manner (Appendix Fig S13A and B). To confirm these data, similar experiments were also performed by pre‐incubating GH3 cells (in serum‐free medium) with the angiopoietin‐neutralizing peptibody AMG386 (Appendix Fig S13C). Twenty‐four hours later, cells were incubated for 30 min with varying amounts of rhANGPT2. Control cells were collected at the 30 min time point to monitor for potential effects of newly secreted Angpt2. Also with this experimental setup, we observed activation of Fak and Erk1/2 signaling upon Tie2 stimulation (Appendix Fig S13C). Thus, Angpt2 is a partial agonist of tumor‐associated Tie2 and causes its phosphorylation and then the activation of Fak and Erk1/2, as previously demonstrated in ECs (Bogdanovic *et al*, 2006).

### Tumor cell‐bound Tie2 mediates pro‐mitogenic signals in PitNETs *in vivo*

**Figure 4:**
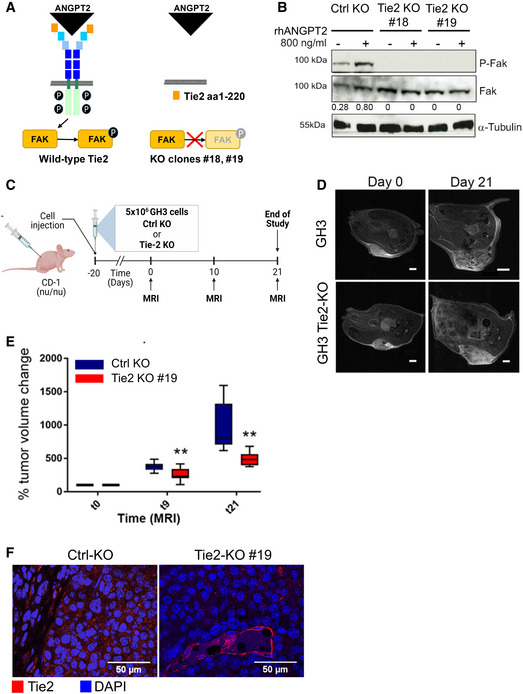
Knockout of Tie2 in PitNET cells suppresses tumor growth *in vivo* Scheme of the wild‐type Tie2 receptor (left), and the receptor domain left‐over in the targeted clones #18 and #19 (right) and the impact of the mutation on the activation of downstream signaling.Expression of total Fak and P‐Fak (Y397) in serum‐starved GH3 Ctrl‐KO and Tie2‐KO clones #18 and #19 stimulated with rhANGPT2 for 30 min or left untreated. The numbers represent the ratio phospho/total Fak. Anti‐α‐tubulin antibody was used to check for equal loading. Blot shown is representative of 3 independent experiments.Scheme of the *in vivo* study in mouse xenografts of GH3 Ctrl‐KO and Tie2‐KO cells (clone #19).T2‐weighted images of two xenografted tumors taken at day 0 and 21 (largest diameter) representing the two animal groups. Scale bar: 2 mm, except GH3‐Ctrl KO day 21: 4 mm.Changes in tumor volumes as determined by MRI were normalized to the day 0 value (=100%) for each animal. All box plots show 25^th^ to 75^th^ percentiles (box) and 5^th^ to 95^th^ percentiles (whiskers). The line in the box represents the median. **P = 0.003 (one way ANOVA).Expression of Tie2 (red) in the xenografted tumors (*n* = 3 each group). Nuclei were counterstained with DAPI (blue). Original magnification: 400×; scale bar: 50 µm. Source data are available online for this figure.

To confirm the role of tumor cell‐bound Tie2 in mediating pro‐mitogenic signals *in vivo,* we generated Tie2‐defective GH3 cells by CRISPR‐Cas9‐mediated gene knockout (KO) for xenograft experiments. Of the selected clones, two (#18 and #19) had a homozygous 1‐base insertion leading to a stop codon at amino acid 220 (Appendix Fig S14A and B). Consequently, these Tie2‐KO PitNET cells have no functional receptor and display no downstream signal activation (i.e., no increase in P‐Fak) upon stimulation with rhANGPT2 (Fig 4A and B). We engrafted GH3 (Ctrl‐KO) or Tie2‐KO GH3 cells (clone #19) subcutaneously into the flanks of immunodeficient mice (*n* = 8 per group; Fig 4C). Twenty days after cell transplantation (average tumor volume 50 mm^3^), high‐resolution anatomical magnetic resonance imaging (MRI) was performed (= day 0), and then again 10 and 21 days later to longitudinally monitor tumor growth (Fig 4C–E). The results showed that deletion of endogenous Tie2 expression significantly suppressed PitNET cell growth *in vivo* (Fig 4E). *Ex vivo* analyses of excised tumors derived from Ctrl‐KO or Tie2‐KO injected cells confirmed the lack of Tie2 expression in the latter, with mouse ECs serving as positive control (Fig 4F). In conclusion, abrogation of Tie2 function in this PitNET cell line reduced their growth *in vivo* likely because it impaired their response to angiopoietins present in the TME.

### Targeting Ang/Tie2 signaling in PitNETs *in vitro* and *in vivo*

**Figure EV4:**
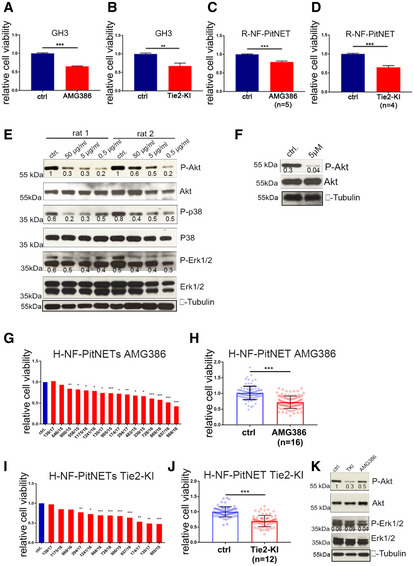
Pharmacological Ang/Tie2 pathway inhibition reduces the viability of PitNET cell lines, and of rat/human primary cultures *in vitro* A, BCell proliferation of GH3 cells treated with 5 μg/ml AMG386 (A) or 5 μM Tie2‐KI (B) or left untreated (ctrl) normalized against untreated cells. ***P < 0.0001; **P = 0.0072 (*t*‐test). Results are expressed as mean ± SEM of three biological replicates with 6 technical replicates.C, DCell viability of rat primary PitNET cells (R‐PitNET) treated with AMG386 (*n* = 5 rats) (C) or Tie2‐KI (*n* = 4 rats) (D) or left untreated (ctrl) normalized against untreated cells. Primary cultures were treated, each with 6 technical replicates. Results are expressed as mean ± SD. ***P < 0.0001 (*t*‐test).EExpression of phosphorylated (P) and total Akt, p38 and Erk1/2 in two R‐PitNET cultures with enough cells upon treatment with AMG386.FExpression of phosphorylated (P) and total Akt in one R‐PitNET culture treated with Tie2‐KI or left untreated (ctrl).G–JCell viability of human primary PitNET cultures treated with (G, H) AMG386 (*n* = 16) or (I, J) Tie2‐KI (*n* = 12) normalized against untreated control cells for each patient (set to 1). Significance in (G, I): *P < 0.05; **P < 0.01; ***P < 0.001 (by *t*‐test, comparing each treated sample with its untreated control). Results in (H) and (J) are expressed as mean ± SEM of the normalized values of 6 technical replicates for each samples. Each dot represents the relative cell viability of one technical replicate. Significance in (H, J) ***P < 0.0001 (*t*‐test).KExample of responsive H‐PitNET treated with AMG386 or Tie2‐KI. Total proteins were extracted and probed for P‐ and total Akt and Erk1/2. Anti‐α‐tubulin antibody as used to check for equal loading. The blot shown is representative of three independent cultures. Data information: (E, F) Blots shown are representative of 3 independent experiments. (E, F, K) The numbers below the bands represent the ratio phospho/total proteins. Source data are available online for this figure.

As Angpt2/Tie2 signaling activates pro‐mitogenic pathways and promotes PitNET cells proliferation/viability, its pharmacological blockade is expected to antagonize these tumors. To test this hypothesis, we evaluated the efficacy of both AMG386 and Tie2‐KI against GH3 cells *in vitro*: both drugs significantly (> 20%) inhibited proliferation (Fig EV4). Similar results were obtained in rat primary PitNET (R‐PitNET) cells (Fig EV4). At the molecular level, we observed a decrease in P‐Akt, P‐p38, and P‐Erk1/2 following AMG386 treatment of primary rat PitNET cells, and reduction of P‐Akt upon Tie2‐KI treatment (Fig EV4).

We also assessed the response of primary cultures of human NF‐PitNETs. Eleven of 16 primary cultures were responsive to AMG386 (significance was observed starting at a −20% reduction in cell viability) (Fig EV4). The range of suppression of cell viability was −20 to −60%. Twelve samples could also be tested with Tie2‐KI and 9 were responsive (Fig EV4). Reduction of cell viability upon Tie2‐KI treatment ranged from −20 to −55%. Overall, eight primary cultures responded to both drugs, two responded to only one of them, and two did not respond to either one. We validated the expression of *ANGPT2* and *Tie2* in some primary cultures by qRT–PCR (Appendix Fig S15). WB analysis of few cultures with enough material showed reduced P‐Akt and P‐Erk1/2 expression after drug treatment (Fig EV4).

**Figure 5:**
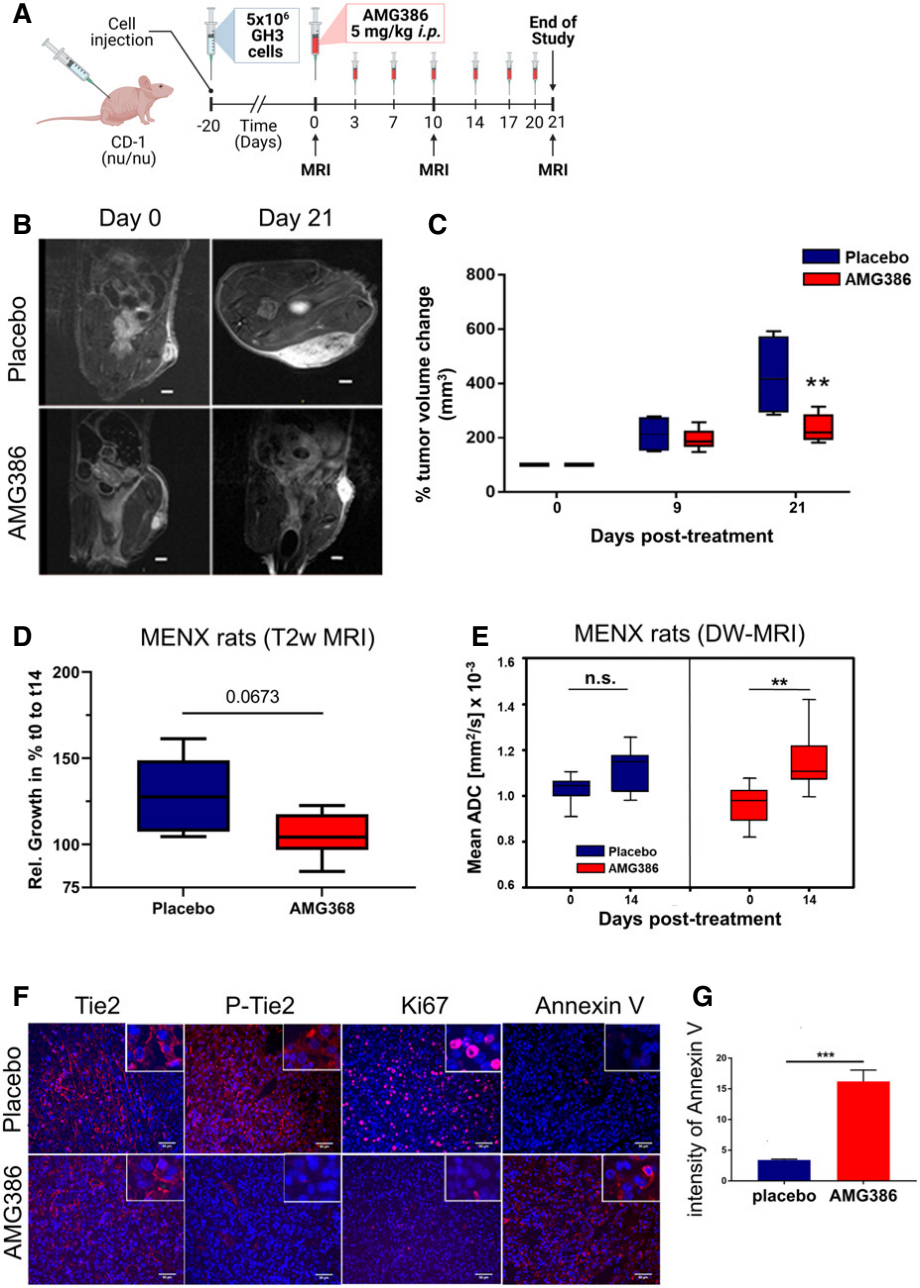
Pharmacological inhibition of Ang/Tie2 signaling suppresses growth and induces apoptosis of PitNET cells xenograft models and of autochthonous PitNETs in MENX rats *in vivo* Scheme of the *in vivo* treatment of GH3‐derived xenografts with AMG386.T2‐weighted images of two xenografted tumors taken at day 0 and 21 (largest diameter). The tumors are representative of the two treatment groups Scale bar: 2 mm.Changes in tumor volumes as determined by MRI were normalized to the day 0 value (= 100%) for each animal. All box plots show 25^th^ to 75^th^ percentiles (box) and 5^th^ to 95^th^ percentiles (whiskers). The line in the box represents the median. **P = 0.007 (one way ANOVA).Changes in tumor volumes in female rats treated with AMG386 (*n* = 6) or with placebo (*n* = 5) for 14 days as determined by anatomical MRI, normalized to the day 0 value. Box plots show 25^th^ to 75^th^ percentiles (box) and 5^th^ to 95^th^ percentiles (whiskers). The line in the box represents the median. *P* = 0.0673, not significant (one way ANOVA).ADC values of the rat PitNETs before (day 0) and after (day 14) treatment with AMG386 (red, *n* = 8) or placebo (blue, *n* = 5). Box plots show 25^th^ to 75^th^ percentiles (box) and 5^th^ to 95^th^ percentiles (whiskers). The line in the box represents the median. **P = 0.009; n.s., not significant (*t*‐test).
*Ex vivo* expression of Tie2, P‐Tie2, Ki67 and Annexin V in PitNETs of female rats treated with AMG386 or placebo for 14 days. Original magnification: 200×; scale bar: 50 µm. Panels shown are representative of the two animal groups.Quantification of Annexin V intensity from tissues stained as in (F) (*n* = 4/group). Three different areas per tumor sample were analyzed. Intensities are expressed as arbitrary units ± SEM. ***P < 0.001 (by *t*‐test). Source data are available online for this figure.

Based on the promising *in vitro* effects of Ang/Tie2 pathway inhibition, we then assessed its effect on GH3 cell‐derived mouse xenografts *in vivo*. We selected AMG386 as representative of specific anti‐angiopoietin biologics currently in clinical trials. Twenty days after cell transplantation (average tumor volume 50 mm^3^), nude mice were randomized and treated with AMG386 (*n* = 8) or placebo (*n* = 4) 2×/week intraperitoneally for 3 weeks (Fig 5A). Measurement of tumor growth by longitudinal, high‐resolution MRI was performed at day 0, 10 and 21 post‐treatment (Fig 5A and B). AMG386 significantly suppressed tumor cell growth *in vivo* (Fig 5C). Staining of tumors *ex vivo* showed a prominent increase in Annexin V (apoptosis) in drug‐treated versus placebo‐treated tumors (Appendix Fig S16A and B).

We also determined the effect of AMG386 on spontaneous, autochthonous NF‐PitNETs developing in MENX rats (Leppänen *et al*, 2017). In a pilot short‐term study (3‐day treatment) (Appendix Fig S17A), we verified that AMG386 reaches the pituitary gland and blocks Ang/Tie2 signaling in this model. While all rat PitNETs showed immunoreactivity for Tie2, tumors of placebo‐treated, but not of drug‐treated, rats were positive for P‐Tie2 (Appendix Fig S17B). While acknowledging possible technical limitations, these staining results make P‐Tie2 a useful readout of drug response. Ki67, but not Annexin V (apoptosis), positivity decreased already after 3 days of AMG386 treatment (Appendix Fig S17B).

Based on these results, we conducted a study where MENX rats were treated with AMG386 (*n* = 12) or placebo (*n* = 5) for 2 weeks (Appendix Fig S17C). High‐resolution anatomical magnetic resonance imaging (MRI) and diffusion‐weighted (DW) MRI with were performed on a subset of mutant rats at day 0 and 14 after AMG386 (*n* = 8) or placebo (*n* = 5) treatment. Calculated mean apparent diffusion coefficient (ADC) values obtained by DW‐MRI were used to assess changes in tumor cellularity as surrogate marker of therapy response (Trajkovic‐Arsic *et al*, 2017). During the course of the experiment, we observed a trend toward a slower tumor growth in AMG386‐treated MENX rats *versus* placebo‐treated animals, as determined by MRI, which was however not statistically significant (Fig 5D). The fact that a relatively short treatment regimen (14 days) was able to elicit a reduction in tumor growth detectable by neuroimaging is remarkable. ADC values significantly increased after AMG386 treatment (*P* = 0.009) but not following placebo administration (*P* = 0.177), again supporting the anti‐tumor effect of the drug in this autochthonous model (Fig 5E). *Ex vivo* analysis of PitNET tissues confirmed a reduction in P‐Tie2 expression in drug‐ but not in placebo‐treated tumors, while PitNETs of all rats showed immunoreactivity for Tie2 (Fig 5F). The Ki67 LI was drastically reduced in drug‐treated *versus* placebo‐treated tumors (7.5 ± 0.81% versus 24.53 ± 3.24%, respectively; *P* = 0.0005), whereas Annexin V expression was enhanced in the former (versus placebo; Fig 5F and G).

Altogether, Ang/Tie2 inhibition elicited strong anti‐proliferative and pro‐apoptotic responses in PitNETs *in vivo*. DW‐MRI, indirectly assessing cell death, emerged as a suitable imaging modality for early response monitoring following anti‐angiopoietin/Tie2 therapy in autochthonous PitNETs.

## Discussion

**Figure 6:**
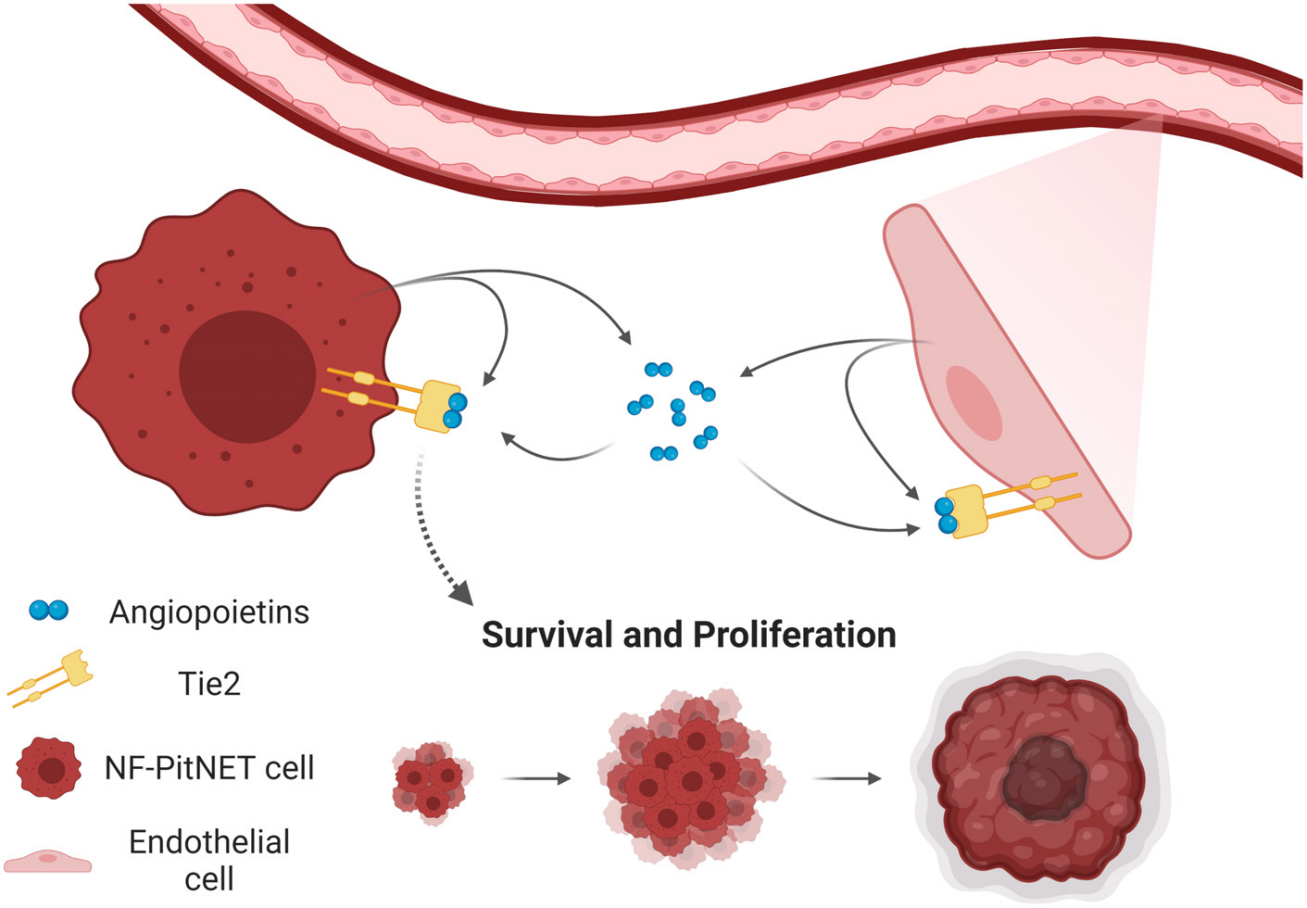
Tumor/endothelial cells crosstalk in the tumor microenvironment (TME) Pituitary tumor cells secrete mostly Angpt2 in the TME. Tumor‐associated ECs secrete mostly Angpt2 but also Angpt1. Thanks to the expression of Tie2 on their plasma membrane, tumor cells can respond to angiopoietins (secreted by both tumor and endothelial cells) and activate pro‐proliferative/pro‐survival pathways.

Our studies demonstrate that NF‐PitNET cells coopt angiogenic pathways typical of ECs (i.e., Angpt2/Tie2) and repurpose them to support their own growth and survival. The data presented here support the existence of autocrine/paracrine signaling involving angiopoietins (mainly Angpt2) in NF‐PitNET cells (Fig 6).

We discovered that Angpt2 is highly expressed in NF‐PitNET cells, which also secrete it as a bioactive cytokine. Tie2 receptors on tumor cells can bind Angpt2 and this initiates stimulatory autocrine signaling involving the same downstream effectors seen in ECs (e.g., Fak, ERK1/2). Activation of these effectors promotes tumor cell proliferation/viability and decreases apoptosis in established and primary *in vitro* PitNET models. Tumor‐borne Angpt2 promotes angiogenesis in zebrafish embryos *in vivo* and works in a paracrine manner on ECs; this implies that Angpt2 might play an active role within the TME in the crosstalk between tumor cells and ECs. Thus, Angpt2 joins the array of growth factors and cytokines that tumor cells release to regulate angiogenesis, foster EC survival and ultimately promote progression. Rat and human primary NF‐PitNET cells/tissues express Angpt1/ANGPT1 but at low level, whereas they express Angpt2/ANGPT2 at high level. This observation, together with the effects caused by Angpt2/ANGPT2 downregulation, point to a major role of this cytokine in tumor/ECs crosstalk.

As previously only shown for melanoma cells (Helfrich *et al*, 2009), our findings that NF‐PitNET cells express both the *Tie2* gene and a functional cell membrane‐bound receptor (as demonstrated by flow cytometry and by stimulation experiments) broadens our view of the bidirectional signaling crosstalk in which tumor and endothelial cells engage. Indeed, endogenous Tie2 allows tumor cells to use angiopoietins present in the tumor milieu, derived from both tumor cells and ECs, thereby generating a symbiotic intratumoral signaling pathway (Fig 6). The relevance of tumor cell‐intrinsic Tie2 receptor signaling in pituitary tumorigenesis was demonstrated by transplantation studies where Tie2‐defective PitNET cells were injected into immunocompromized mice and compared with Tie2‐expressing control cells. Tie2 deficiency significantly suppressed tumor growth *in vivo*, thereby proving that there is a cell‐autonomous role of angiopoietin/Tie2 signaling relevant for PitNET cell tumorigenesis. The importance of an active Tie2 receptor in NF‐PitNET progression is further underscored by two findings: (i) sequestration of angiopoietins in the TME with AMG386 blunts PitNET growth in mouse xenograft models; (ii) AMG386 treatment suppresses NF‐PitNETs growth in a representative autochthonous tumor model (MENX rats) where the physiological heterocellular communication in the TME is preserved. Remarkably, NF‐PitNETs in MENX rats showed virtually no Tie2 activation after only 3 days of AMG386 treatment, which led to a dramatic decrease in tumor cell proliferation and increase in apoptosis in the 14‐day treatment regimen. Consequently, and compared to placebo‐treated rats, treatment with AMG386 for 14 days was associated with limited tumor growth. This is clinically relevant since any ability to stop tumors from growing may benefit patients with recurrent/remnants of NF‐PitNETs who are unsuitable for surgery. Moreover, if we consider that treatment of MENX rats with a cytotoxic dual PI3K/mTOR inhibitor had no effect on tumor growth, as assessed by neuroimaging after 14 days (only ADC values were altered) (Lee *et al*, 2015b), the results obtained by AMG386 administration are truly promising.

AMG386 sequesters both Angpt1 and Angpt2 in the TME. Although NF‐PitNET cells express much higher levels of Angpt2 than Angpt1, ECs secrete Angpt1, which can also promote tumor‐bound Tie2 activation. Moreover, AMG386 also inhibits the activation of the Tie2 receptor not only on tumor cells but also on Tie2‐ expressing ECs, pericytes, and perivascular macrophages. We believe that the efficacy of AMG386 *in vivo* indeed comes from the suppression of the exchange of these cytokines among the different cellular compartments.

Importantly, we identified elevated circulating levels of ANGPT2 in patients with NF‐PitNETs compared to age‐ and gender‐matched healthy individuals. High plasma concentration of ANGPT2 correlates with the tumors’ proliferation rate (i.e., Ki67 LI). Although some controversy remains, the Ki67 LI is considered a major indicator of clinical aggressiveness: in the latest WHO classification PitNETs are divided into “low” and “high” risk of recurrence/progression based on their proliferative activity (Ki67 LI ≥ 3%) and on p53 expression (Osamura, 2017; Inoshita & Nishioka, 2018). It has been reported that PitNETs with a K67 index ≥ 1.5% have a reduced disease‐free survival time (i.e., higher recurrence rates) (Chiloiro *et al*, 2014). Furthermore, in NF‐PitNETs, a recent study proposed the Ki67 LI as a strong prognostic factor for recurrence/progression (Yao *et al*, 2017). Increased levels of plasma/serum ANGPT2 have been reported in patients with various solid cancers, where they correlate with advanced tumor stage, recurrence, and presence of metastases (Sfiligoi *et al*, 2003; Takanami, 2004; Helfrich *et al*, 2009; Detjen *et al*, 2010; Goede *et al*, 2010; Hacker *et al*, 2016; Dong *et al*, 2018). Elevated plasma ANGPT2 concentration is therefore a putative indicator of more aggressive tumor behavior in PitNETs.

Our work provides experimental evidence that NF‐PitNET cells possess a functional Tie2 receptor and repurpose angiopoietin/Tie2 signaling typical of ECs to promote their own proliferation/survival, thereby furthering our understanding of tumor/EC crosstalk in the TME. Our results underpin the use of anti‐angiopoietin biologicals or Tie2 inhibitors not only in NF‐PitNETs but in general in other tumors found to express Tie2, thereby widening the therapeutic relevance of targeting the angiopoietin/Tie2 axis.

## Materials and Methods

### Human tissue and plasma samples

NF‐PitNETs were obtained from patients operated at the Imperial College or at the University of Tübingen (Appendix Tables S1 and S2). The Parkinson's UK Brain Bank provided post‐mortem unaffected pituitary samples. NF‐PitNETs were classified according to Knosp Grading (Micko *et al*, 2015). Plasma samples of 69 NF‐PitNET patients (38 males; 31 females; mean age: 58.2) and 69 age‐ and gender‐matched healthy volunteers (38 males; 31 females; mean age: 58) came from the Technical University Dresden and the University of Tübingen, respectively (Appendix Table S1).

Studies using human tissues were approved by the local ethics committee (Univ. Tübingen: 456/2009BO2; Univ. Munich, TUM: 169/17S) and prior to surgery, all patients signed a written informed consent, and the experiments conformed to the principles set out in the WMA Declaration of Helsinki and the Department of Health and Human Services Belmont Report.

### ANGPT2 ELISA

ANGPT2 concentration in human plasma and supernatant of human cells was measured using a validated ELISA assay according to manufacturer’s instructions (DANGPT20; R&D Systems). Assays were run on the same day, on plates from the same batch, and the same control plasma samples (*n* = 4) were included in each plate for reproducibility. Using unpaired *t*‐test with Welch’s correction: mean ± SEM healthy controls (*n* = 69) = 0.89 ± 0.058; mean ± SEM PitNET patients (*n* = 69) = 1.78 ± 0.14; Difference between means = 0.92 ± 0.15; 95% confidence interval = 0.62 to 1.21; *P* value < 0.0001.

Angpt2 levels in the supernatant of GH3 cells and derivative clones, as well as of rat primary cells was assessed with a rat/mouse ELISA assay as above (MANG20; R&D Systems). To each measurement, the value of the serum‐free medium alone (blank) was subtracted. The values of the supernatants of sh*Angpt2*‐infected cells were close to/lower than those of the blank, indicating no secretion of Angpt2.

### Zebrafish care and xenograft procedure

Fish of the strain *Tg(fli1a:EGFP)y1* were raised and maintained according to the National (Italian D.lgs 26/2014) and European (2010/63/EU and 2012/707/EU) animal guidelines. Embryos were collected by natural spawning and staged according to Kimmel and colleagues (Kimmel *et al*, 1995). GH3 cells, infected with unspecific shRNA (shCtrl) or with shAngpt2 were labeled with a viable red fluorescent dye (CellTrack‐erTM CM‐DiI, Invitrogen), resuspended in PBS and implanted into the sub‐peridermal space of embryos at 48 h postfertilization. At 24 and 48 h post‐injection, embryos were imaged through a fluorescence stereomicroscope (Leica DM6000B equipped with LAS Leica imaging software). In some pictures, the red channel was omitted to highlight the differences of tumor‐induced microvascular networks between the two experimental groups. Digital magnifications of graft regions are provided and all images shown are oriented so that rostral is to the left and dorsal is at the top. The same exposure time was used for all panels. At least 20 correctly grafted embryos have been included in each experimental group. As arbitrary unit (A.U.) of the tumor‐induced angiogenesis, the total cumulative length of endothelial structures sprouting from the SIV and CCV was measured in each grafted embryo by Fiji software and expressed in number of pixels.

### Rodent care and *in vivo* treatments

Animals were maintained in agreement with general husbandry rules approved by the Helmholtz Zentrum München. All experimental procedures were conducted in accordance with approved governmental guidelines (GV‐Solas; Felasa; TierschG) and *in vivo* studies were approved by the government of Upper Bavaria (rat studies: Az. 55.2.1.54‐2532‐39‐13 and 55.2‐2532.Vet_02‐18‐102; mouse xenografts: Az. 55.2.1.54‐2532‐111‐2015). Female MENX rats at 7‐8 months of age were injected i.p. with AMG386 (2 mg/kg body weight) or PBS every day for 3 days (*n* = 3 each group; short treatment) or 3×/week for 2 weeks (*n* = 10 each group; long treatment). Tumor monitoring in rats was conducted using magnetic resonance imaging (MRI), and diffusion weighted (DW)‐MRI with ADC maps pre‐ (day 0) and post‐treatment (day 14) was conducted on a subset of rats. At the end of each treatment, complete necropsy was conducted and the pituitary glands were fixed in 4% buffered formalin and embedded in paraffin for histological/immunohistological analyses, or stored at −80°C.

Female CD1 foxn1 nu/nu mice aged 6 weeks and weighing 27–35 g were purchased from Charles River and housed in a pathogen‐free environment at a temperature of 25°C and relative air humidity following the between 45 and 50%. Mice were injected subcutaneously in the dorsal flank region with 5X10^6^ GH3 parental (*n* = 12), KO Ctrl (*n* = 8), or Tie‐2‐knockout (*n* = 8) cells. Twenty days after injection, mice were randomized and treated with AMG386 or placebo (i.p. injection) 3×/week for 3 weeks, or monitored for tumor growth. No body weight loss was observed in upon treatment with AMG386. Tumor volume was assessed non‐invasively at day 0 (= day 20 after injection), 10, 21 post‐treatment using magnetic resonance imaging (MRI) and external caliper.

### Magnetic resonance imaging

Magnetic resonance imaging was performed on a 7T preclinical scanner (Bruker BioSpin MRI GmbH) using a ^13^C/^1^H volume resonator, a 4 channel ^1^H rat head surface coil array (for rats) and a 2 channel ^1^H surface coil array (for mice; all: RAPID Biomedical). Anesthetized animals (2.5% isoflurane, administered in pure oxygen) were placed on an animal bed in a prone position (tail ahead) and the particular surface coils were positioned over the tumor. For tumor volume determination in both rats and mice T_2_ weighted imaging was applied (sequence parameters [T2_TurboRARE]: TE: 40 ms, TR: 3,000 ms, averages: 2, Rare factor: 8, slices: 20, slice orientation: sagittal, read orientation: rostral‐caudal, slice thickness: 0.800 mm, image size [matrix]: 256 × 256, field of view [FoV]: 40 × 35 [mm²], resolution: 0.156 × 0.137 [mm²], fat suppression: on). The actual volume determination was performed in Osirix/Horos (Pixmeo SARL, Horos Project) to quantify and evaluate tumor growth. Therefore regions of interest (ROIs) were manually segmented around the PitNETs/xenografts in every slice where they appeared in the T_2_ weighted datasets; tumor volumes were finally calculated from the data (area of particular ROIs and slice thickness) using an implemented algorithm in Osirix/Horos.

Following T_2_ weighted imaging of the rat PitNETs, diffusion‐weighted MRI (DW‐MRI) was performed to quantify tumor cellularity and to monitor processes of necrosis and apoptosis (sequence parameters [Dti_EPI]: TE: 20 ms, TR: 3,000 ms, averages: 16, slices: 20, slice orientation: sagittal, read orientation: rostral‐caudal, slice thickness: 0.800 mm, image size [matrix]: 128 × 128, field of view [FoV]: 40 × 35 [mm²], resolution: 0.313 × 0.273 [mm²], fat suppression: on, b‐values: 9 (13; 49; 89; 129; 209; 309; 509; 759; 1,009 [s/mm²]). Apparent diffusion coefficient (ADC) maps were calculated according to the pixelwise fitting of the Stejskal‐Tanner equation: *S* = *S*_0_*e ^(−^ *^b^* *ADC) (with *b* being the diffusion weighting factor, *S*_0_ the signal intensity without applying any diffusion weighting and *S* the signal intensity at a certain *b* value) (Hundshammer *et al*, 2018). The data fitting was performed using the method of least squares (MATLAB, MathWorks). ROIs were drawn around the PitNETs in the four innermost ADC maps which resulted in a mean ADC for every slice and then a weighted mean ADC over the four slices was calculated in consideration of different ROI sizes. A statistically significant increase in mean ADC was found in AMG386‐treated tumors (mean ADC pre = 0.959 versus mean ADC post = 1.158; *P* = 0.009), while no significant changes were observed in placebo‐treated rats (mean ADC pre = 1.027 versus mean ADC post = 1.114; *P* = 0.177).

### Reagents, siRNA oligos, and vectors

AMG386 was supplied by AMGEN in a DMSO‐containing solution suitable for *in vitro* and *in vivo* applications. The Tie2 kinase inhibitor was purchased by Abcam (ab141270) and dissolved in DMSO. rhANGPT2 (623‐AN‐025) was from R&D Systems. Lentiviral vectors for the knockdown of Ang‐2 purchased from Sigma‐Aldrich (MISSION^®^ Lentiviral Transduction Particles) contained each a different shRNA sequence: #1: GATTTTAGCACAAAGGATT; #2: TGCAGCTTCTTCAACATTCTA; #3: ACGGCTGTGATGATCGAGATT. Control vectors contained a non‐specific shRNA sequence (MISSION pLKO.1‐puro Non‐Mammalian shRNA Control). A pool of 30 different siRNA sequences against rat Ang‐2 were purchased from siTOOLs Biotech (NM_134454). Their sequences are reported in Appendix Table S3. A different siAng‐2 oligo (Accell rat Ang‐2 siRNA: A‐089390‐16‐0010: TGCAGCTTCTTCAACATTCTA) and control scrambled siRNA were purchased from Dharmacon (D‐001810‐03‐05).

### Cell culture and treatments

The GH3 and AtT20 cell lines were purchased from ATCC. αT3 and LβT2 cells were kindly provided by Pamela Mellon (University of San Diego, CA, USA). GH3 cells were maintained in F‐12K medium with 15% horse serum (HS), 2.5% FBS, penicillin (100 IU/ml) and streptomycin (100 mg/l). αT3, LβT2 and AtT‐20 cells were grown in DMEM supplemented with high glucose (4,500 mg/l) containing 10% (v/v) FBS, penicillin (100 IU/ml) and streptomycin (100 mg/l) (Sigma‐Aldrich). Cells were routinely tested for mycoplasma and maintained in culture for maximum 5–6 passages. A dose‐response experiment was performed to identify the best concentration of AMG386 and Tie2‐KI (IC50 = 5μg/ml and 5 μM, respectively) to use for GH3 cells.

HUVECs were purchased from Sigma and cultivated using the Ready‐to‐use kit including Basal Medium and SupplementMix (C‐22010). Rat aortic endothelial cells (RAOECs) were also from Sigma and were maintained in Rat Endothelial Cell Basal Medium (R210‐500) with Rat Endothelial Cell Growth Supplement (R211‐GS). Cell culture plates and dishes were coated using Attachment Factor Solution (Sigma #123‐100).

Primary pituitary cell cultures were established from fresh rat or human tumor tissues as previously described (Lee *et al*, 2015b). For drug treatments, primary cells were plated in 96‐well plates and treated for 48 h. Cell viability was assessed, and the average of the treated wells (*n* = 6) for each individual sample was normalized against the average of the untreated wells (*n* = 6) for each sample, arbitrarily set to 1. A significant reduction in the viability of the human primary tumor cells (versus untreated controls) was observed starting from the value of −20% viability, which was then used as threshold to consider the samples responders/non‐responders.

For the separation of ECs from fresh pituitary adenoma tissues, we used a protocol reported in Sievert *et al* (2014).

### Transfections and infections

GH3 cells were transfected with 400 pmol of scrambled or siRNA against rat Ang‐2 by Amaxa 4D‐Nucleofector (Lonza). To generate ANG‐2 overexpressing GH3 line (C9OE), 1 µg plasmid DNA carrying a Myc‐tagged human ANG‐2 cDNA was used. The selection was done using Neomycin (300 µg/ml; Sigma‐Aldrich). Lentiviral infection was performed following the manufacturer’s instruction. Puromycin (4 µg/ml; Sigma‐Aldrich) was used to select infected cells.

### CRISPR‐Cas9 knockout of Tie‐2

The knockout of the *Tie‐2* gene in GH3 cells was obtained by using the Edit‐R All‐in‐one lentiviral (knockout) rat TEK sgRNA kit (Dharmacon). After infection, single cell clones were selected with puromycin, expanded and sequenced to verify the knockout of the *Tie‐2* gene. Two clones, #18 and #19, having gene deletion showed no Tie‐2 protein expression nor activation by rhANG‐2. Clone 19 was used for *in vivo* xenograft studies.

### Cell proliferation and apoptosis assays

Primary tumor cells or GH3 cells were plated in 96‐well plates (10^4^cells/well). Cell proliferation/viability was measured using WST‐1 colorimetric assay (Roche) 48 h after treatment or 24 h, 48 h, 72 h after transfection according to the manufacturer’s recommendations.

Apoptosis assay was performed using Caspase‐Glo^®^ 3/7 Assay kit (Promega) to assess the activity of caspase‐3/7 48 h after treatment or 24 h, 48 h, 72 h after transfection. Absorbance and luminescence was measured using Thermo Scientific Varioscan LUX device.

### Cell treatment with rhANG‐2 or conditioned medium

Parental or *Ang‐2*‐silenced GH3 cells were grown in serum‐free condition for 24 h then stimulated with different amounts of rhANG‐2 and various time points indicated in each experiment. For RAOECs and rat primary tumor and ECs, treatment with rhANGPT2 (800 mg/ml) or rhANGPT1 (600 mg/ml) was performed for 15 min prior to cell collection for protein extraction. CM obtained from rat primary cells was used to stimulate GH3 cells. The amounts of secreted Ang‐2 in concentrated medium were measured by ELISA and 10 µl of it was added to the cells directly or pre‐incubated with AMG386 (5 µg/ml) for 1h and subsequently added to the cells in a final concentration of 0.335 ng/ml. Cell proliferation or apoptosis were assessed 24h later. If receptor activation was studied, CM was given for 30min to the cells.

### Cell staining for flow cytometry

Following antibodies were used for flow cytometry: anti‐Tie2, PE‐conjugated (Bioss); anti‐CD31, APC‐conjugated (Miltenyi Biotec). Flow cytometric staining was performed for 30 min on ice in the dark. Cells were passed through a 40 µm cell strainer (NeoLab) to remove large debris.

Cells were acquired on a BD FACSAriaIII flow cytometer using FACSDiva software V6.1.3 with optimal compensation and gain settings based on unstained and single‐color stained beads. Doublets were excluded based on SSC‐A vs. SSC‐W and FSC‐A vs. FSC‐W plots. Live cell populations were gated on the basis of cell side and forward scatter and the exclusion of cells positive for Sytox Blue (Life Technologies) or Fixable Viability Dye eFluor506 (Invitrogen). Samples were analyzed using FlowJo software v10.8.0 (TreeStar Inc., OR). CD31^+^Tie2^+^ and CD31^−^Tie2^+^ cells were sorted with a BD FACS Aria III cell sorter for purity. Per sample between 9,000–43,000 (CD31^+^Tie2^+^) cells and 172,000–320,000 (CD31^−^Tie2^+^) cells were sorted and used for qRT–PCR.

### RNA extraction and quantitative TaqMan RT–PCR

RNA was extracted using RNeasy Mini Kit (Qiagen) following the manufacturer’s instructions. Quantitative RT–PCR was performed using TaqMan inventoried primers and probes (Applied Biosystem) for the corresponding genes as previously reported (Lee *et al*, 2015b).

### Protein extraction, western blotting, and immunoprecipitation (IP)

Cells (GH3 or primary cells) were collected, washed with PBS, and lysed in ice‐cold lysis buffer (Rippa, Sigma‐Aldrich) supplemented with protease and phosphatase. Western blotting was conducted using antibodies listed in Appendix Table S5. The membranes were incubated with HRP‐conjugated secondary antibodies (Bio GE Healthcare) for 1 h followed by washes in TBS‐T and incubation with West Pico chemiluminescent substrates (Thermo Fisher Scientific) to visualize immunoreactive proteins.

### Immunostaining and scoring

For immunohistochemistry (IHC) of formalin‐fixed, paraffin‐embedded (FFPE) tissues, 2 μm sections were cut and processed for staining with an automated immunostainer (Ventana Medical Systems, Tucson, AZ) as described previously (Lee *et al*, 2015a). Primary antibodies (Appendix Table S5) were diluted in DCS antibody diluents. Hematoxylin was used as counterstaining. Positive controls were included in each run and sections without the primary antibody served as a negative control.

IHC for ANG‐1 and ANG‐2 were scored semi‐quantitatively on acquired images. Scoring was performed independently by two investigators (FR, an experienced neuropathologist, and by NSP, with 18‐year experience in scoring histology/immunohistochemistry of neuroendocrine tumors), by a double‐blind method, with an inter‐observer variability ranging from 1 to 3.7%. The intensity of the staining and the percentage of positive cells were taken into account to obtain a final score (12): 0 (negative); + (faint); ++ (moderate); +++ (strong). Images were captured using a Hitachi camera HW/C20 installed in a Zeiss Axioplan microscope with Intellicam software (Carl Zeiss MicroImaging).

For immunofluorescence (IF) of tissues, primary antibodies (Appendix Table S5) were diluted in Dako REAL solution. The sections were incubated with fluorophore conjugated secondary antibodies (Appendix Table S5) for 1h in room temperature. Cell nuclei were counterstained with DAPI. Sections were analyzed with a Zeiss Axiovert 200 epifluorescence microscope including Apotome unit (Carl Zeiss) or Olympus CLSM FLuoView FV1200.

Quantification of IF staining intensities in cells was performed using ImageJ (NIH). Images were subjected to the threshold function, and the same threshold for all images obtained with the same antibody was used. Then, the intensity of the staining was quantified for 30 cells in total for each experimental condition and is indicated as average ± SEM for each condition.

The Ki67 labeling index (= nuclear immunoreactivity) of rat PitNETs after treatment was determined semiquantitatively by counting the positive nuclei in 3× high‐power fields (HPF; at 400×) per tissue (*n* = 3 per group) and is indicated as the percent of positive cells against all neoplastic cells in the fields examined.

For IF of cells, 8x10^5^ cells were plated onto coverslips previously coated with poly‐l‐lysine and cultured in the appropriate medium overnight. Cells were washed with PBS and fixed in freshly prepared 2% paraformaldehyde in PBS. After fixation, cells were washed with PBS, permeabilized in 0.1% Triton X‐100, washed again with PBS, and blocked in 5% normal goat serum (in PBS) for 30 min. Cells were then incubated overnight at 4°C with the primary antibody. The appropriate secondary antibody diluted in 5% normal goat serum, was added to the cells for 1h at room temperature and nuclei were stained with DAPI.

### Proximity ligation assay

One micron sections of FFPE rat pituitary tumor tissues were deparaffinized, rehydrated, and cooked in citrate buffer. After cooking, the tissue samples were washed twice in TBS‐T + 1 M Glycin and once in TBS‐T. Then the blocking solution (provided by the PLA kit) was added to each sample followed by incubation at 37°C for 30 min (Sigma‐Aldrich). After incubation the primary antibodies, anti‐Ang‐2 (AF623, R&D) and anti‐Tie‐2 (PA5‐28582, Thermo Scientific) or the combination of anti‐Ang‐2 and anti‐P‐Tie‐2 (PC449, Millipore) was added to each sample and incubated overnight at 4°C. Afterward, the tissue samples were washed twice with washing buffer A (Sigma‐Aldrich). Then, the specific proximity probes targeting goat and rabbit IgGs (secondary antibodies attached to single stranded DNA oligonucleotides) were added at a 1:5 dilution using the antibody diluents supplied by kit. After 1h incubation at 37°C in a humidity chamber, the tissue samples were washed and the ligation solution (included in the kit) was added to the samples and incubated for 30 min at 37°C in a humidity chamber. After ligation, the samples were washed again using the same washing buffer. Then the samples were treated with the amplification and detection solution (Sigma‐Aldrich) and were incubated for 100 min in a humidity chamber at 37°C. Afterward, the samples were washed in washing buffer B and for 1 min in diluted (1:100) washing buffer B in dark Sigma‐Aldrich. The samples were left to dry at room temperature and were mounted using a mounting medium containing DAPI and stored at 4°C. Optical sections of tissues used for PLA assays were taken with Olympus CLSM FLuoView FV1200. PLA signal was quantified by counting the number of cells with discrete spots in 3× HPF (at 400× magnification) per sample (*n* = 3). In total, 1,914 cells were analyzed having a total of 1,294 PLA spots. Cells having 1 discrete spot were 452, with 2 spots 167 and with ≥3 spots 41.

For PLA on cells, RAOECs or isolated primary tumor cells were plated on coated coverslips (Attachment Factor Solution for endothelial cells and collagen‐coating for primary cells). The next day, primary tumor cells were washed with PBS and either treated with rhANGPT2 (800 mg/ml) for 15 min or left untreated, fixed in freshly prepared 2% paraformaldehyde in PBS. RAOECs were also washed and fixed as above. After fixation, cells were washed with PBS and then blocked using the blocking solution provided by the PLA kit at 37°C for 30 min. Further PLA procedure was carried out as outlined above for the FFPE tissues.

### Statistical analysis

Study endpoints from *in vitro* experiments, including cell proliferation, apoptosis, migration, colony formation, as well as primary cell viability, were displayed by bar graphs with means ± SEM. Sample sizes were determined based on use of the two‐sample *t*‐test for detecting group differences at the 0.05 significance level. The sample sizes achieved power greater than or equal to 80% for detecting clinically relevant differences between groups that could be anticipated based on prior feasibility and published studies. The ethics commission reviewed and approved these sample sizes.

Unpaired Welch's *t*‐tests were used for detecting differences between series of data, separately at all time points, as well as for differences in zebrafish vessel sprouting between groups. Statistical comparisons between more than two groups were performed using the one‐way ANOVA with F‐test. For analysis of imaging scans, one‐way ANOVA was used.

All statistical analyses were executed using GraphPad (GraphPad Software Inc.), with the level of statistical significance set at 0.05.

## Author contributions

**Ninelia Minaskan Karabid:** Conceptualization; Investigation; Writing—original draft; Performed experiments. **Tobias Wiedemann:** Conceptualization; Formal analysis; Investigation. **Sebastian Gulde:** Formal analysis; Investigation; Writing—review and editing. **Hermine Mohr:** Writing—review and editing. **Renu Chandra Segaran:** Investigation. **Julia Geppert:** Investigation. **Maria Rohm:** Data curation. **Giovanni Vitale:** Formal analysis; Investigation. **Germano Gaudenzi:** Formal analysis; Investigation. **Alessandra Dicitore:** Investigation. **Donna Pauler Ankerst:** Data curation; Formal analysis; Writing—original draft; Writing—review and editing. **Yiyao Chen:** Formal analysis. **Rickmer Braren:** Formal analysis; Investigation. **Georg Kaissis:** Investigation. **Franz Schilling:** Formal analysis; Investigation. **Mathias Schillmaier:** Investigation. **Graeme Eisenhofer:** Resources; Data curation. **Stephan Herzig:** Conceptualization; Data curation. **Federico Roncaroli:** Resources; Data curation; Writing—original draft. **Jürgen B Honegger:** Resources; Investigation. **Natalia S Pellegata:** Conceptualization; Data curation; Writing—original draft; Writing—review and editing.

In addition to the CRediT author contributions listed above, the contributions in detail are:

NSP and NMK conceptualized the study and designed the experiments. NMK, HM, SG performed *in vitro* experiments; TW, SG conducted the *in vivo* studies in mice and rats; RCS performed immunostainings; JG and MR carried out and analyzed flow cytometry data; GV, GG and AD generated and analyzed zebrafish xenografts; DPA and YC provided statistical support and analysis; RB, MS, FS and GK conducted and analyzed MRI scans; GE and JBH assisted with patient recruitment and plasma sample acquisition; FR performed pathological examination; figures were selected by NMK and NSP and prepared by NMK, TW, GG, YC and GK. NMK, DPA, JBH and NSP wrote the manuscript. TW, GV, GE and SH critically reviewed and edited the manuscript.

## Disclosure and competing interests statement

The authors have no conflicts of interest to declare.

## Supporting information



Appendix

Expanded View Figures PDF

Source Data for Expanded View/Appendix

Source Data for Figure 2

Source Data for Figure 3

Source Data for Figure 4

Source Data for Figure 5

## Data Availability

The study generated no data that required deposition.
