# Supplementary material for: Angpt2/Tie2 autostimulatory loop controls tumorigenesis
Source: EMBO Mol Med. 2022 Mar 10;14(5):e14364. doi: 10.15252/emmm.202114364 (PMC9081903; doi:10.15252/emmm.202114364)
Supplement: Supplementary file 1 — Appendix [file EMMM-14-e14364-s007.pdf]

EMM-2021-14364

APPENDIX

Table of content

File name	Page
Appendix Figure S1 + figure legend	2
Appendix Figure S2 + figure legend	3
Appendix Figure S3 + figure legend	4
Appendix Figure S4 + figure legend	5
Appendix Figure S5 + figure legend	6
Appendix Figure S6 + figure legend	7
Appendix Figure S7 + figure legend	8
Appendix Figure S8 + figure legend	9
Appendix Figure S9 + figure legend	10
Appendix Figure S10 + figure legend	11
Appendix Figure S11 + figure legend	12
Appendix Figure S12 + figure legend	13
Appendix Figure S13 + figure legend	14
Appendix Figure S14 + figure legend	15
Appendix Figure S15 + figure legend	16
Appendix Figure S16 + figure legend	17
Appendix Figure S17 + figure legend	18
Appendix Table S1	19
Appendix Table S2	20
Appendix Table S3	21
Appendix Table S4	22
Appendix Table S5	23

Appendix Figure S1

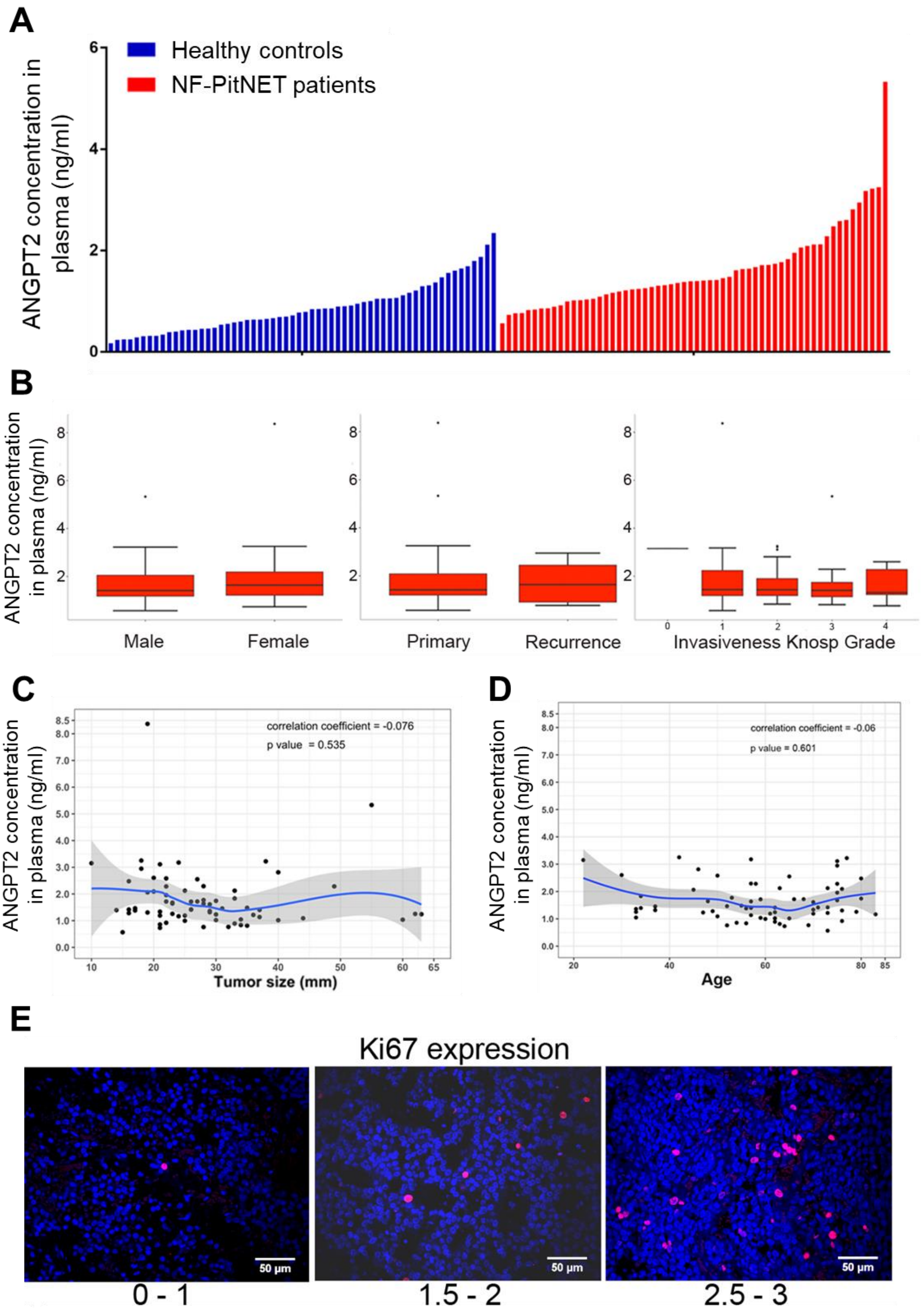

Appendix Figure S1. Correlation between circulating ANGPT2 levels in NF-PitNET patients and clinical parameters. (**A**) Concentration of ANGPT2 in plasma samples from control individuals (n=69) and NF-PitNET patients (n=69) determined by ELISA. Each bar represents the average value of each sample measured in duplicate. (**B**) ANGPT2 plasma concentration was correlated with patients' gender, primary/recurrence status and the Knosp grade of tumors. Differences between sample groups were not statistically significant. (**C, D**) ANGPT2 plasma concentration (ng/ml) was correlated with tumor size (mm) (**C**) or age (**D**) for the 69 patients. The corresponding correlation coefficients are given, which are not significant (P=0.535 or P=0.601, respectively, by t-test). The blue line is based on the locally estimated scatterplot and in grey are the 95% confidence intervals. (**E**) Immunofluorescence for Ki67 of exemplary PitNET samples belonging to the Ki67 LI groups indicated in main Fig 1B. Original magnification: 400x; Size bar: 50 µm.

Appendix Figure S2

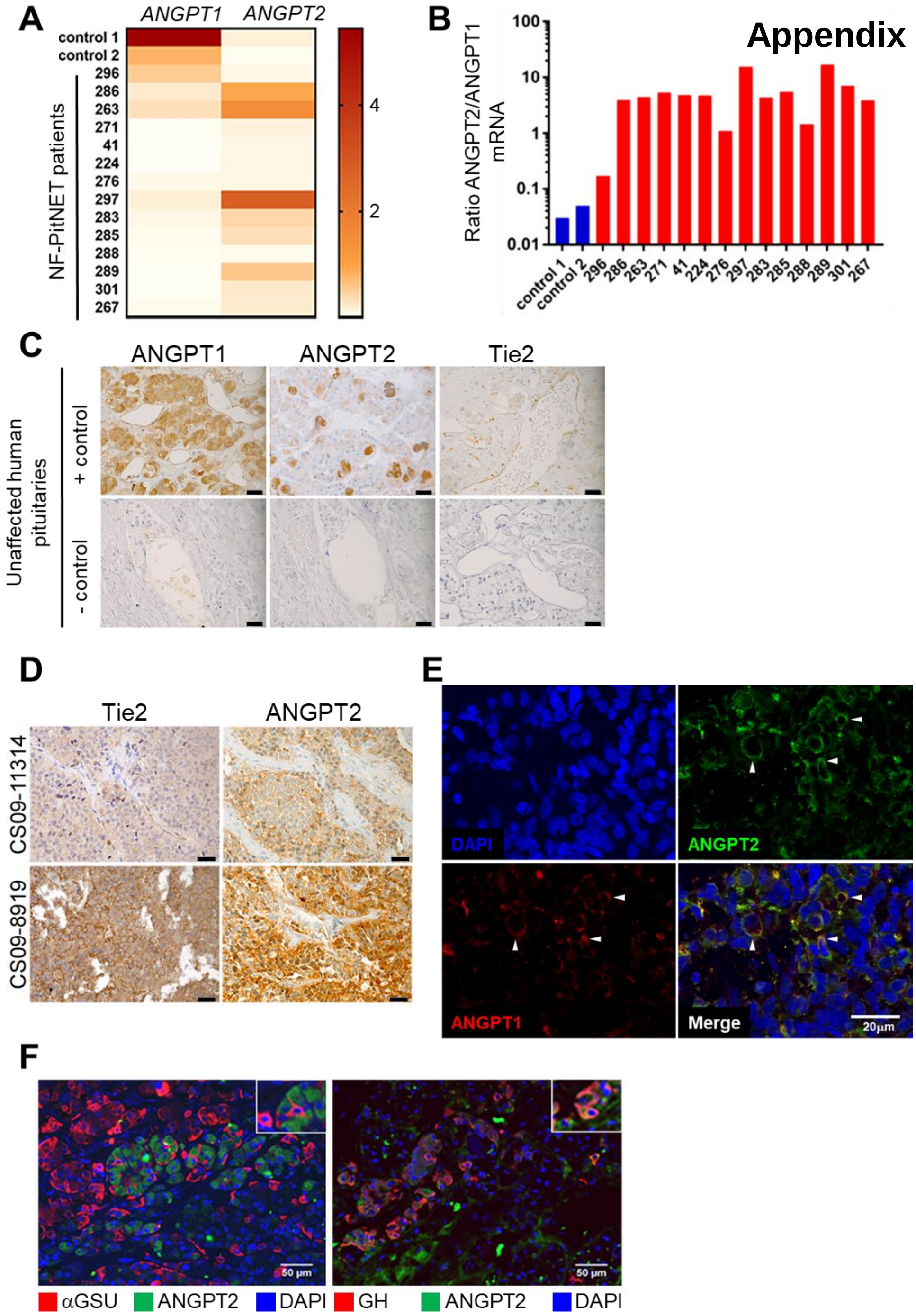

Appendix Figure S2. Expression of Angpt2 and Tie2 in human PitNETs. (**A**) qRT-PCR for *ANGPT1* and *ANGPT2* on pituitary samples from two commercially available normal human pituitary RNAs and from 14 NF-PitNET patients (Appendix Table S2). The relative mRNA expression level of the target genes was normalized for input RNA using TBP as housekeeping gene and calculated with the $2^{-\Delta\Delta Ct}$ formula. A calibrator human adrenal RNA was run in parallel. Here a heatmap is shown where the intensity of the color correlates with the level of expression of the genes shown in log scale on the right. (**B**) Shown is the ratio *ANGPT2/ANGPT1* as calculated based on the data in panel **A**. (**C**) Immunohistochemical staining (IHC) was performed on pituitary FFPE tissues from control human healthy pituitaries using antibodies against Angpt1, Angpt2 and Tie2. Negative controls („-“) were obtained omitting the primary antibody. Original magnification: 400x; scale bar: 20 µm. (**D**) IHC was performed on two representative human NF-PitNETs (out of 10) for the expression of Tie2 and ANGPT2. The tumor area is shown. ANGPT2 staining of sample CS09-11314 is also reported in Fig 1C. Original magnification: 400x; scale bar: 20 µm. (**E**) Co-immunofluorescence (IF) for ANGPT1 and ANGPT2 of human NF-PitNETs (n=4). White arrows point to double-positive tumor cells. Original magnification: 400x; scale bar: 20 µm. (**F**) Co-immunofluorescence was performed on normal human pituitary tissue using antibodies against ANGPT2 and α-subunit (α-GSU), or ANGPT2 and growth hormone (GH). Original magnification: 200x; scale bar: 50 µm.

Appendix Figure S3

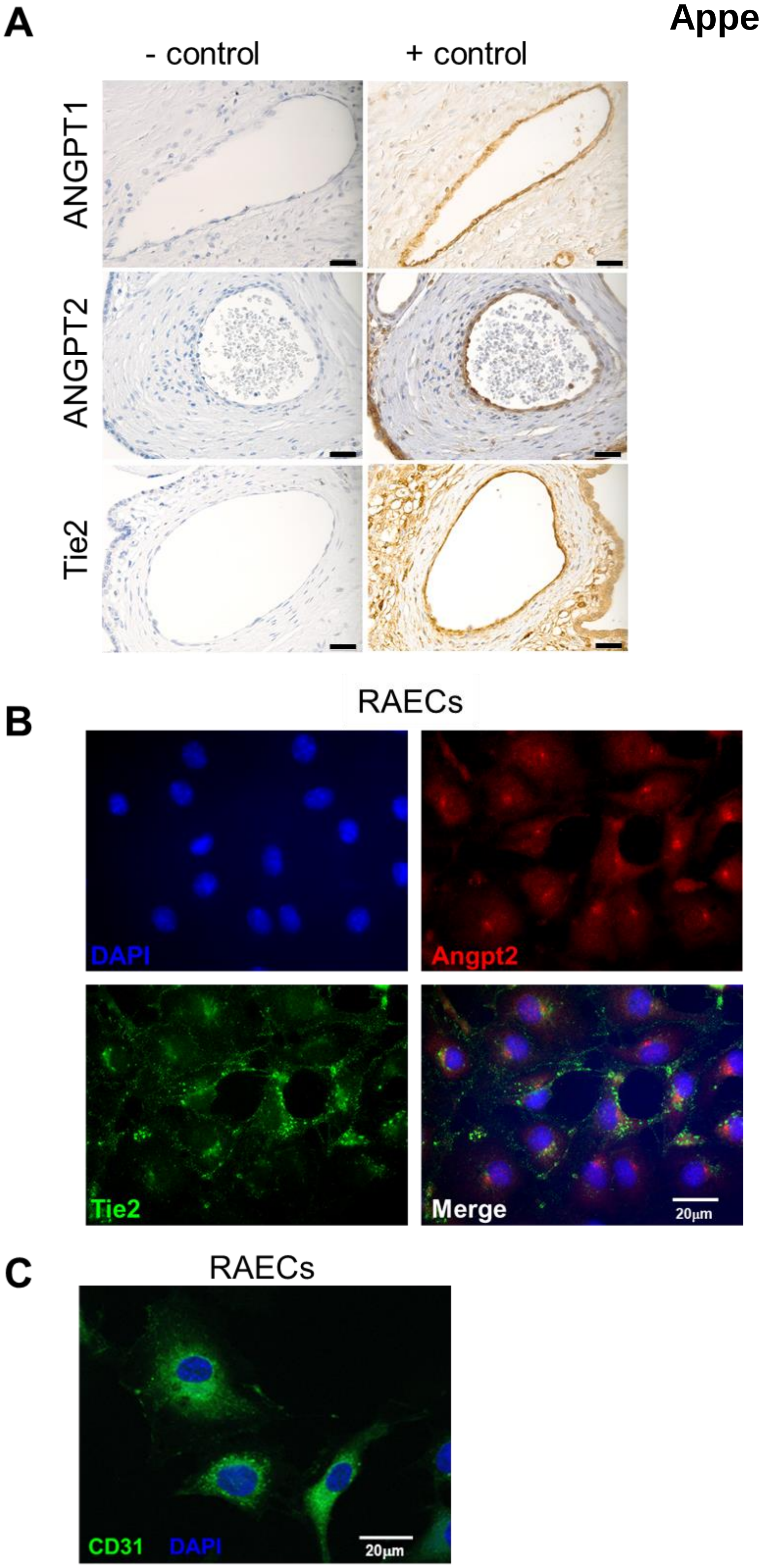

Appendix Figure S3. Expression of Angpt1, Angpt2 and Tie2 in human PitNETs and control ECs. (**A**) IHC of human prostate (ANGPT1) or placenta (ANGPT2, Tie2) for the indicated antibodies. These tissues were used as positive control tissues for the 3 antibodies („+“). Negative controls („-“) were obtained omitting the primary antibody. (**B**) Co-IF of rat aortic ECs (RAOECs, positive control cells) for Angpt2 (red) and Tie2 (green) to further validate the antibodies. Nuclei were counterstained with DAPI (blue). Original magnification: 400x; scale bar: 20 µm. (**C**) IF of RAOECs, positive control cells, for CD31(green) to validate the antibody. Nuclei were counterstained with DAPI (blue). Original magnifcation: 630x; scale bar: 20 µm.

Appendix Figure S4

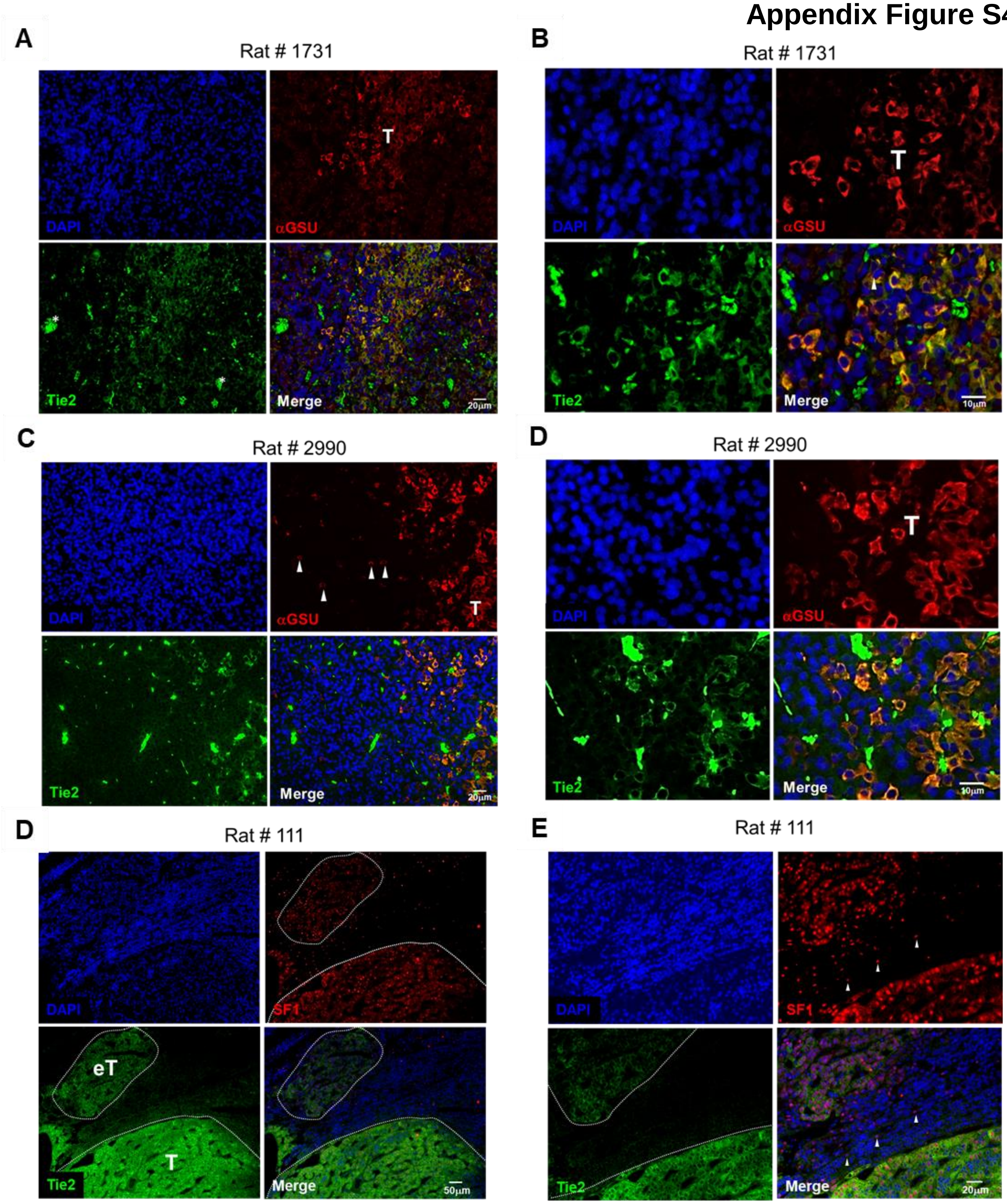

Appendix Figure S4. Expression of Tie2 and markers of gonadotroph cells in rat primary PitNETs. (**A-C**) Pituitaries from tumor-bearing MENX rats (n=3) were stained with antibodies against aGSU (red) and Tie2 (green). Two representative cases are show. Nuclei were counterstained with DAPI. The tumor areas are indicated (T). White arrows in **C** point to aGSU-positive gonadotroph cells in the adjacent nontumor area, which are negative for Tie2. Original magnifications: **A,B**: 630x; scale bar: 10 µm; **c**: 400x; scale bar: 20 µm. (**D,E**) Pituitaries from tumor-bearing MENX rats (n=3) were stained with antibodies against the steroidogenic factor (SF1) transcription factor (red) and Tie2 (green). One representative case is shown. Tumor areas are indicated. Nuclei were counterstained with DAPI. White arrows in **D** point to SF1-positive gonadotroph cells in the adjacent nontumor area, which are negative for Tie2. T, tumor area; eT, early tumor. Original magnifications: **D**: 200x; scale bar: 50 µm; **E**: 400x; scale bar: 20 µm.

Appendix Figure S5

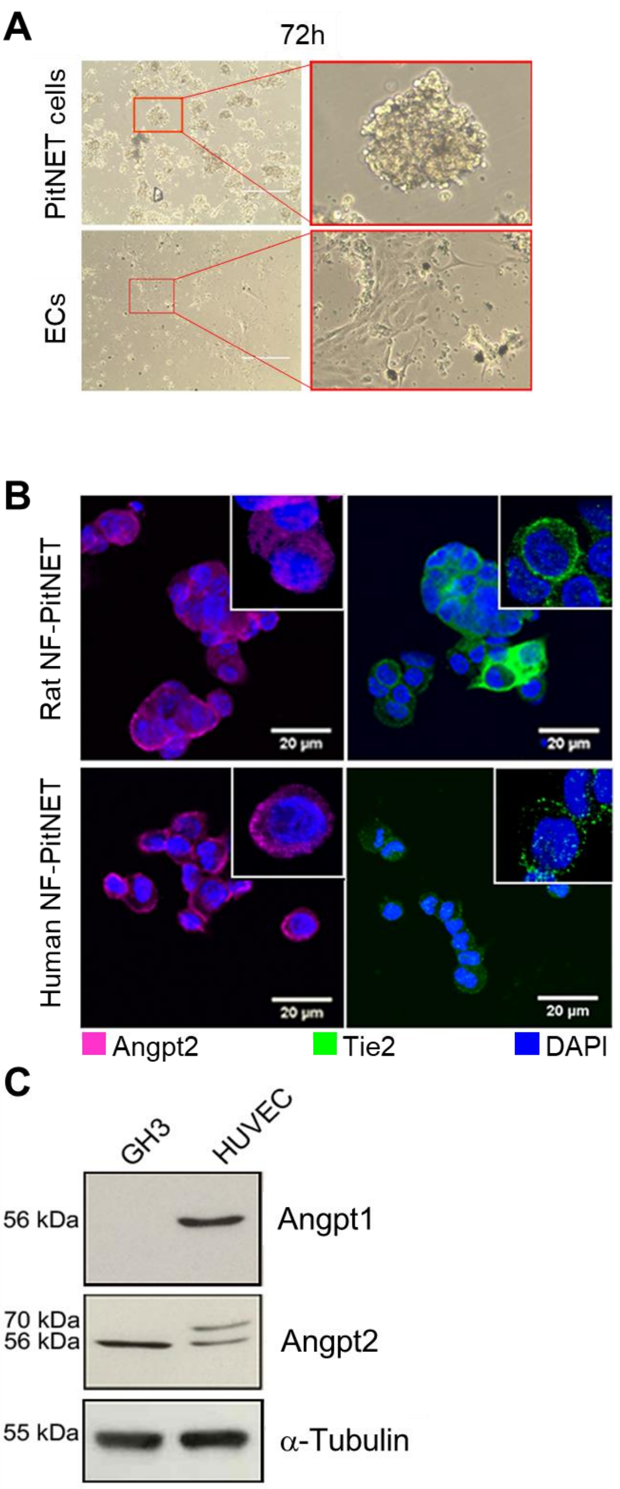

Appendix Figure S5. Separation of tumor from ECs in primary cell suspensions, expression of angiopoietins/Tie2 in primary NF-PitNET cells, in GH3 cells, in HUVECs.

(**A**) Primary PitNETs were processed to obtain single cell suspensions. ECs were separated from PitNET cells using biotin-conjugated anti-CD31 antibody and streptavidin coated magnetic beads. Both cell populations were maintained in culture as described in Materials and Methods and photos of representative cultures were taken 72h later. Original magnification: 200x (left panels); scale bar: 400 µm. Red panels on the right are digitally enlarged images of the red squares in the left panels. (**B**) Immunofluorescence of rat (R-PitNET) and human (H-PitNET) primary tumors for Angpt2 (red) and Tie2 (green). Nuclei were counterstained with DAPI (blue). Original magnification: 400x; scale bar: 20 µm. (**C**) Expression of Angpt1 and Angpt2 was assessed in GH3 cells and HUVECs (positive control) by western blotting using specific antibodies. a-Tubulin was included as loading control. Blots shown are representative of 3 independent experiments with similar results.

Appendix Figure S6

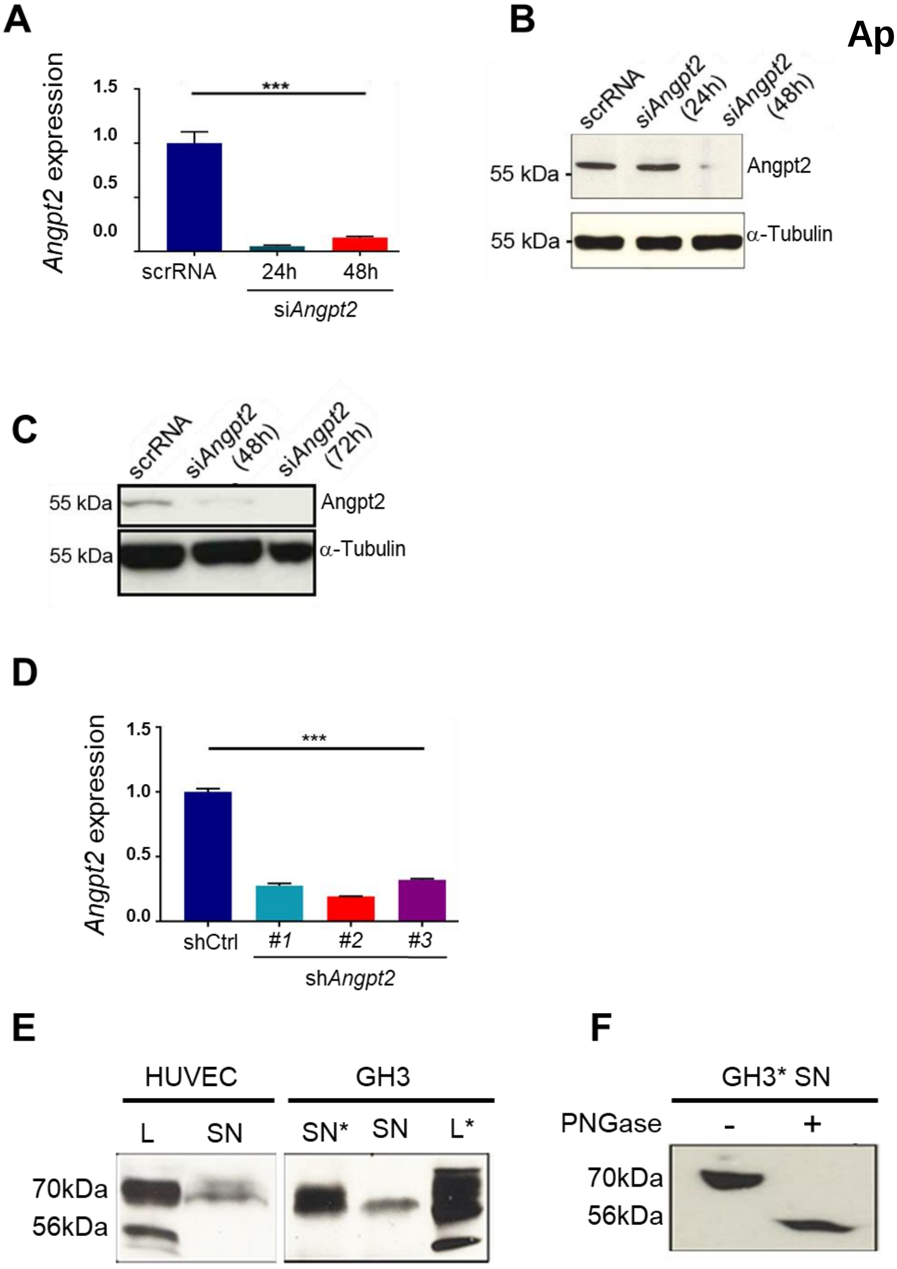

Appendix Figure S6. Individual siRNA-mediated Angpt2 downregulation in GH3 cells and expression/secretion of Angpt2. (**A**) GH3 cells were transfected with scRNA or si*Angpt2* POOLs. 24h and 48h after transfection total RNA was extracted and qRT-PCR for Angpt2 was performed. β2-microglobulin served as endogenous control for normalization of RNA input. Angpt2 levels in siAngpt2-transfected cells were calculated with the $2^{-\Delta\Delta Ct}$ formula and normalized against scRNA-transfected cells, arbitrarily set to 1. Data were analyzed independently with 2 biological and 3 technical replicates each and are expressed as the mean ±SEM. ***, P<0.0001 (one-way ANOVA). (**B**) The protein lysates of samples as in **A** were analyzed by western blot using a specific anti-Angpt2 antibody (1:200). α-Tubulin (1:1000) was used as loading control. (**C**) The protein lysates of GH3 cells transfected with the individual si*Angpt2* or with scRNA were analyzed 48h and 72h after transfection by western blot using a specific anti-Angpt2 antibody (1:200). α-Tubulin (1:1000) was used as loading control. (**D**) GH3 cells were transduced with unspecific shRNA control (shCtrl) or lentiviral vectors expressing 3 different sh*Angpt2* sequences (named #1,2,3). Two weeks after selection, RNA was extracted and qRT-PCR conducted as in indicated in Materials and Methods. Data were analyzed independently with 2 biological and 3 technical replicates. ***, P<0.0001 by t-test. (**E**) Protein lysates (L) and supernatants (SN) from HUVECs (control cells), GH parental and GH3-ANGPT2OE cells (indicated with *) were analyzed for the expression of Angpt2 by western blotting using the anti-Angpt2 antibody. (**F**) The SN of GH3-ANGPT2OE cells was treated with PNGase to remove N-linked oligosaccharides from glycosylated Angpt2 (+) or left untreated (-), and analyzed by western blotting for Angpt2. (**E-F**) Results shown are representative of 3 experiments with comparable results.

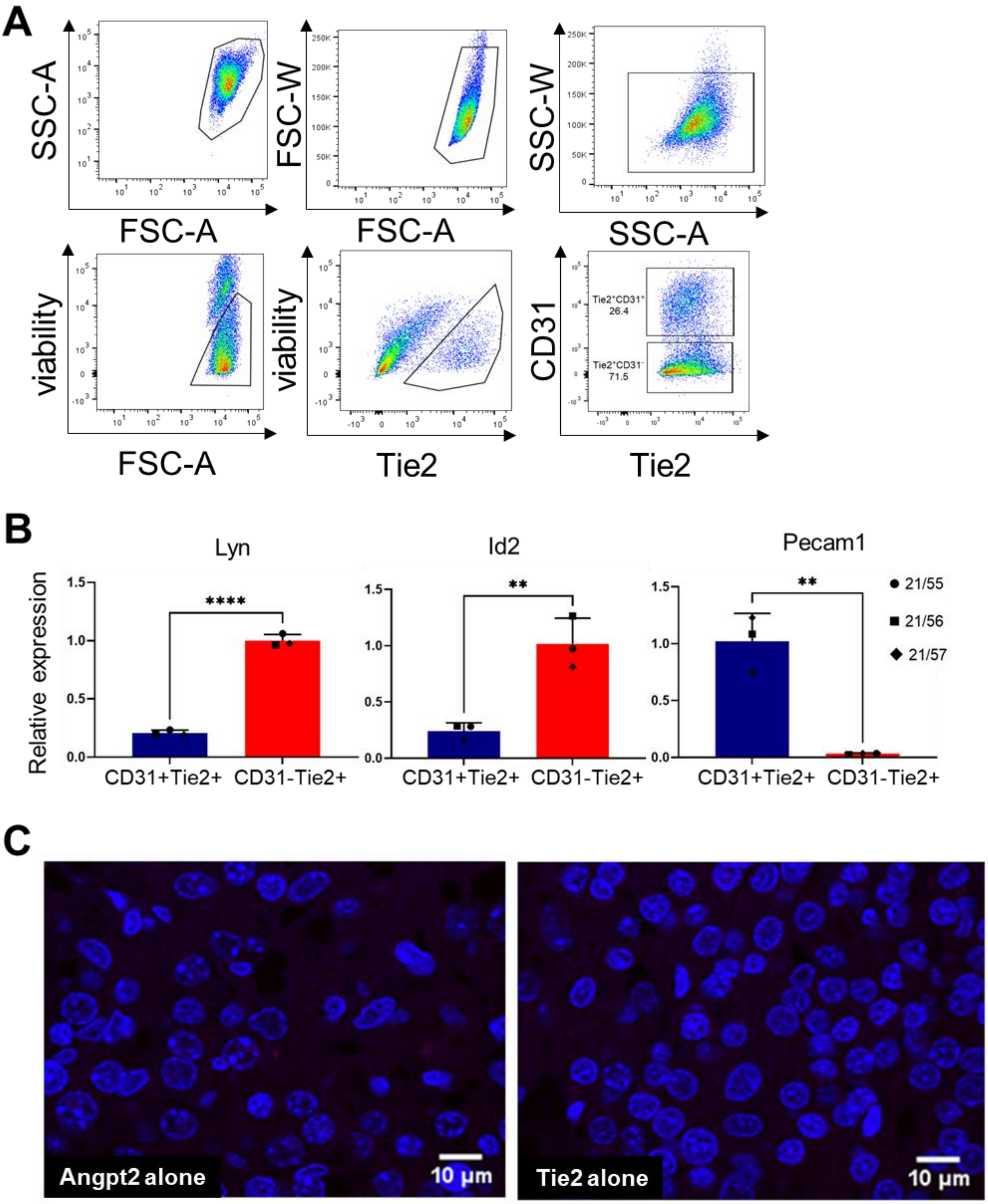

Appendix Figure S7. Flow cytometry gating and PLA on RAOECs. (**A**) Gating strategy for Tie2- and CD31-exposing cells from rat primary pituitary cultures. (**B**) qRT-PCR was performed on the cell populations indicated in **A** upon sorting. The *Pecam1* gene was used as marker of CD31+ cells, whereas *Lyn* and *Id2* have been previously found to be highly expressed in rat NF-PitNETs (Lee *et al.,* 2013). (**C**) PLA assays performed using only the Angpt2 or the Tie2 antibodies on primary rat NF-PitNET tissues as negative controls.

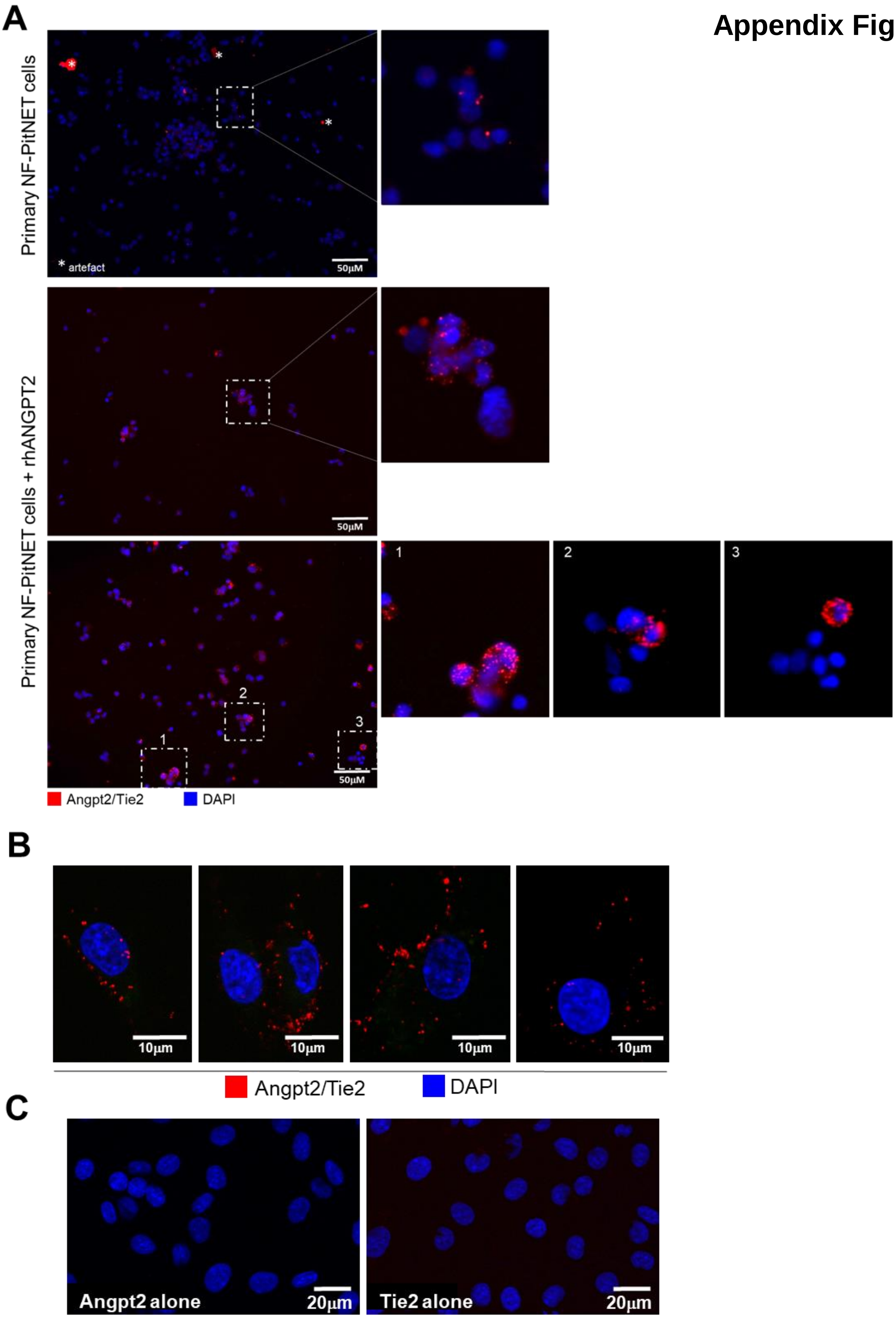

Appendix Figure S8. PLA to detect Angpt2 and Tie2 interaction in isolate rat primary NF-PitNET cells. (**A**) Isolated NF-PitNET cells from 2X9-months old MENX were plated in serum-free medium and 24h later incubated with rhANGPT2 for 15 minutes or left untreated, then fixed and processed for PLA using antibodies against Angpt2 and Tie2 (red signal). Nuclei were counterstained with DAPI. 1,2,3, different areas enlarged in the right panel. *, staining artefacts. Original magnification: X200; size bar: 50mm. (**B**) PLA was performed on cultivated RAOECs using the antibodies reported in **A**. Nuclei were counterstained with DAPI. (**C**) PLA was conducted on RAOECs using each individual antibody alone as negative control.

Appendix Figure S9

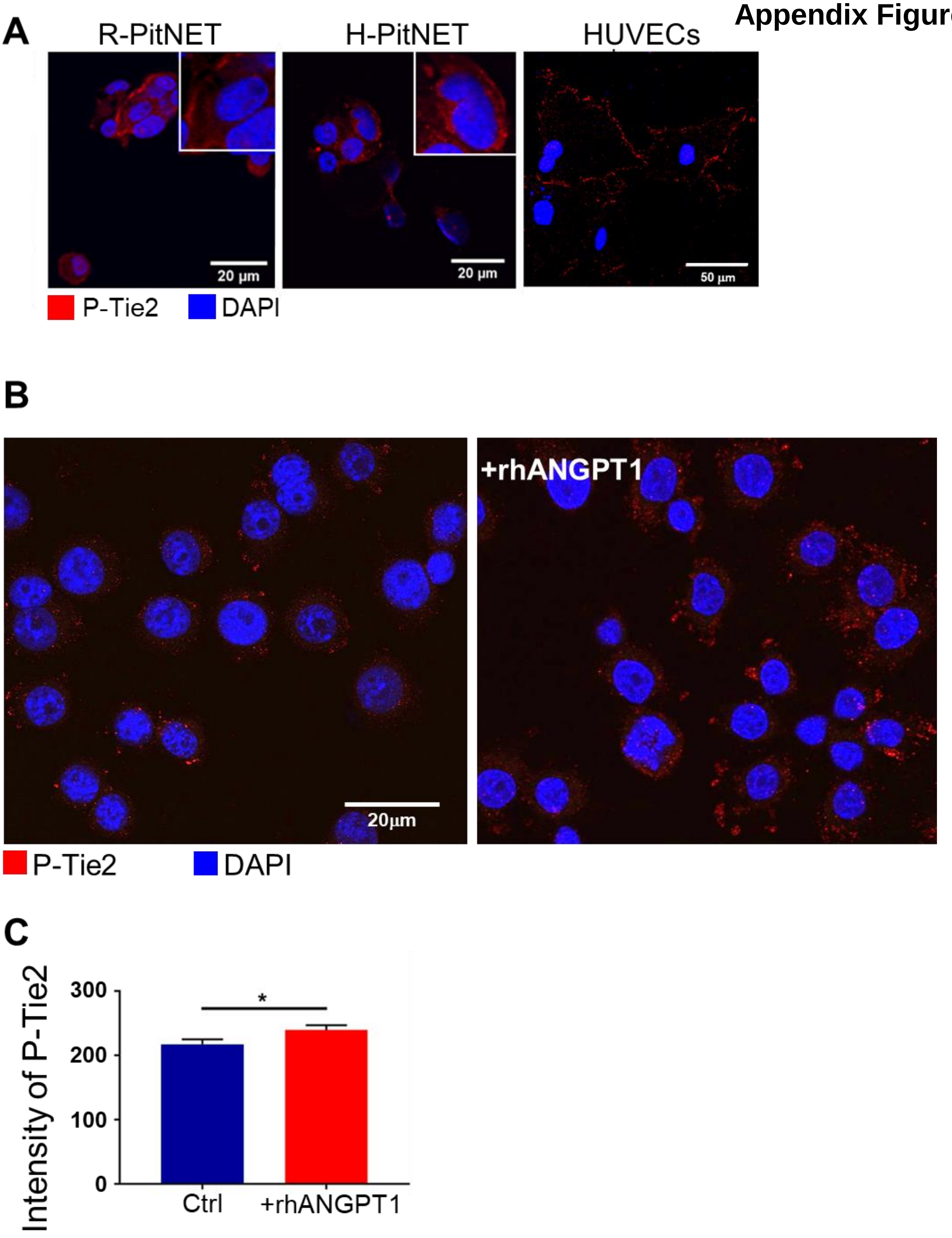

Appendix Figure S9. Basal and induced phosphorylation of Tie2 receptor in PitNET primary cells and GH3 cells. (**A**) Expression of P-Tie2 (Tyr 1102/1108; red) in primary PitNET cells and in HUVECs control cells. Nuclei were counterstained with DAPI. Original magnification: 400x; scale bar: 20 µm. (**B**) GH3 cells were grown in serum-free medium for 24h and then stimulated with 800 ng/ml rhANGPT1 for 15 min to stimulate Tie2. IF was performed for P-Tie2 (Tyr 1102/1108; red). Nuclei were counterstained with DAPI (blue). Pictures shown were taken with the same exposure time and are representative of three independent experiments. Original magnification: 400x; scale bar: 20 µm. (**C**) Quantification of staining intensity of cells treated as in panel **B**. The intensities were estimated as in Materials and Methods, and are expressed as arbitrary units ± SEM. Parental GH3 cells: 217.34 ± 7.65; GH3+rhANGPT1 800ng/ml: 239.49 ± 7.38 (vs. GH3, P=0.0446, by t-test).

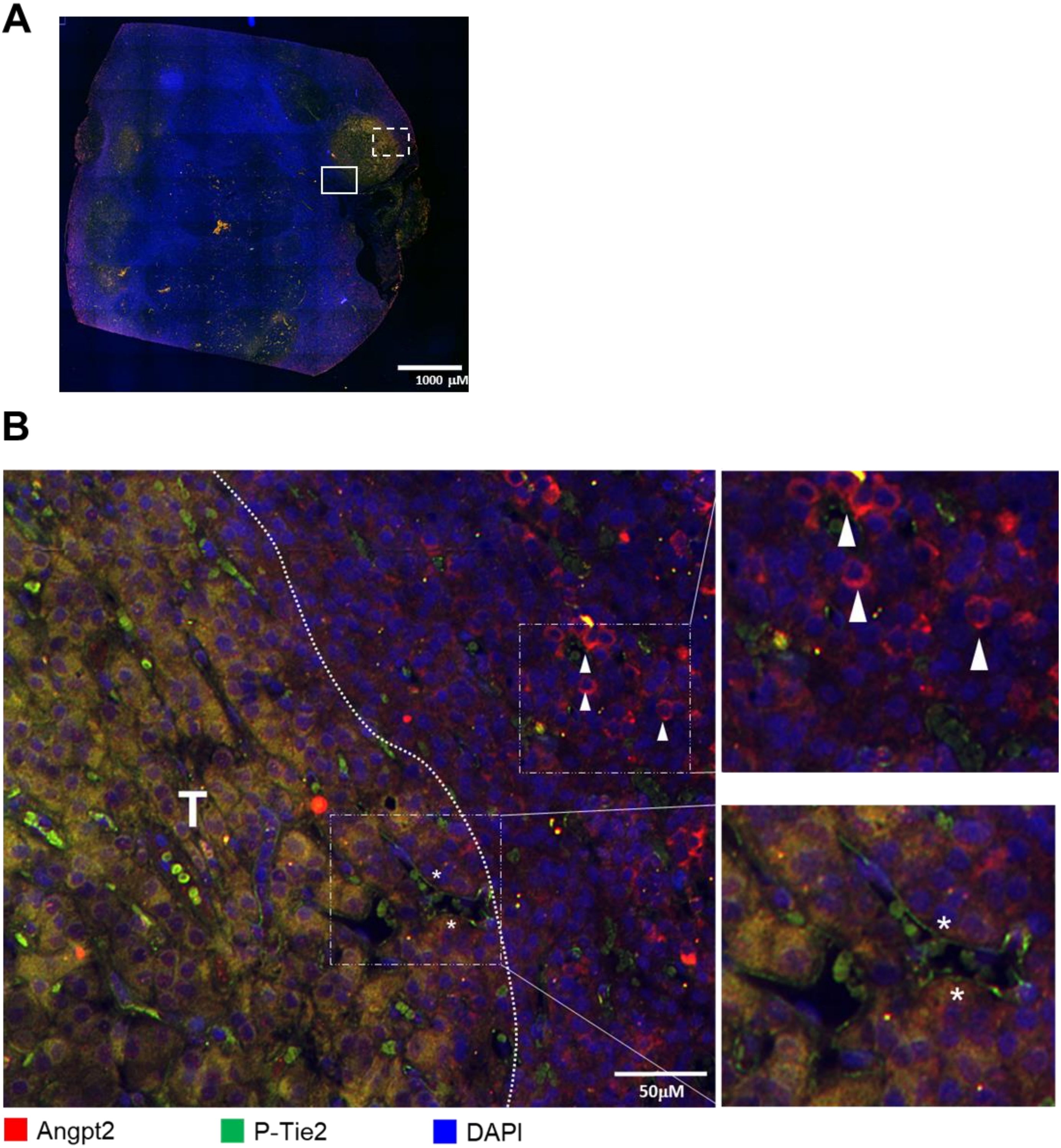

Appendix Figure S10. Expression of Angpt2 and phosphorylated Tie2 receptor in rat primary PitNETs. (**A**) Co-immunofluorescence (IF) for Angpt2 (red) and P-Tie2 (Tyr 1102/1108; green) of pituitary glands of tumor-bearing MENX rats (n=3). One representative tissue is shown. The area delineated by the white dashed line is enlarged in panel **B**. The line delineated by the white line is shown in Fig EV3. Nuclei were counterstained with DAPI. Original magnification: 40x; scale bar: 1000 µm. (**B**) The white arrows in the enlarged panel indicate P-Tie2-positive ECs, used as positive control. Original magnification: 200x; scale bar: 50 µm. T, tumor area.

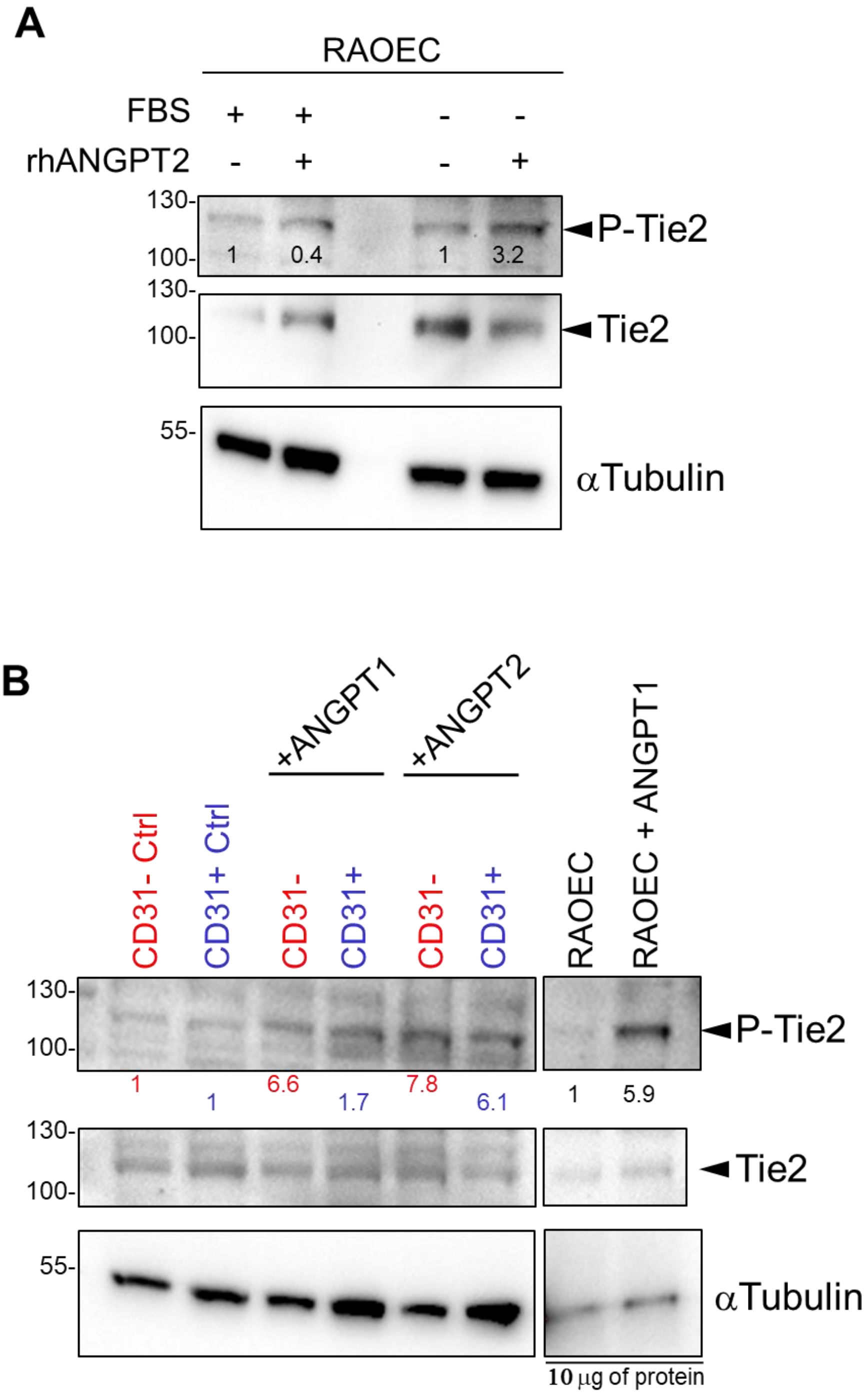

Appendix Figure S11. Expression of Tie2 and P-Tie2 in RAOECs and isolated primary rat cells. (**A**) RAOECs were grown in full medium (+FBS) or in serum-depleted medium (-FBS) for 24h and then stimulated with 800 ng/ml of rhANGPT2, or left untreated. Total proteins were extracted and WB was probed with the anti-total-Tie2 or the anti-P-Tie2 (Y992). (**B**) Isolated primary rat pituitary cells (CD31- = NF-PitNETs; CD31+ = ECs) from 2x9-month-old rat pituitaries were pooled, stimulated with the indicated recombinant proteins or left untreated. Proteins extracted and loaded onto a gel. WB was probed with the indicated antibodies. Control RAOECs cells were included as positive control. To avoid overexposure, only 10 mg of total RAOECs were loaded. The numbers represent the ratio P-Tie2/Tie2. The numbers represent the ratio phospho/total proteins. α-Tubulin (1:1000) was used as loading control. Blots shown are representative of 3 independent experiments with similar results.

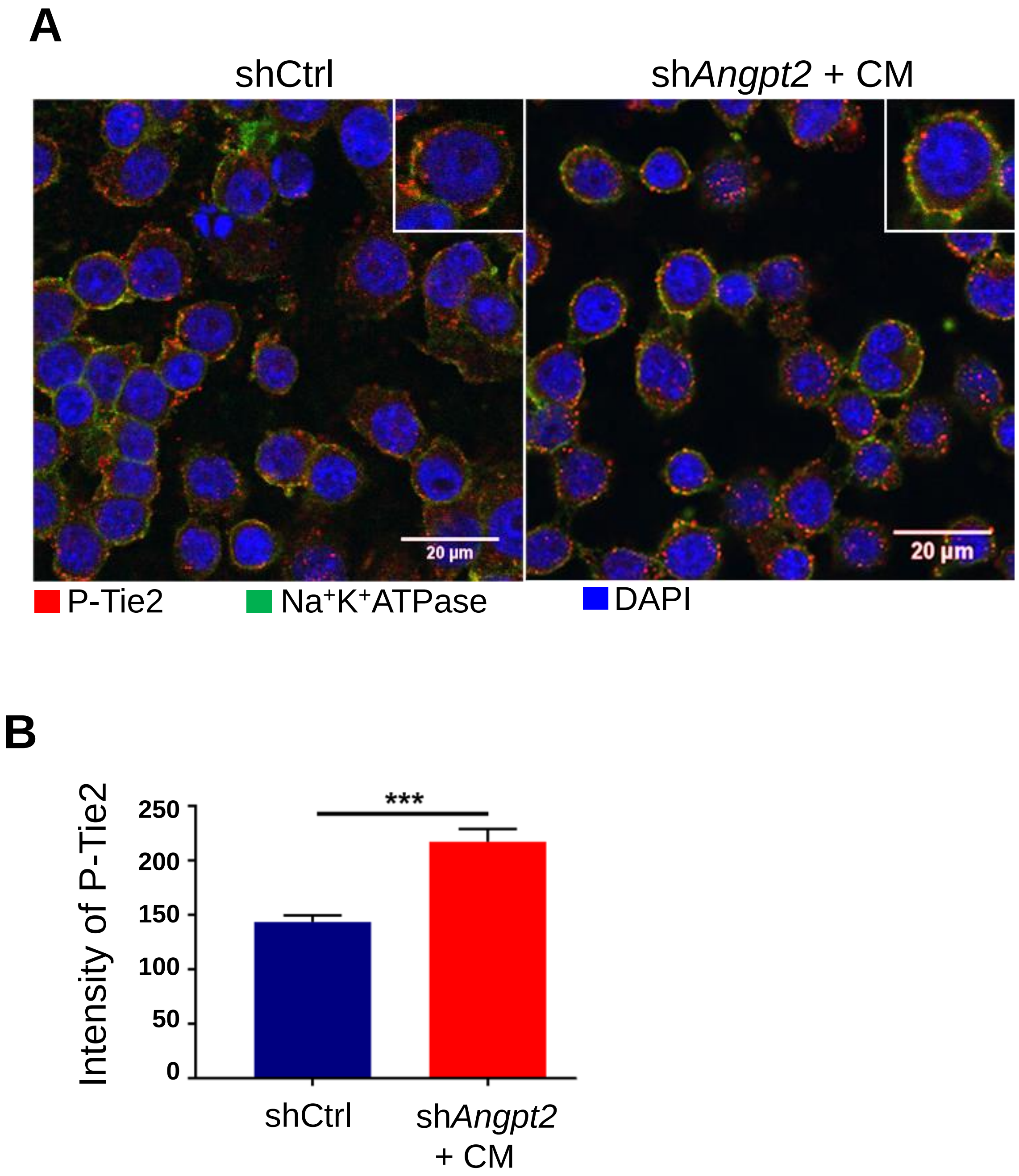

Appendix Figure S12. The conditioned medium (CM) of primary tumor cells induces Tie2 in GH3 cells. (**A**) sh*Angpt2* GH3 cells were incubated with serum-free medium alone *versus* supplemented with 10µl of CM of rat primary PitNETs cells for 15min. Co-IF was performed for both P-Tie2 (Tyr 1102/1108; red) and Na+K+ATPase (green) used as membrane marker. Nuclei were counterstained with DAPI (blue). Original magnification: 400x; scale bar: 20 µm. (**B**) Quantification of staining intensity of P-Tie2 in cells from panel **B**. sh*Angpt2* cells: 143.62±5.98; sh*Angpt2* cells + CM: 217.48±11.35. Intensities are expressed as arbitrary units ± SEM. ***, P<0.0001 (t-test).

Appendix Figure S13

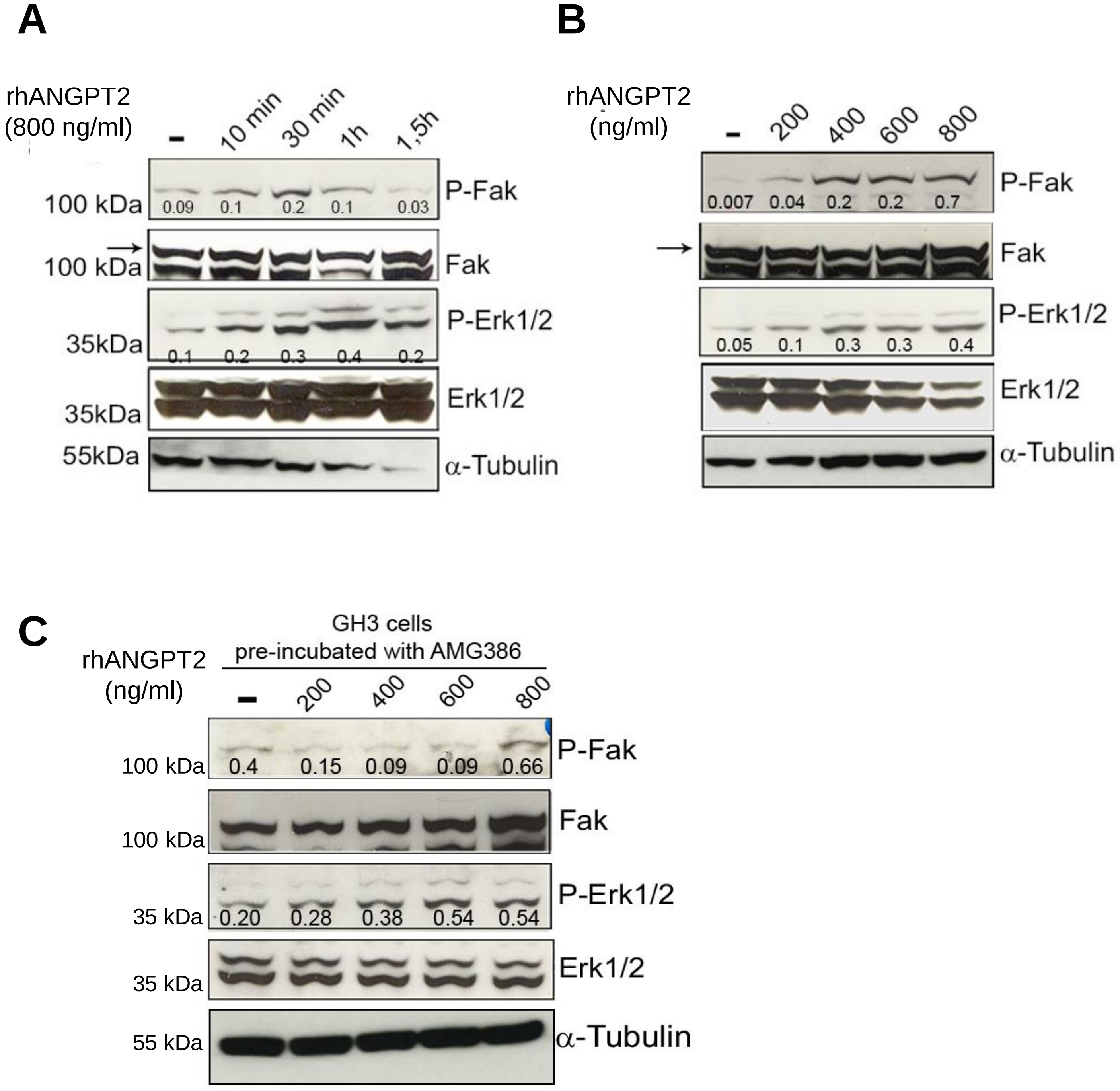

Appendix Figure S13. Pathways downstream of activated Tie2 in GH3 cells. (**A, B**) Serum-starved GH3 cells transfected with siAngpt2 POOLs were stimulated with (**A**) 800 ng/ml rhANGPT2 for 0, 10 min, 30 min, 1h and 1.5h or (**B**) stimulated with the indicated rhANGPT2 doses for 30 minutes. Proteins were extracted and probed to assess the expression of Fak, P-Fak, Erk1/2, P-Erk1/2 (Thr202/Tyr204). (**C**) GH3 parental cells were serum-starved for 24h. AMG386 was added for additional 24h in serum-free medium. Then the medium was changed and cells were stimulated with the indicated concentrations of rhANGPT2 for 30 min. Proteins were extracted and probed as in **A**. (**A**-**C**) The numbers represent the ratio phospho/total proteins. α-Tubulin (1:1000) was used as loading control. Blots shown are representative of 2/3 independent experiments with similar results.

A

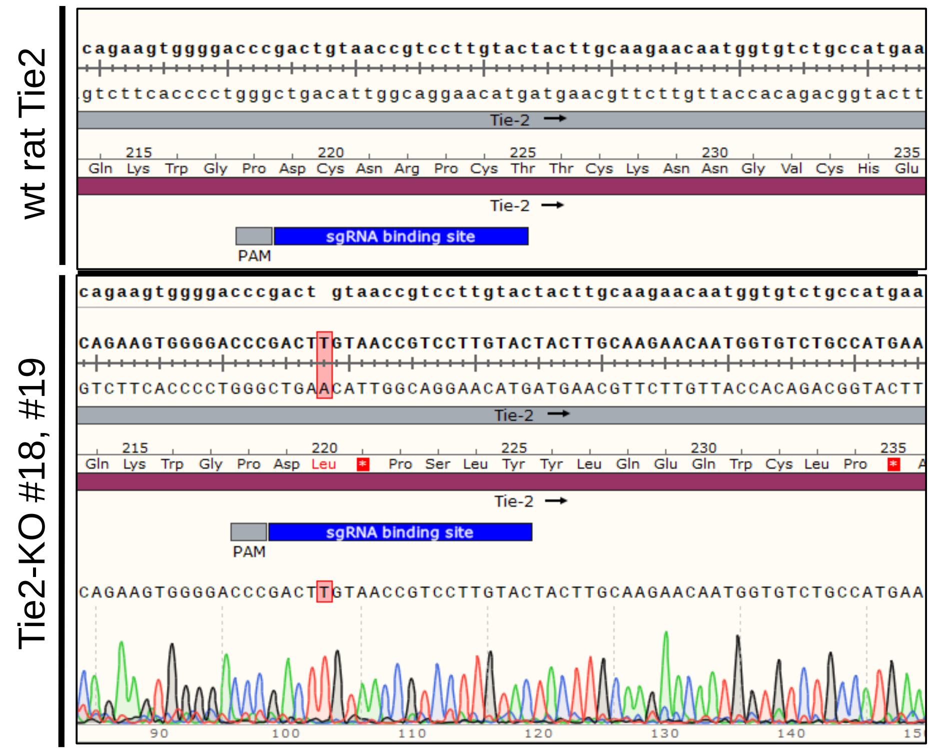

B

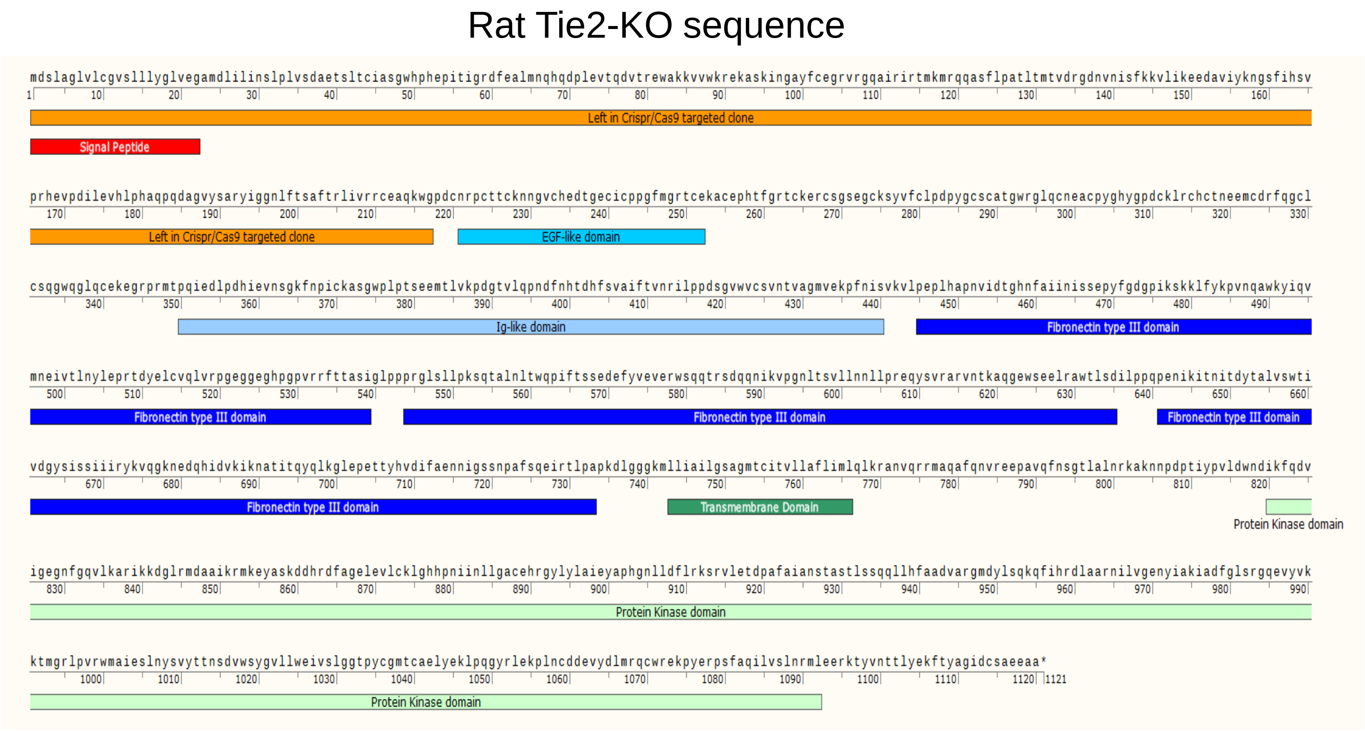

Appendix Figure S14. Tie2 knockout sequence. (**A**) Chromatogram showing the homozygous mutation in the targeted region of rat Tie2. The wild-type (wt) Tie2 targeted sequence is aligned with the sequence of clones #18 and #19 where an additional T was inserted by CRISPR/Cas9 editing (indicated in red). This insertion leads to a stop codon at aa 220 (indicated in red). (**B**) Aminoacid sequence of rat Tie2 protein indicating the various domains and the part of the protein that is left over in the CRISPR-Cas9-targeted clones #18 and #19 (in orange).

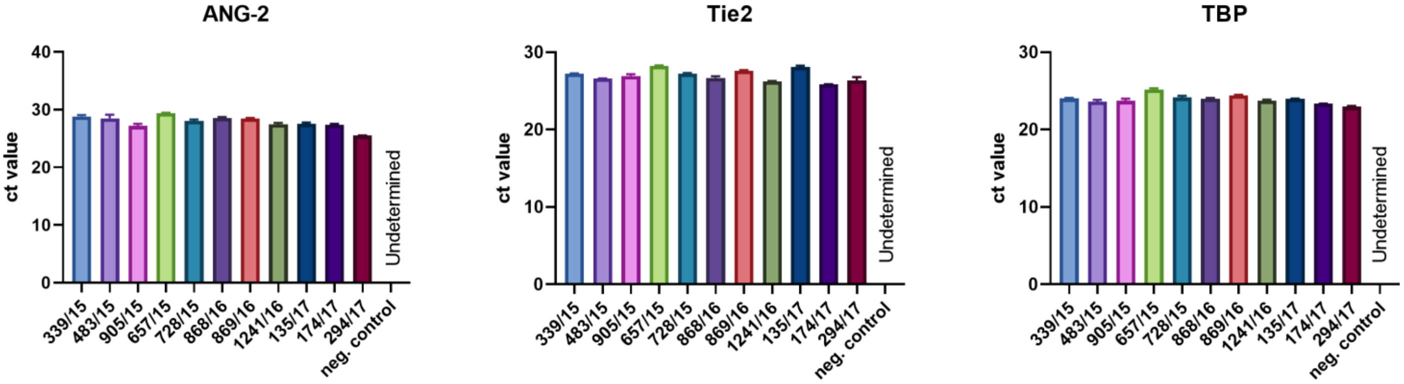

Appendix Figure S15. H**uman NF-PitNETs express ANGPT2 and Tie2.** RNA was extracted from a subset of the primary human PitNETs shown in Fig EV4. qRT-PCR was performed for *ANGPT2*, *Tie2* and the *TBP* housekeeping control gene. Shown are the original Ct values.

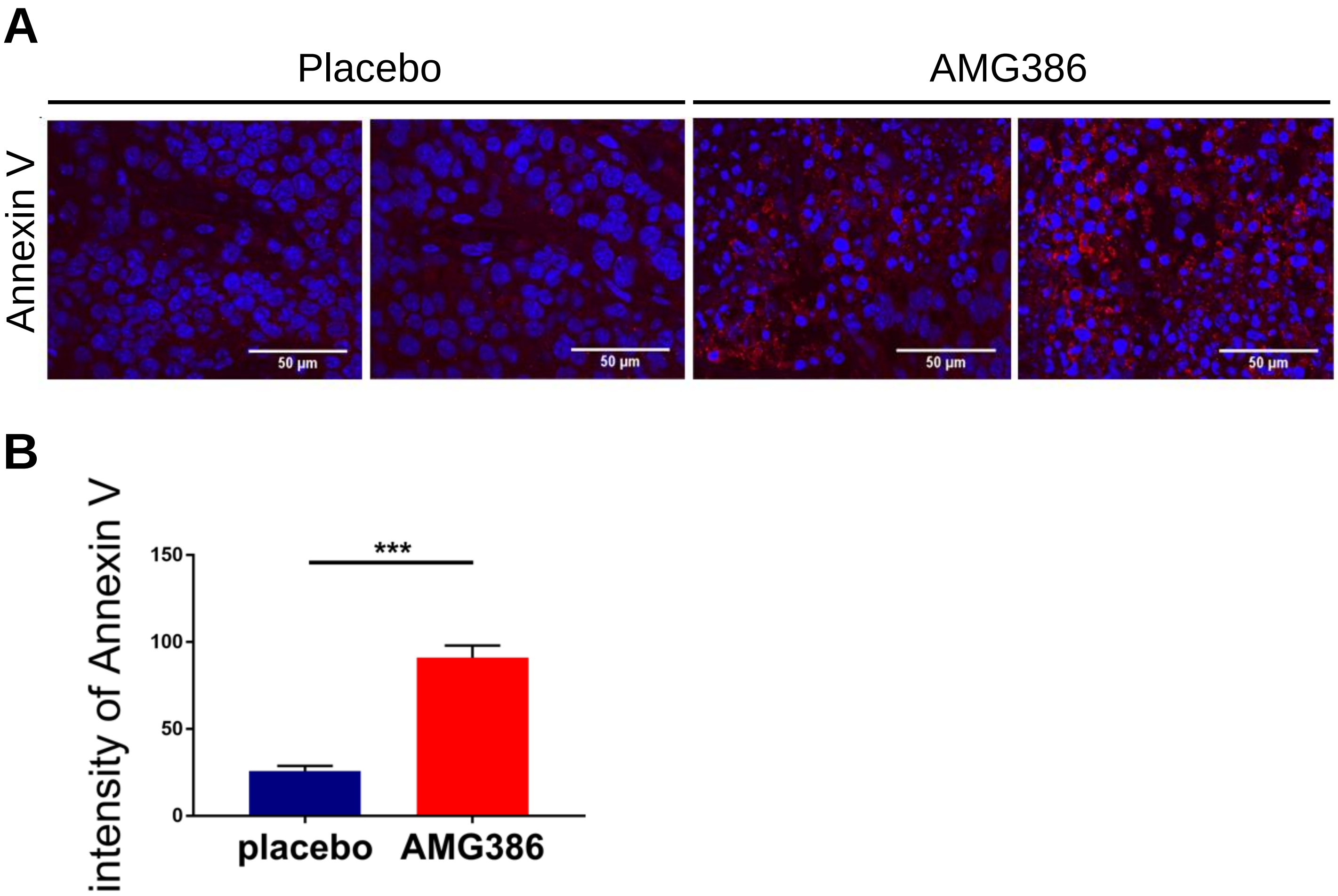

Appendix Figure S16. Expression of Annexin V in tissues from mouse xenografts of GH3 cells treated or untreated with AMG386. (**A**) Expression of Annexin V in mouse xenografts treated with AMG386 or placebo for 21 days. Original magnification: 400x; scale bar: 50 µm. Panels shown are representative of the two treatment groups. (**B**) Quantification of Annexin V intensity from tissues stained as in **A** (n=4/group). Three different areas per tumor sample were analyzed. ***, P<0.001.

Appendix Figure S17

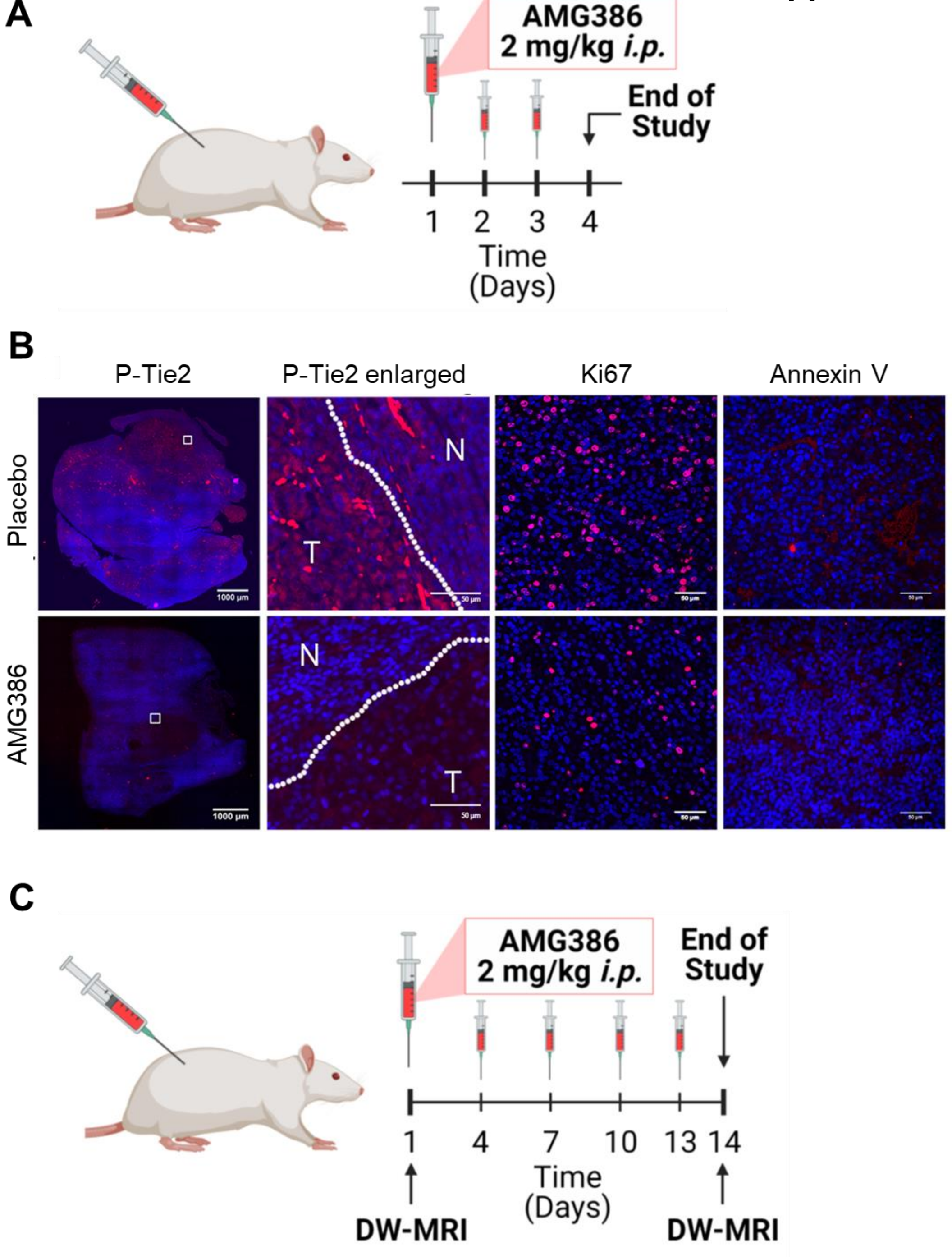

Appendix Figure S17. In vivo treatment of tumor-bearing MENX rats with AMG386.
(**A**) Scheme of the short-term treatment of MENX rats. AMG386 (2 mg/kg body weight) or placebo were administered to 8-month-old mutant rats (with NF-PitNETs) via intraperitoneal (i.p.) injection every day for 3 days. Then, rats were sacrificed and tissues collected for *ex vivo* tissue analyses. (**B**) *Ex vivo* expression of P-Tie2, Ki67 and Annexin V in PitNETs of rats treated with AMG386 or placebo for 3 days. N, normal area; T, tumor area in digitally enlarged sections. Original magnification P-Tie2: 100x; scale bar: 1000µm; Ki67/Annexin V: 200x; scale bar: 50 µm. Pictures were taken with the same exposure time. (**C**) Scheme of the long-term treatment of MENX rats. AMG386 or placebo were administered to 8-month-old mutant rats via i.p. injection every 3 days for 14 days. At day 0, T2-weighted MRI and DW-MRI were performed on 13 rats (8 treated with AMG386 and 5 untreated). At day 14, MRI was performed on the same rats. After the last scan, the rats were sacrificed and the pituitary glands collected for *ex vivo* tissue analyses.

Appendix Table S1: Clinical characteristics of NF-PitNET patients whose plasma was analyzed by ELISA to measure circulating ANGPT2 levels.

Patient ID	Sex	Age	Knosp Grade	Primary/ Recurrence	% Ki67	ANGPT2 (ng/ml)
20/14	M	73	3	Primary	1	2.122
22/14	W	71	2	Primary	2	1.417
76/14	M	37	2	Primary	2	1.457
80/14	M	70	2	Primary	< 1	1.337
93/14	M	46	2	Primary	2.5	2.812
113/14	M	20	3	Primary	1	5.332
124/14	W	57	1	Primary	1	3.177
125/14	W	65	1	Primary	1	1.017
129/14	W	80	2	Primary	1	1.742
139/14	W	66	2	Primary	1.5	1.717
141/14	M	59	2	Primary	1.5	1.712
145/14	W	75	2	Primary	1	1.957
151/14	M	53	3	Primary	1.5	1.772
154/14	M	75	1	Primary	3	2.282
162/14	W	62	3	Primary	2	1.242
184/14	M	62	3	Primary	1	1.412
215/14	W	69	4	Recurrence	1	0.767
229/14	M	79	1	Primary	1.5	1.257
271/14	M	77	2	Primary	3	3.222
278/14	W	54	1	Primary	1.5	0.857
325/14	M	62	3	Primary	< 1	0.892
331/14	M	63	3	Recurrence	< 1	0.814
7/15	M	56	1	Primary	1	1.382
32/15	W	75	1	Recurrence	3	2.947
160/15	W	80	1	Primary	2	2.477
222/15	M	37	4	Primary	< 1	1.317
281/15	W	56	2	Primary	1	0.837
282/15	M	48	1	Primary	1	1.642
309/15	M	55	3	Primary	2	1.482
371/15	W	45	1	Primary	1.5	2.062
420/15	M	50	1	Primary	2	2.582
481/15	M	73	1	Primary	1	1.422
482/15	M	52	1	Primary	1.5	0.762
556/15	W	47	4	Primary	1	1.232
636/15	W	64	1	Primary	1	0.732
793/15	W	76	2	Primary	1	2.092
865/15	M	33	1	Primary	-	1.047
896/15	W	30	4	Recurrence	2	2.602
1150/15	W	33	3	Primary	1	1.362
16/16	M	61	2	Primary	1	1.191
33/16	W	49	1	Primary	1	1.282
44/16	M	73	1	Primary	2	0.566
49/16	W	42	2	Primary	1	3.252
129/16	M	57	2	Primary	1.5	1.401
188/16	M	34	1	Primary	1.5	1.831
199/16	M	73	2	Primary	< 1	1.216
297/16	W	34	1	Primary	2	1.396
327/16	W	57	3	Recurrence	2.5	1.636
347/16	M	63	1	Primary	1	2.121
385/16	M	83	1	Primary	2	1.166
531/16	W	70	2	Primary	1.5	1.611
560/16	M	50	2	Primary	< 1	1.091
643/16	M	59	3	Primary	2	1.036
656/16	M	76	2	Primary	1	0.921
682/16	W	62	3	Primary	1	1.021
697/16	W	60	2	Recurrence	2.5	0.996
767/16	W	75	1	Primary	1	1.676
793/16	M	57	2	Primary	1.5	1.136
900/16	M	76	1	Primary	1	1.306
1173/16	W	70	2	Primary	2	1.191
154/14	M	75	2	Primary	3	3.110
327/16	W	57	3	Recurrence	2-3%	2.291
910/16	W	58	4	Primary	5	2.284
324/17	M	65	1	Primary	3	2.550
704/17	M	33	3	Primary	4	1.253
1098/17	W	22	1	Primary	2-3	8.370
1413/17	M	22	0	Primary	~3% regional 6%	3.151
655/18	M	52	1	Primary	2-3	1.464
790/18	W	68	3	Primary	3	1.717

Appendix Table S2: Clinical characteristics of NF-PitNET patients used for qRT-PCR

Patient ID	Sex	Age	Tumor biology	% Ki67
296	M	56	invasive	2%
286	M	59	invasive	1%
263	M	58	invasive	2%
271	F	44	non-invasive	1%
41	M	57	invasive	2%
224	M	59	non-invasive	2%
276	F	62	invasive	2%
297	M	50	invasive	3%
283	M	47	non-invasive	<1%
285	F	71	non-invasive	1%
288	M	76	invasive	1-2%
289	M	56	invasive	3%
301	F	47	non-invasive	1-2%
267	M	58	invasive	4%

M, male; F, female

Appendix Table S3: The 30 anti-*Angpt2* siRNA oligos included in the siPOOLs

Sense seq	Antisense seq
GCACGGGCAGGAGGCAGTA	TACTGCCTCCTGCCCGTGC
CCAACCAAAGGAGACCCAA	TTGGGTCTCCTTTGGTTGG
GGCTGGACACTATTACTAT	ATAGTAATAGTGTCCAGCC
GGAGACCGACAGCTGCCGA	TCGGCAGCTGTCGGTCTCC
GGAAAACAGAACCCTCAGA	TCTGAGGGTTCTGTTTTCC
GACCCTGCAGCTACACATT	AATGTGTAGCTGCAGGGTC
CGGGACCACATTACTCGGA	TCCGAGTAATGTGGTCCCG
CCTCGACTACGACGACTCA	TGAGTCGTCGTAGTCGAGG
CCAAAGGCACTGCGGGTCT	AGACCCGCAGTGCCTTTGG
CGCCGTGCAGAGGGATGCA	TGCATCCCTCTGCACGGCG
CGGAGCACGTTTCTACAGT	ACTGTAGAAACGTGCTCCG
GCATTCGGACTCTGTCACA	TGTGACAGAGTCCGAATGC
GGGCGCCATGTCAGTGCAT	ATGCACTGACATGGCGCCC
CTTAGGCCTGCACCGCTAA	TTAGCGGTGCAGGCCTAAG
GCACCGCTAACCAACCAAA	TTTGGTTGGTTAGCGGTGC
CACAGGGACAGCATGCATT	AATGCATGCTGTCCCTGTG
GTCAGTGCATGTGGAGACT	AGTCTCCACATGCACTGAC
GCCTGCCTACATTATCAGA	TCTGATAATGTAGGCAGGC
CCTATCTGACGGCTTAGTT	AACTAAGCCGTCAGATAGG
GCATCCAAAGATTAACTCT	AGAGTTAATCTTTGGATGC
GGCACTGAGAGACACCAAT	ATTGGTGTCTCTCAGTGCC
GGCCATGGTTAGAATCACA	TGTGATTCTAACCATGGCC
CAGTTCGTTGTTCCGTCTT	AAGACGGAACAACGAACTG
GACTGACGCACATCACTTA	TAAGTGATGTGCGTCAGTC
CTATTACTATACGGCAAAA	TTTTGCCGTATAGTAATAG
GTCTTGTGCTTGACATGTT	AACATGTCAAGCACAAGAC
GGGTCTGGAGAAATACCTA	TAGGTATTTCTCCAGACCC
CTCCAGGGACTTCTAAGTA	TACTTAGAAGTCCCTGGAG
GTGGGAGTTCATCAGTAAA	TTTACTGATGAACTCCCAC
CTCAGCCTACAATAACTTT	AAAGTTATTGTAGGCTGAG

Appendix Table S4: Angpt2 concentration measured by ELISA in cells supernatant

	GH3	GH3-ANGPT2OE*	R-PitNET cells (n=8)	H-PitNET cells (n=2)	GH3 shctrl	GH3 shC1	GH3 shC2	GH3 shC3
**Cell number**	$9x10^5$	$9x10^5$	$1,5x10^6$	$1,5x10^6$	$9x10^5$	$9x10^5$	$9x10^5$	$9x10^5$
**ng/ml**	2.97±0.05	194.75±0.83	19.5±0.71	5.91±0.36	0.38±0.03	-1.9±0.23	-1.8±0.45	-1.9±0.75

*GH3 cells overexpressing hANGPT2 cDNA as positive control

R-PitNET, rat primary PitNET cells; H-PitNET, human primary PitNET cells

Appendix Table S5: Primary and secondary antibodies used for WB/IHC/IF

Name	Species	Cat. Nr.	Company	Dilution	Used for
anti-Akt	rabbit	9272	Cell Signaling	1:1000	WB
anti-pAkt (S473)	rabbit	4060	Cell Signaling	1:500	WB
anti-Ang1	mouse	Ab133425	Abcam	1:500	WB
anti-Angpt1	goat	AF923	R&D	1:300	IHC
anti-Ang2	goat	AF623	R&D	1:200/1:300	WB/IF/PLA
anti-Ang2	rabbit	ab153934	Abcam	1:400	IHC
anti-Annexin V	rabbit	Ab14196	Abcam	1:200	IF
anti-CD31	rat	130-119-131	Miltenyi Biotech	1:200	FC
anti-CD31	mouse	55502	Becton Dickinson	1:200	IF
anti-c-Myc	rabbit	ab9106	Abcam	1:400	WB
anti-Erk1/2	rabbit	4695	Cell Signaling	1:500	WB
anti-pErk1/2 (Thr202/Tyr204)	rabbit	4376	Cell Signaling	1:1000	WB
anti-Fak	rabbit	3285	Cell Signaling	1:1000	WB
anti-pFak	rabbit	3283	Cell Signaling	1:500	WB
anti-Integrin	rabbit	EP1041Y	Abcam	1:500	WB
anti-Ki67	rabbit	Ab16667	Abcam	1:200	IF
anti-P38	rabbit	9212	Cell Signaling	1:1000	WB
anti-pP38	rabbit	4511	Cell Signaling	1:1000	WB
anti-Tie2	rabbit	PA5-28582	Thermo Scientific	1:500/1:600	WB/IHC+IF/PLA
anti-Tie2	rabbit	sc-324	Santa Cruz	1: 500	WB/IF
anti-Tie2	mouse	05-584	Millipore	1:200	IF
anti-Tie2	rabbit	1300R-PE	BIOSS	1:100	FC
anti-pTie2 (Y992)	rabbit	AF2720	R&D Systems	1:250	WB
anti-pTie2 (pTyr1102/1108)	rabbit	PC449	Millipore	1:3000/1:2000	WB/IHC+IF/PLA
anti-α-tubulin	mause	T5168	Sigma	1:1000	WB
anti-$Na^{+}K^{+}$ATPase	rabbit	Ab76020	Abcam	1:500	IF
anti-$Na^{+}K^{+}$ATPase	mouse	Ab7671	Abcam	1:300	IF
anti-rabbit IgG	donkey	NA934	GE Healthcare	1:3000	WB
anti-mouse IgG	sheep	NA931	GE Healthcare	1:4000	WB
anti-goat IgG	donkey	HAF109	R&D	1:3000	WB
anti-rabbit IgG, FITC	goat	F2765	Invitrogen	1:200	IF
anti-rabbit IgG, Alexa Fluor 555	goat	4413	Cell Signaling	1:200	IF
anti-mouse IgG, FITC	goat	F2761	Invitrogen	1:200	IF
anti-mouse IgG, Alexa Fluor 555	goat	4409	Cell Signaling	1:200	IF
anti-goat IgG, Alexa Fluor 555	donkey	ab150130	Abcam	1:200	IF
anti-guinea-Pig IgG, Alexa Fluor 555	goat	A21435	Invitrogen	1:500	IF
