# Supplementary material for: Angpt2/Tie2 autostimulatory loop controls tumorigenesis
Source: EMBO Mol Med. 2022 Mar 10;14(5):e14364. doi: 10.15252/emmm.202114364 (PMC9081903; doi:10.15252/emmm.202114364)
Supplement: Supplementary file 2 — Expanded View Figures PDF [file EMMM-14-e14364-s002.pdf]

Expanded View Figures

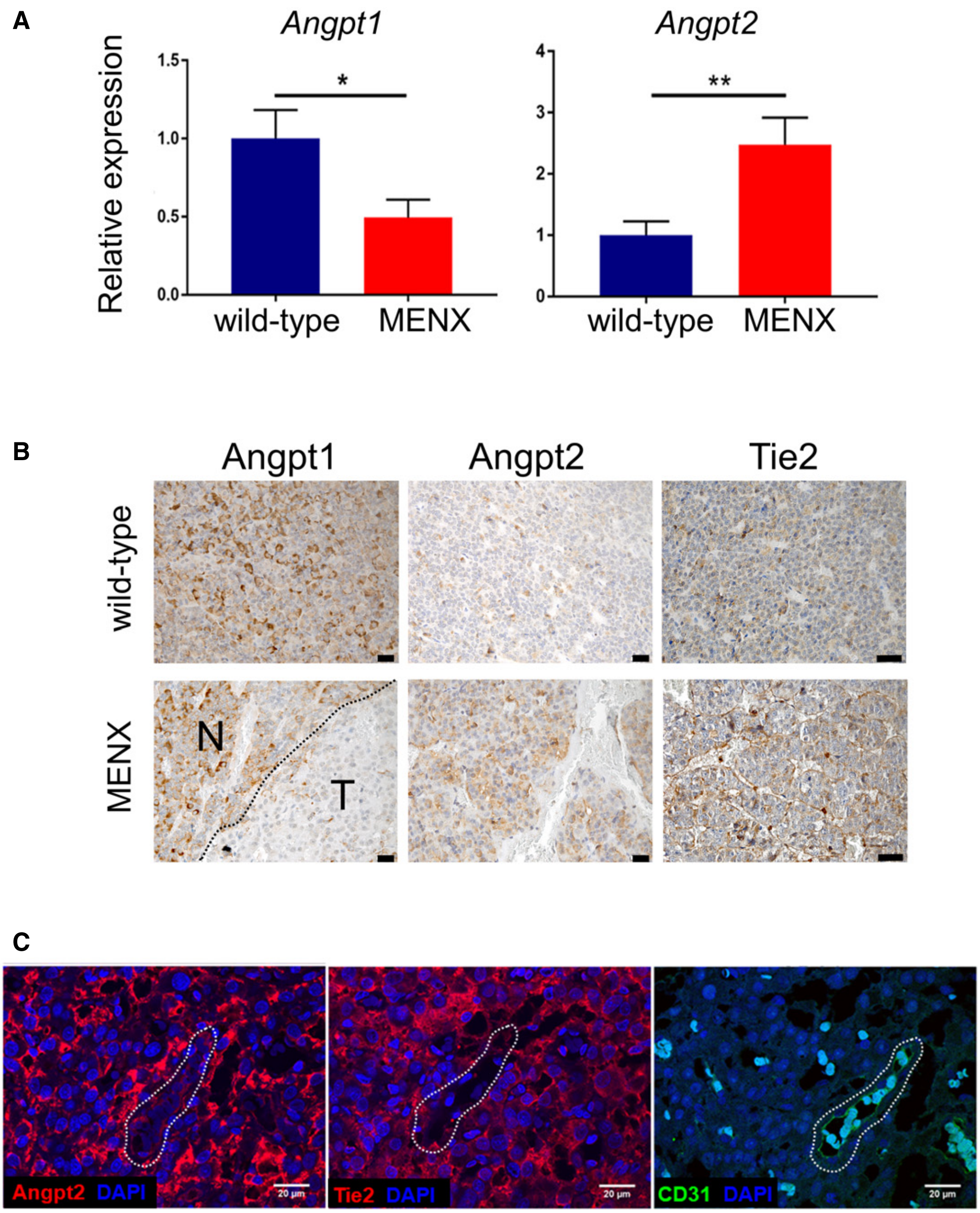

Figure EV1. Angpt2 is expressed in rat primary NF-PitNETs together with Tie2 and Angpt1.

A qRT–PCR for *Angpt1* and *Angpt2* was performed on pituitary samples from tumor-bearing MENX mutant rats (n = 10, 12, respectively) and wild-type control rats (n = 5, 8). Data are expressed as mean ± SEM. *P = 0.049; **P = 0.008 by t-test.

B IHC was performed on pituitary tissues from wild-type (n = 5) and MENX mutant rats (n = 10) using antibodies against Angpt1, Angpt2. Representative stainings are shown. Original magnification: 400×; scale bar: 20 µm. N, normal area; T, tumor area.

C Expression of Angpt2, Tie2, and CD-31 in rat NF-PitNETs and associated ECs (used as positive control). Consecutive tissue sections of rat NF-PitNETs (n = 4) were stained with the indicated antibodies. One representative tumor is shown. The dashed shape indicates the vessel present in the consecutive slides. Original magnification: 200×; scale bar: 20 µm.

 © 2022 The Authors

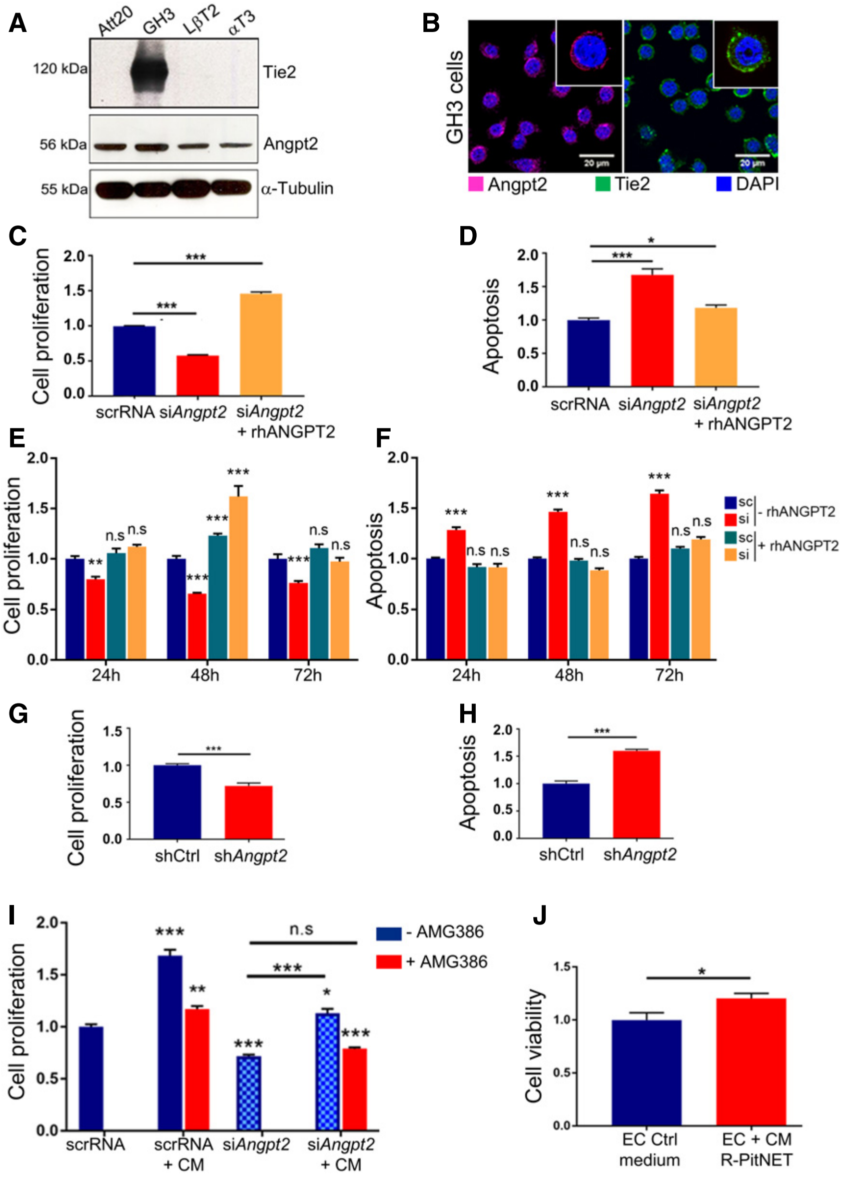

Figure EV2. PitNET cells express Angpt2 and Tie2, and intrinsic Angpt2 promotes PitNET cell survival *in vitro*.

- A Expression of Angpt2 and Tie2 was assessed in Att20, GH3, LβT2 and αT3 cells by western blotting (WB) using specific antibodies. α-Tubulin was included as loading control.
- B Immunofluorescence (IF) of GH3 cells for Angpt2 (red) and Tie2 (green). Nuclei were counterstained with DAPI (blue). Original magnification: 400x; scale bar: 20 µm. Panels shown are representative of 3 independent experiments.
- C Cell proliferation of GH3 cells transfected with si*Angpt2* or scrRNA POOLs and incubated with rhANGPT2 (+rhANGPT2) or left untreated (−rhANGPT2) normalized against scrRNA-transfected cells. ***$P < 0.0001$ (one-way ANOVA).
- D In samples parallel to (C), activated caspase-3/7 was measured to assess for apoptosis. ***$P < 0.0001$; *$P = 0.0411$ (one-way ANOVA).
- E Cell proliferation of GH3 cells transfected with the individual siAngpt2 or scrRNA and incubated with rhANGPT2 (+rhANGPT2) or left untreated (−rhANGPT2) normalized against scrRNA-transfected, untreated (scr-rhANGPT2) cells. n.s., not significant; ***$P < 0.0001$; **$P < 0.002$ (one-way ANOVA).
- F In samples parallel to (E), activated caspase-3/7 was measured to assess for apoptosis. n.s., not significant; ***$P < 0.0001$ (one-way ANOVA).
- G Cell proliferation of GH3-sh*Angpt2* (#2) cells or cells transduced with shCtrl was measured 24h later and normalized against shCtrl. ***$P < 0.0001$ (*t*-test).
- H In samples parallel to (G), activated caspase-3/7 was measured to assess for apoptosis. ***$P < 0.0001$ (*t*-test).
- I GH3 cells transfected with si*Angpt2* or scrRNA POOLs were incubated with conditioned medium (CM) from rat primary PitNET cells for 24 h. CM was pre-incubated with AMG386 (red bars), or left untreated (blue bars). Cell proliferation was measured and normalized against that of scrRNA-transfected cells. n.s., not significant; ***$P < 0.0001$; **$P = 0.00116$; *$P = 0.0192$ by two-way ANOVA.
- J Cell viability of isolated rat primary EC cells incubated with CM from isolated rat primary PitNET cells. The experiments was performed independently 2 times, each with 3 technical replicates. Results are expressed as mean ± SEM. *$P < 0.0368$ (*t*-test). (C–I) Data are expressed as the mean ± SEM of three biological replicates, each with 3–6 technical replicates.

Source data are available online for this figure.

© 2022 The Authors

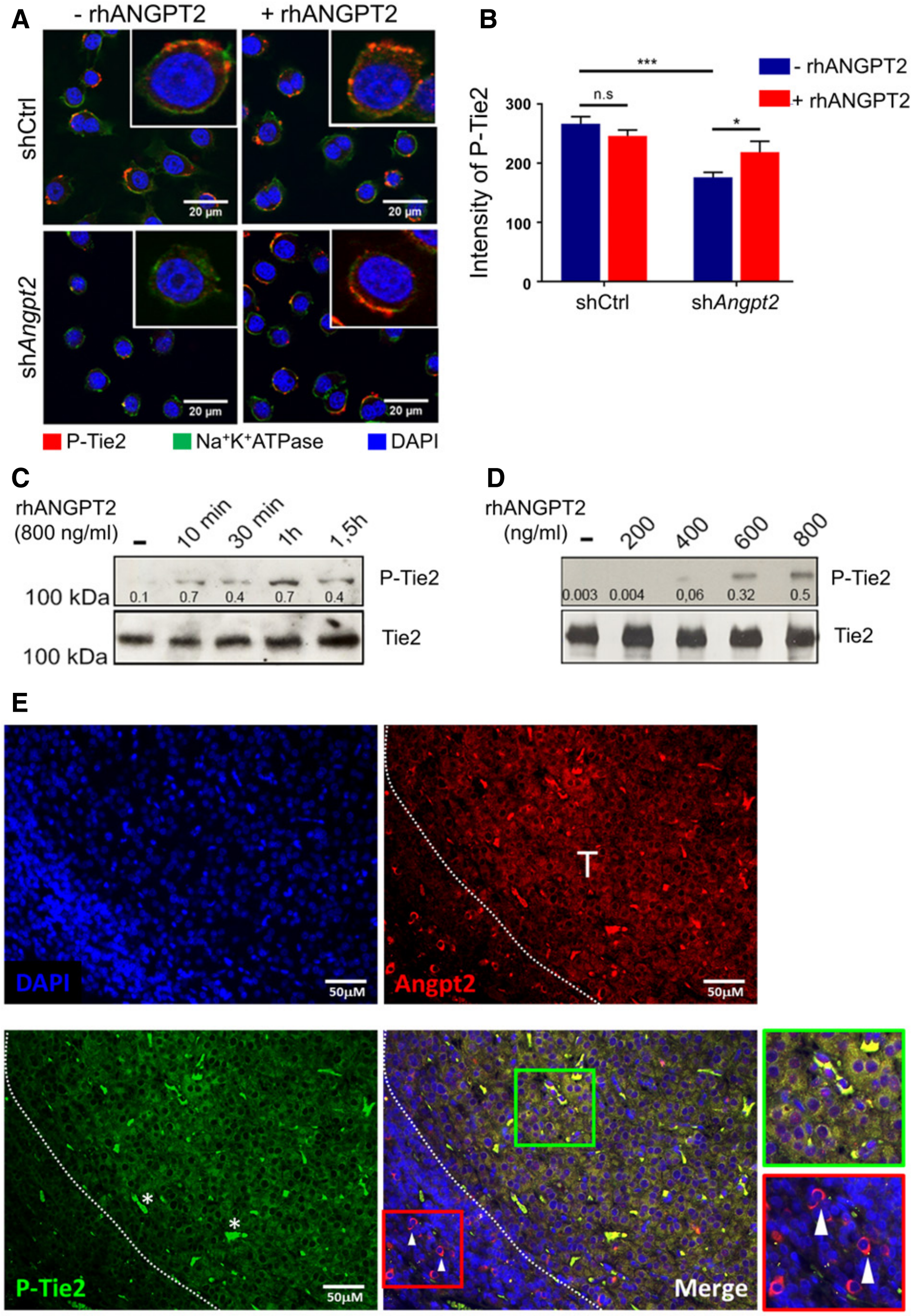

Figure EV3. Tie2 on PitNET cells is phosphorylated and can be stimulated by Angpt2.

A Co-IF for both P-Tie2 (Tyr 1102/1108; red) and for Na^+K^+ ATPase (green), used as plasma membrane marker, of GH3 cells transduced with unspecific shRNA (shCtrl) and sh*Angpt2* (#2) and incubated with rhANGPT2 or left untreated. Original magnification: 400×; scale bar: 20 µm.

B Quantification of P-Tie2 immunostaining intensity in cells shown in (A) shCtrl-transduced GH3 cells (266.71 ± 12.12); in sh*Angpt2* cells (176.04 ± 8.46); in control cells treated with rhANGPT2: shCtrl + rhANGPT2: 246.33 ± 9.79 (versus shCtrl, not significant by *t*-test); in treated knockdown cells: sh*Angpt2* + rhANGPT2: 218.68 ± 18.16 (versus shC2; **P* = 0.0421 by *t*-test). The experiment was performed twice each with three technical replicates. Intensities are expressed as arbitrary units ± SEM. ****P* < 0.0001 by *t*-test.

C Tie2 and P-Tie2 expression in serum-starved GH3 cells transfected with siAngpt2 POOLs and stimulated with rhANGPT2 for the indicated times.

D Tie2 and P-Tie2 expression in cells as in (C) stimulated with the indicated doses of rhANGPT2 for 30 min.

E Co-IF for Angpt2 (red) and P-Tie2 (green) of a representative rat primary NF-PitNET. Nuclei were counterstained with DAPI. White arrows point to Angpt2-positive (P-Tie2 negative) cells in adjacent non-tumor area. Scale bars: 50 µm. T, tumor area.

Data information: (C, D) Blots shown in all panels are representative of three independent experiments. The numbers represent the ratio phospho/total proteins. Source data are available online for this figure.

 © 2022 The Authors

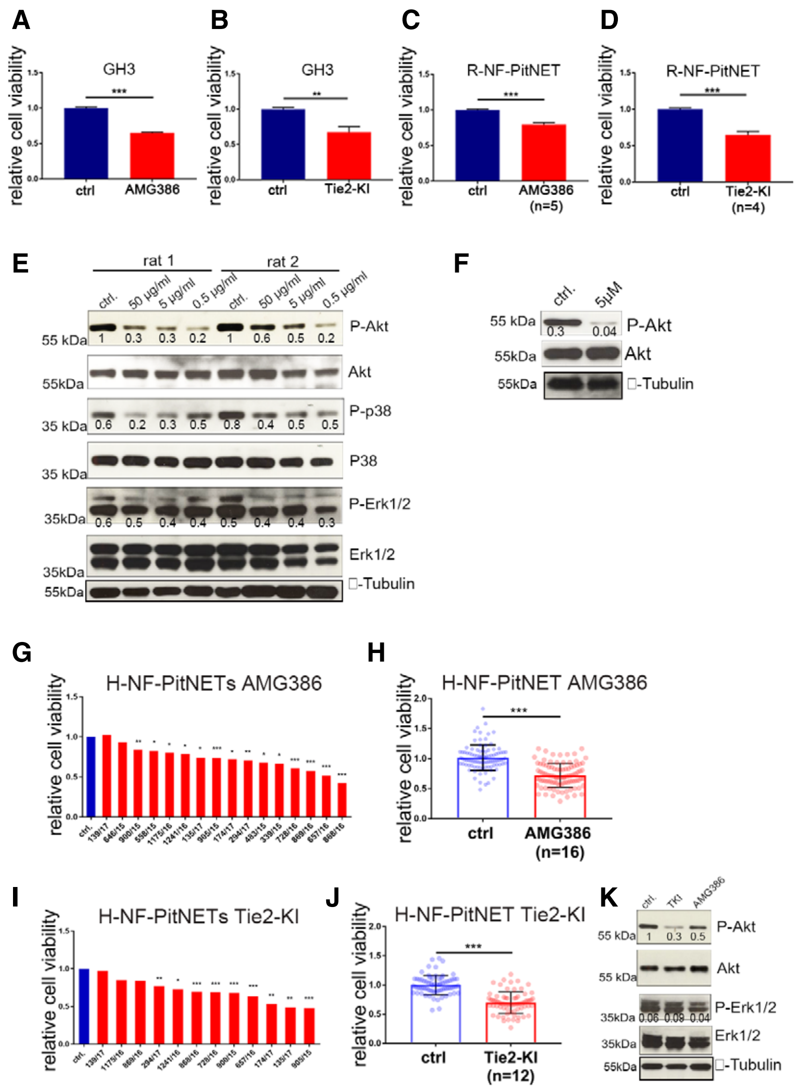

Figure EV4. Pharmacological Ang/Tie2 pathway inhibition reduces the viability of PitNET cell lines, and of rat/human primary cultures *in vitro*.

A, B Cell proliferation of GH3 cells treated with 5 µg/ml AMG386 (A) or 5 µM Tie2-KI (B) or left untreated (ctrl) normalized against untreated cells. ***$P < 0.0001$; **$P = 0.0072$ (*t*-test). Results are expressed as mean ± SEM of three biological replicates with 6 technical replicates.

C, D Cell viability of rat primary PitNET cells (R-PitNET) treated with AMG386 ($n = 5$ rats) (C) or Tie2-KI ($n = 4$ rats) (D) or left untreated (ctrl) normalized against untreated cells. Primary cultures were treated, each with 6 technical replicates. Results are expressed as mean ± SD. ***$P < 0.0001$ (*t*-test).

E Expression of phosphorylated (P) and total Akt, p38 and Erk1/2 in two R-PitNET cultures with enough cells upon treatment with AMG386.

F Expression of phosphorylated (P) and total Akt in one R-PitNET culture treated with Tie2-KI or left untreated (ctrl).

G–J Cell viability of human primary PitNET cultures treated with (G, H) AMG386 ($n = 16$) or (I, J) Tie2-KI ($n = 12$) normalized against untreated control cells for each patient (set to 1). Significance in (G, I): *$P < 0.05$; **$P < 0.01$; ***$P < 0.001$ (by *t*-test, comparing each treated sample with its untreated control). Results in (H) and (J) are expressed as mean ± SEM of the normalized values of 6 technical replicates for each samples. Each dot represents the relative cell viability of one technical replicate. Significance in (H, J) ***$P < 0.0001$ (*t*-test).

K Example of responsive H-PitNET treated with AMG386 or Tie2-KI. Total proteins were extracted and probed for P- and total Akt and Erk1/2. Anti-α-tubulin antibody as used to check for equal loading. The blot shown is representative of three independent cultures.

Data information: (E, F) Blots shown are representative of 3 independent experiments. (E, F, K) The numbers below the bands represent the ratio phospho/total proteins.
Source data are available online for this figure.
