# Supplementary material for: Angpt2/Tie2 autostimulatory loop controls tumorigenesis
Source: EMBO Mol Med. 2022 Mar 10;14(5):e14364. doi: 10.15252/emmm.202114364 (PMC9081903; doi:10.15252/emmm.202114364)

Figure 3A, left panel

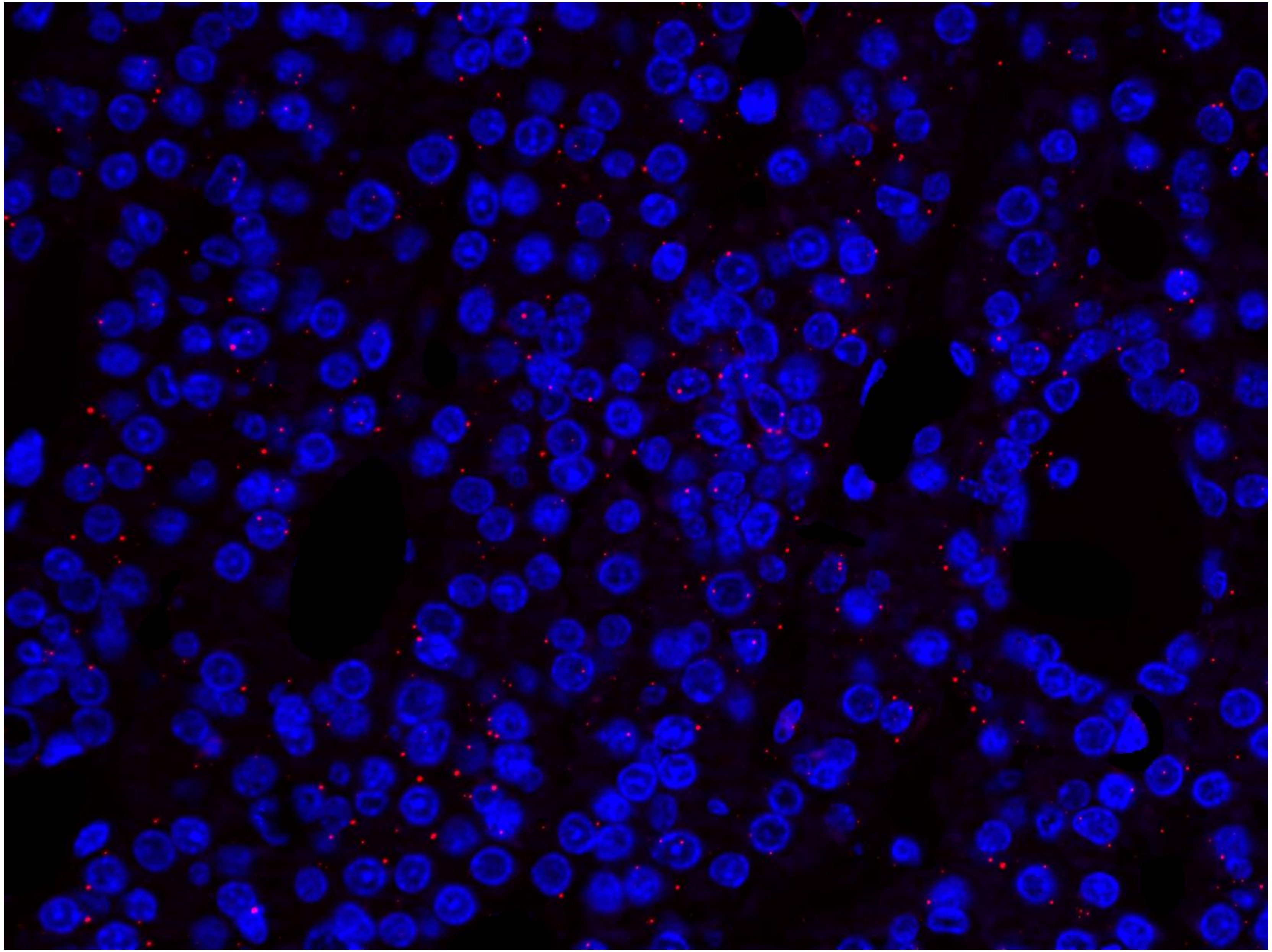

Figure 3A, central panel

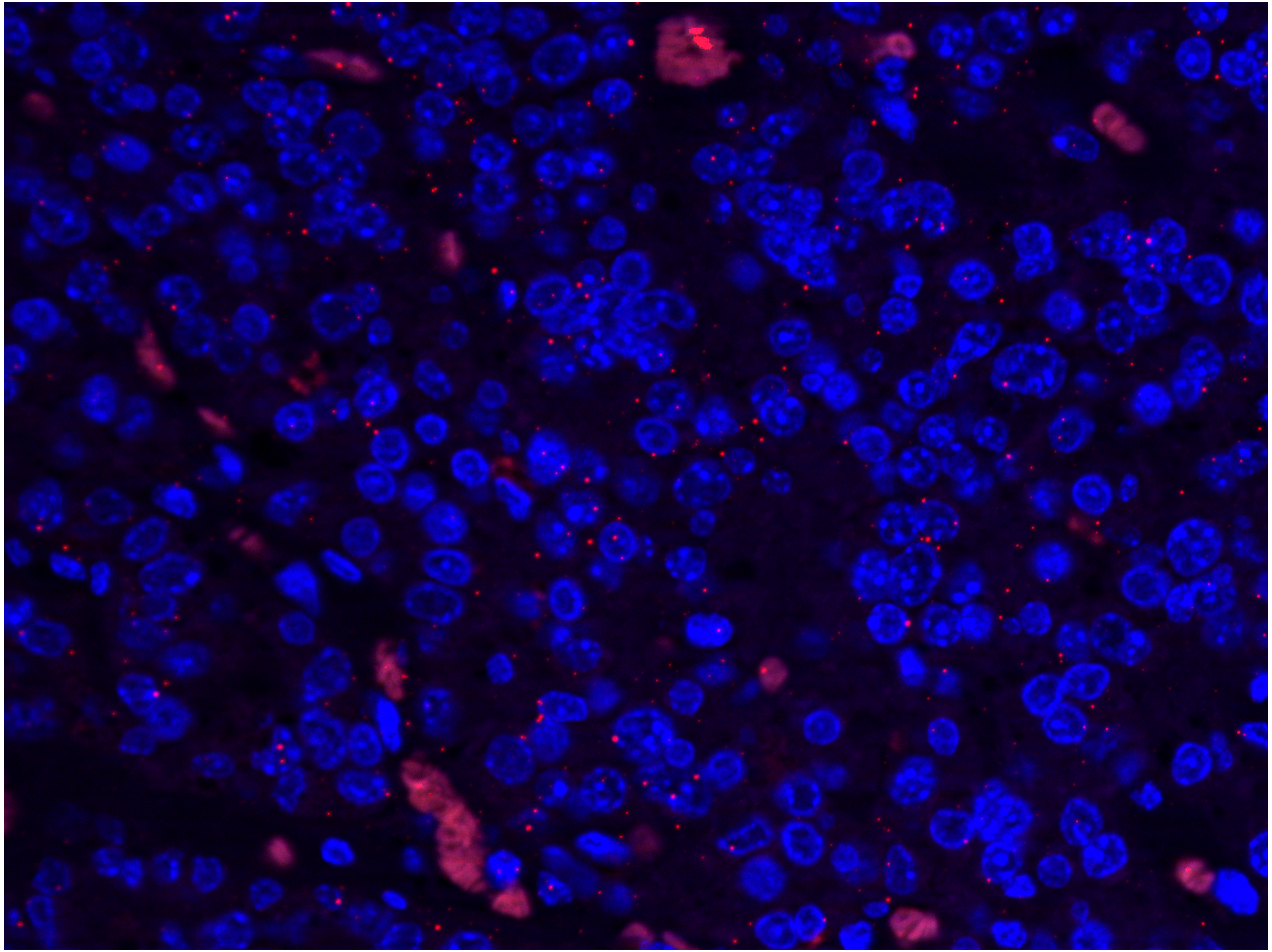

Figure 3A, right panel

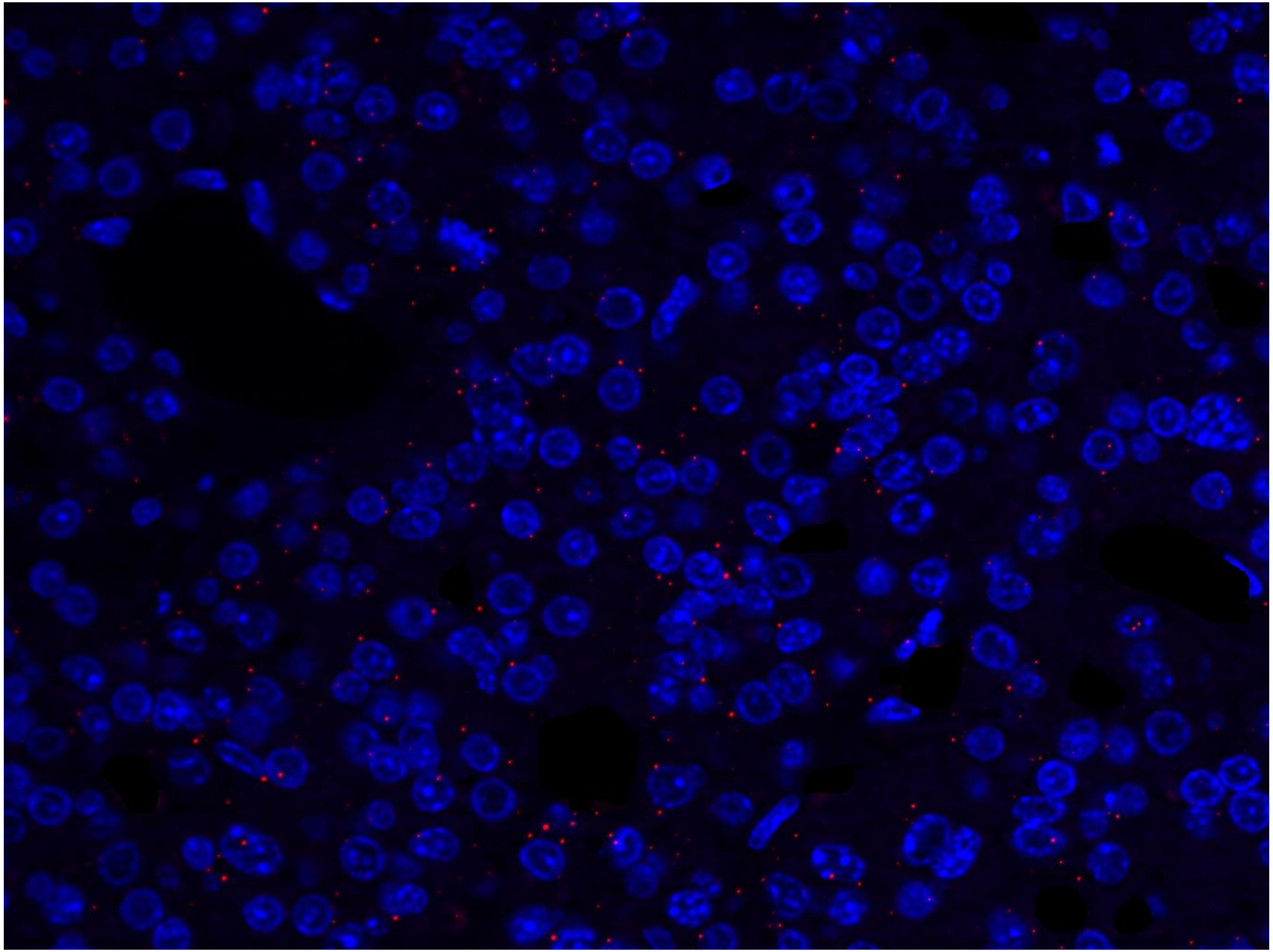

Figure 3C
Left panel

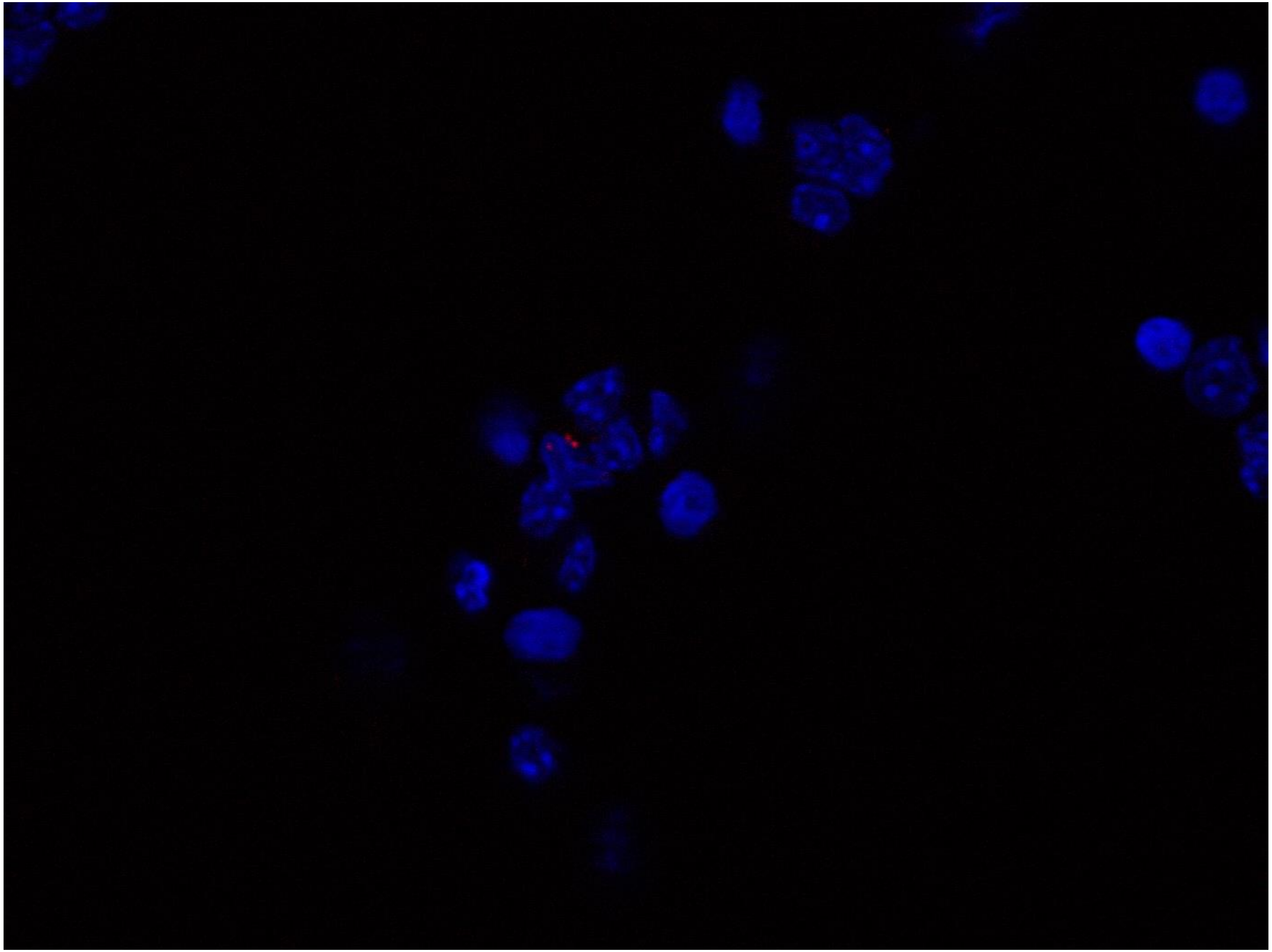

Figure 3C
Right panel

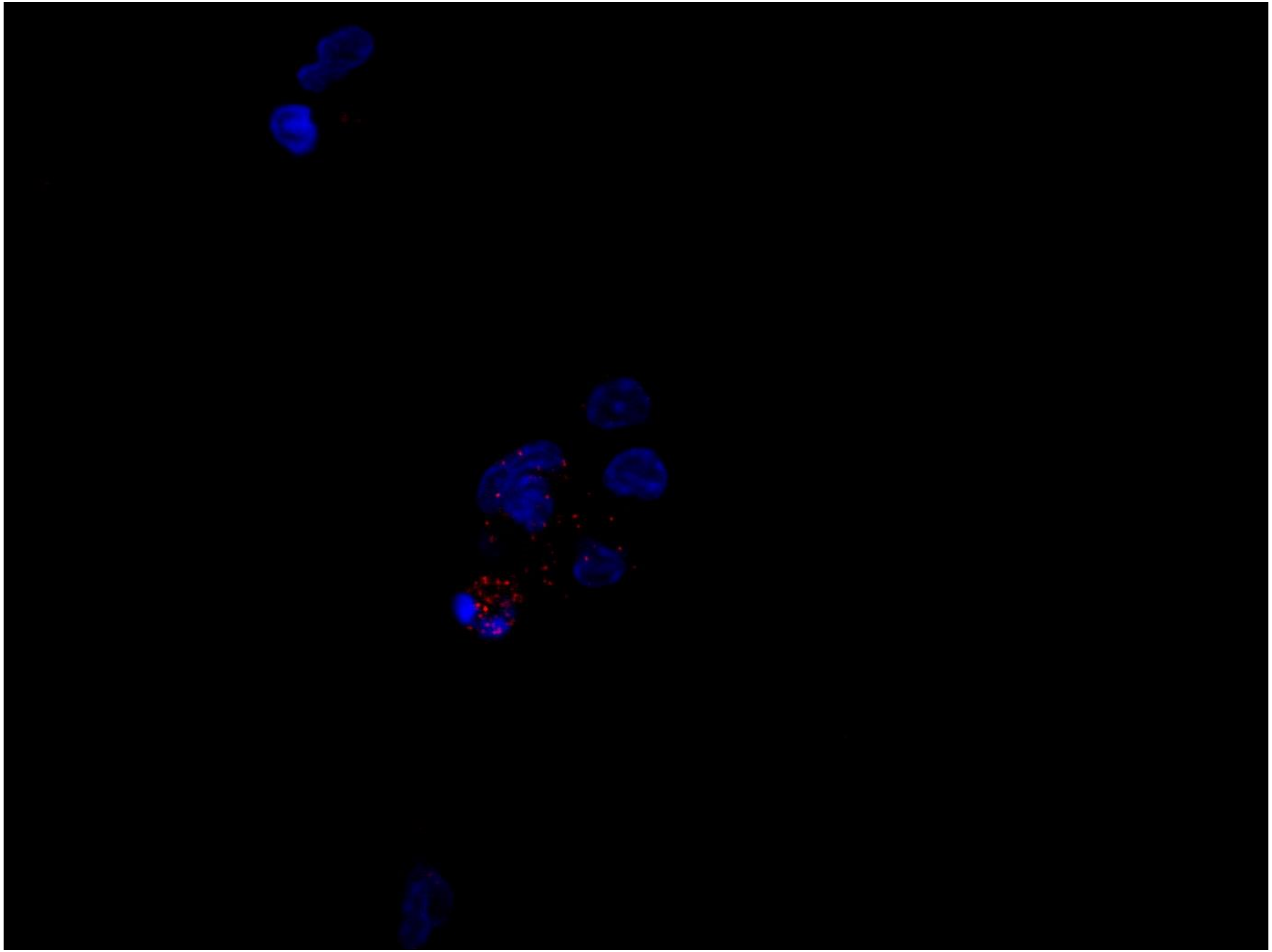

Supplement: Supplementary file 5 — Source Data for Figure 3 [file EMMM-14-e14364-s006.zip › EMM_2021-14364_uncropped_IF_v2.pdf]
