# Supplementary material for: Angpt2/Tie2 autostimulatory loop controls tumorigenesis
Source: EMBO Mol Med. 2022 Mar 10;14(5):e14364. doi: 10.15252/emmm.202114364 (PMC9081903; doi:10.15252/emmm.202114364)

Main figures

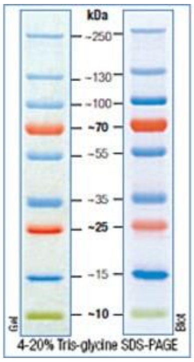

Prestained dual marker

In the following gels, the bands indicated in red on the films correspond to the 70 kDa or the 25 kDa band.

Full unedited gel for Figure 4B:

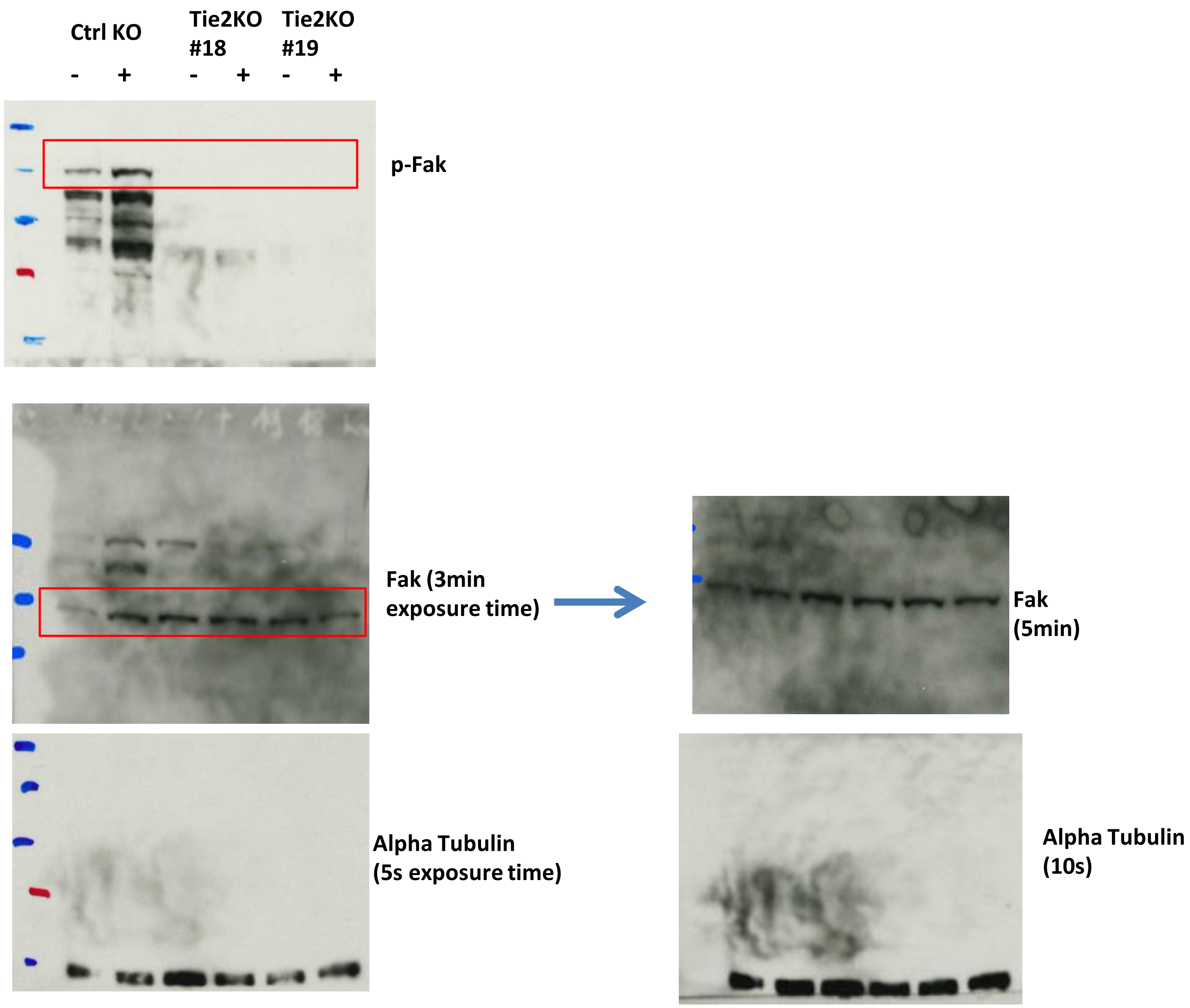

Supplement: Supplementary file 6 — Source Data for Figure 4 [file EMMM-14-e14364-s003.zip › EMM_2021_14364_Uncropped_Fig_4B.pdf]
