# Supplementary material for: Angpt2/Tie2 autostimulatory loop controls tumorigenesis
Source: EMBO Mol Med. 2022 Mar 10;14(5):e14364. doi: 10.15252/emmm.202114364 (PMC9081903; doi:10.15252/emmm.202114364)
Supplement: Supplementary file 8 [file EMMM-14-e14364-s004.pdf]

EMBO Molecular Medicine

Angpt2/Tie2 autostimulatory loop controls tumorigenesis

Ninelia Karabid, Tobias Wiedemann, Sebastian Gulde, Hermine Mohr, Renu Segaran, Julia Geppert, Maria Rohm, Giovanni Vitale, Germano Gaudenzi, Alessandra Dicitore, Donna Ankerst, Yiyao Chen, Rickmer Braren, Georg Kaissis, Franz Schilling, Mathias Schillmaier, Graeme Eisenhofer, Stephan Herzig, Federico Roncaroli, Jürgen Honegger, and Natalia Pellegata
DOI: 10.15252/emmm.202114364

Corresponding author: Natalia Pellegata (natalia.pellegata@helmholtz-muenchen.de)

Review Timeline:

Submission Date:	1st Apr 21
Editorial Decision:	4th May 21
Revision Received:	22nd Nov 21
Editorial Correspondence:	14th Dec 21
Author Correspondence:	23rd Dec 21
Editorial Decision:	11th Jan 22
Revision Received:	24th Jan 22
Editorial Decision:	26th Jan 22
Revision Received:	4th Feb 22
Accepted:	8th Feb 22

Editor: Lise Roth

Transaction Report:

(Note: With the exception of the correction of typographical or spelling errors that could be a source of ambiguity, letters and reports are not edited. Depending on transfer agreements, referee reports obtained elsewhere may or may not be included in this compilation. Referee reports are anonymous unless the Referee chooses to sign their reports.)

4th May 2021

Dear Prof. Pellegata,

Thank you for the submission of your manuscript to EMBO Molecular Medicine, and please accept my apologies for the delay in getting back to you, which is due to the fact that one referee needed more time to complete his/her report. We have now received feedback from the three reviewers who agreed to evaluate your manuscript. As you will see from the reports below, the referees acknowledge the interest and novelty of the study and are overall supporting publication of your work pending appropriate revisions (in particular regarding data and writing quality, as well as issues with nomenclature).

Addressing the reviewers' concerns in full will be necessary for further considering the manuscript in our journal, and acceptance of the manuscript will entail a second round of review. EMBO Molecular Medicine encourages a single round of revision only and therefore, acceptance or rejection of the manuscript will depend on the completeness of your responses included in the next, final version of the manuscript. For this reason, and to save you from any frustrations in the end, I would strongly advise against returning an incomplete revision.

When submitting your revised manuscript, please carefully review the instructions that follow below. Failure to include requested items will delay the evaluation of your revision:

1) A .docx formatted version of the manuscript text (including legends for main figures, EV figures and tables). Please make sure that the changes are highlighted to be clearly visible.

2) Individual production quality figure files as .eps, .tif, .jpg (one file per figure).

3) A .docx formatted letter INCLUDING the reviewers' reports and your detailed point-by-point responses to their comments. As part of the EMBO Press transparent editorial process, the point-by-point response is part of the Review Process File (RPF), which will be published alongside your paper.

4) A complete author checklist, which you can download from our author guidelines (https://www.embopress.org/page/journal/17574684/authorguide#submissionofrevisions). Please insert information in the checklist that is also reflected in the manuscript. The completed author checklist will also be part of the RPF.

5) Before submitting your revision, primary datasets produced in this study need to be deposited in an appropriate public database (see https://www.embopress.org/page/journal/17574684/authorguide#dataavailability).
Please remember to provide a reviewer password if the datasets are not yet public.
The accession numbers and database should be listed in a formal "Data Availability " section (placed after Materials & Method).
Please note that the Data Availability Section is restricted to new primary data that are part of this study.

*** Note - All links should resolve to a page where the data can be accessed. ***

6) We would also encourage you to include the source data for figure panels that show essential data. Numerical data should be provided as individual .xls or .csv files (including a tab describing the data). For blots or microscopy, uncropped images should be submitted (using a zip archive if multiple images need to be supplied for one panel). Additional information on source data and instruction on how to label the files are available at
.

7) Our journal encourages inclusion of *data citations in the reference list* to directly cite datasets that were re-used and obtained from public databases. Data citations in the article text are distinct from normal bibliographical citations and should directly link to the database records from which the data can be accessed. In the main text, data citations are formatted as follows: "Data ref: Smith et al, 2001" or "Data ref: NCBI Sequence Read Archive PRJNA342805, 2017". In the Reference list, data citations must be labeled with "[DATASET]". A data reference must provide the database name, accession number/identifiers and a resolvable link to the landing page from which the data can be accessed at the end of the reference. Further instructions are available at .

8) We replaced Supplementary Information with Expanded View (EV) Figures and Tables that are collapsible/expandable online. A maximum of 5 EV Figures can be typeset. EV Figures should be cited as 'Figure EV1, Figure EV2" etc... in the text and their respective legends should be included in the main text after the legends of regular figures.

- For the figures that you do NOT wish to display as Expanded View figures, they should be bundled together with their legends in a single PDF file called *Appendix*, which should start with a short Table of Content. Appendix figures should be referred to in

the main text as: "Appendix Figure S1, Appendix Figure S2" etc.

- Additional Tables/Datasets should be labeled and referred to as Table EV1, Dataset EV1, etc. Legends have to be provided in a separate tab in case of .xls files. Alternatively, the legend can be supplied as a separate text file (README) and zipped together with the Table/Dataset file.
See detailed instructions here:
.

9) The paper explained: EMBO Molecular Medicine articles are accompanied by a summary of the articles to emphasize the major findings in the paper and their medical implications for the non-specialist reader. Please provide a draft summary of your article highlighting
- the medical issue you are addressing,
- the results obtained and
- their clinical impact.

This may be edited to ensure that readers understand the significance and context of the research. Please refer to any of our published articles for an example.

10) For more information: There is space at the end of each article to list relevant web links for further consultation by our readers. Could you identify some relevant ones and provide such information as well? Some examples are patient associations, relevant databases, OMIM/proteins/genes links, author's websites, etc...

11) Every published paper now includes a 'Synopsis' to further enhance discoverability. Synopses are displayed on the journal webpage and are freely accessible to all readers. They include a short stand first (maximum of 300 characters, including space) as well as 2-5 one-sentences bullet points that summarizes the paper. Please write the bullet points to summarize the key NEW findings. They should be designed to be complementary to the abstract - i.e. not repeat the same text. We encourage inclusion of key acronyms and quantitative information (maximum of 30 words / bullet point). Please use the passive voice. Please attach these in a separate file or send them by email, we will incorporate them accordingly.

Please also suggest a striking image or visual abstract to illustrate your article as a png file 550 px-wide x 400-px high.

12) As part of the EMBO Publications transparent editorial process initiative (see our Editorial at http://embomolmed.embopress.org/content/2/9/329), EMBO Molecular Medicine will publish online a Review Process File (RPF) to accompany accepted manuscripts.
In the event of acceptance, this file will be published in conjunction with your paper and will include the anonymous referee reports, your point-by-point response and all pertinent correspondence relating to the manuscript. Let us know whether you agree with the publication of the RPF and as here, if you want to remove or not any figures from it prior to publication.
Please note that the Authors checklist will be published at the end of the RPF.

I look forward to receiving your revised manuscript.

Yours sincerely,

Lise Roth

Lise Roth, PhD
Editor
EMBO Molecular Medicine

To submit your manuscript, please follow this link:

Link Not Available

*Additional important information regarding Figures

Each figure should be given in a separate file and should have the following resolution:
Graphs 800-1,200 DPI
Photos 400-800 DPI
Colour (only CMYK) 300-400 DPI"

Figures are not edited by the production team. All lettering should be the same size and style; figure panels should be indicated by capital letters (A, B, C etc). Gridlines are not allowed except for log plots. Figures should be numbered in the order of their appearance in the text with Arabic numerals. Each Figure must have a separate legend and a caption is needed for each panel.

*Additional important information regarding figures and illustrations can be found at https://bit.ly/EMBOPressFigurePreparationGuideline

***** Reviewer's comments *****

Referee #1 (Remarks for Author):

This manuscript appears to have an interesting finding, but it also has so many problems, extensive revision is required before one can really evaluate it for publication. The presentation of the data also requires quite extensive professional rewriting. Only then can this potentially interesting manuscript be properly evaluated for publication in EMM.

1. From the data shown it appears that Tie2 expression is heterogeneous in the tumors. Flow cytometry and comparison with Tie2 expression in ECs could resolve this issue and give an idea of the relative expression of Tie2 in tumor cells vs ECs. - After tumor treatment with the inhibitors, do the authors see treatment-escaped Tie2 or Ang2 negative cells?

2. AMG386 inhibits both ANG1 and ANG2 and the tyrosine kinase inhibitor very likely also inhibits both Tie2 and Tie1. What is the evidence that the inhibition in vivo is Ang2 specific?

3. At least in some of the signal transduction studies, it would be good to use Ang1 as control ligand for Tie2. - Is Tie1 or VE-PTP expressed in PitNET cells?

4. What exactly are the cells expressing Ang2 in the normal pituitary? Are they the origin of PitNETs? Are there Ang2 plus Ang1 positive cells and if so, what are their ratios and are there any coexpressing cells?

5. It seems that this manuscript has been submitted previously to another journal, and the authors have not had the energy to streamline their report before resubmission. The manuscript text should be shortened considerably, for example, the abstract is about twice the 175 word limit. The figure legends are much too long! They repeat material that should be presented in the materials and methods. The writing should be edited into a fluent text, crystallizing only the essential findings, which would be easier for the readers to digest. (The authors could perhaps use help from a professional editor).

6. The figures are congested, forcing the miniaturization of many of the immunohistochemical panels, which are of variable quality. The authors should prune the material and transfer some of it to the supplementary figures, concentrating only on the major and clear findings in those figures that should appear in print.

7. Figure 1 has miniaturized texts below the x-axis in panel c; and use much larger text in panel a, for example. In panel i, it is very difficult to find out where the mentioned vessel is located and the CD31 color code is misleading. A general recommendation is not to vary text size by more than a few points and have everything legible in the final reproduction-sized figure.

8. In Figure 2b, it is impossible to say whether the Tie2 receptors are on the cell surface or internalized into some kind of vesicles near the plasma membrane. Flow cytometry of cell surface-stained control and tumor cells should be shown.

9. Figure 3e could be moved to the end of the discussion. The few PLA signals in Figure f could be outside of the cells, and some may be in the cell-free area. A more convincing presentation should be shown with quantification of the signals.

10. Figure 4 panels g and h are not very convincing. For example, P-FAK signal is increased at 30 min, but how reproducible is this? Some of the data here is not critical for the main conclusions of the manuscript. Again, I would like to suggest that the authors prune some of the material in panels e-h out altogether or transfer it to the supplement.

11. Because of the poor resolution of the very small text in panel a of Figure 5, a and b could be also moved to the supplement.

12. The layout should be also improved in Figure 7. For example, panel d could be moved to the supplement, the schematics showing rodent injection could be removed from the figure, thus allowing expansion of panel a, where the text is too small.

13. Panel F in Supplementary Figure 2 is of unacceptable quality. Please show staining of EC islands or confluent EC layers. Panel d in the same figure is too small for the resolution of cellular detail. The text does not specify what alpha subunit is.

14. The panel B in Supplementary figure 5 is supposed to show "Ang-2 (magenta), Tie-2 (green) and P-Tie-2 (red)", but the staining for Ang-2 and Tie-2 are not shown. The immunofluorescent staining in panel C of Supplementary figure 5 is impossible to see. The Tie-2 phosphorylation should be shown by western blotting.

15. Supplementary figure S8 lacks a negative control.

16. In Table S5, the negative values lack an explanation.

17. Statistical analysis. P-values for Figure 2J and 4B are given by t-tests although there are 4 groups in both figures. It is not clear how the statistical analysis was done for Figures 6G and 6I.

18. In Figure 1, the authors show that human PitNETs express both ANG-1 and ANG-2, but on page 12 the authors justify focusing on the interaction between Tie-2 and Ang-2 by saying that "PitNET cells express and secrete a bioactive Ang-2, but do not express Ang-1 ....". This is not correct. The putative role of Ang-1 should be considered and discussed especially since AMG-386 inhibits both Ang-1 and Ang-2 signaling.

19. The authors discuss cytoplasmic and membrane-bound protein expression in several places, but the low resolution of the IF images does not support such conclusions.

20. In the Supplementary figure 3A, the authors show Ang-1 and Ang-2 expression in GH3 and HUVEC cells. Obviously, the authors show cell lysates and not the supernatants. HUVECs show two bands for Ang-2: the premature 55 kDa form and the mature (to be secreted) form of about 70 kDa. GH3 cells do not have the mature form. Please, show the Ang-2 secretion from GH3 cell by analyzing the supernatants.

21. For proximity-ligation assay (PLA, Fig. 3f, g) the authors should use a positive control, for example, cultured endothelial cells. This would show how much Ang2/Tie2 interaction (intra/extracellular?) occurs in cells with known expression of Tie2. The current panels show very few dots. Every dot should represent a single interaction. Does the result mean that the cells express very little or no Tie2?

22. These PLA data should be co-stained for EC markers to show how much the Ang2/Tie2 interaction occurs on tumor cells vs. ECs of blood capillaries.

23. In Fig. 4, the authors show that Tie2 kinase inhibitor surprisingly activates ERK1/2 phosphorylation. The effect of Tie2 kinase inhibitor in GH3 cells should be compared to its effect on ECs.

24. Why does Ang2 treatment cause pERK1/2 phosphorylation in Figs. 4e and f, but not in Fig. 4h? Both represent the 30 min timepoint (panels e and h) and the concentrations are the same (panels f and h).

25. It seems that intracellular signaling in the H-PitNET cells differs from that in GH3 cells. For example, pERK1/2 is not increased by the TKI treatment (Fig. 6k vs. 4g, h). Is it possible that Tie2 kinase regulate distinct processes (e.g. proliferation, survival) in the GH3 vs H-PitNET cells?

26. Does the endogenous Ang2 activate Tie2 intracellularly, in secretory vesicles? This could compromise the efficacy of the anti-Ang2 treatment at least to some degree.

Referee #2 (Remarks for Author):

This manuscript discovers that a family of angiogenic factors, Angiopoietin-2 and its receptor Tie-2, never previously studied in non-functioning pituitary adenomas (NFPA), is expressed in NFPA, and has a relevant pro-tumourigenic role. Experiments in vivo included in this work led to the suggestion that therapy could be used as a new therapeutic tool for aggressive NFPA resistant to therapies or for those NFPA in older patients with comorbidities that cannot otherwise be treated with surgery and where radiotherapy is too risky.
The knowledge is completely new and I think it merits its publication in EMM.
However, I have two main concerns that should be addressed before final publication:
1) Nomenclature
2) Models
Since, in a way both are related I will address both together.
The nomenclature of PitNets for Pituitary Adenomas started in the last 3 years in an attempt to reclassification of all endocrine tumours together by the pathology committee in charge of the WHO tumor classifications. They wanted to include the pituitary in the same group as all the other neuroendocrine tumours. However, not everybody agrees with this name. In other organs with multiple epithelial populations, it has sense to point out that neuroendocrine tumours come from this tiny endocrine population within the whole organ, and called them NET. However, in the pituitary all the gland is composed of endocrine cells secreting

different hormones. Thus, the importance for pituitary tumours is if they secrete or not, and which hormone (if), and not if they are neuroendocrine or not, because they are.
NFPA is a tumour non-secreting any hormone. Due to this, the clinical follow-up is hard since there is not marker of therapy success. Only image studies (MR) indicate its course.
I understand that the group doing this work, is a firm believer in the proposed "recent" nomenclature. However, the disease NFPA is endocrine, it appears with this name in all databases and many previous publications and public patients' information websites. At least, once in the manuscript should be used as a synonym, in order to reach all medical specialities implicated and patient's associations.
And here comes my main claim: the term PitNet is equivalent to "pituitary adenoma". But the authors use it throughout the manuscript PitNet exclusively as NFPA sometimes, while other times as the whole group of pituitary tumours (Somatotropinoma, Prolactinoma, Corticotropinoma, Gonadotropinoma, and NFPA). While at the beginning (Abstract, Introduction) the focus is in aggressive NFPA, throughout the manuscript they change the focus and repeat constantly PitNet, as if they wanted to include all kind of pituitary adenomas. This is very confusing, and makes a chaotic comprehension.
To this we must add the confusion of "models". The GH3 cell line was obtained in the last century as a "GH /PRL secreting pituitary adenoma cell line", together with GH1, and GH4C1. Some of this lines secret more GH and some more PRL, but all three are taken as a model of secreting tumour.
Experiments in this manuscript use two kind of rodent models: i) Pituitary tumours from MENX rats. This the authors indicate are a good in vivo model of NFPA. ii) GH3 cell line with different inhibition or treatments. This second model does not fit at all with the main focus of the manuscript that is to study the role of Angiopoietin-2/Tie-2 in NFPA.
The experiments in GH3 lead to some confusion. Although from the mechanistic point of view they are useful, the insistence of using it in animal models changes the focus of the manuscript, since they are somatotroph cells, and the authors indicate in Supp Figure 2 that normal somatotroph (GH) cells express Angiopoietin-2. Thus, all those experiments may be are telling another story of the role of this Ang-2/Tie-2 system for normal somatotrophs.
Thus, my recommendation is to put all GH3 experiments together in one figure (and Supplementary as needed), but move the focus in the manuscript to the nice data on human and rat NFPA tumours, while deepening its characterization.
The authors have a perfect model in the MEN-X pituitary tumours, a model that includes all immune system and that is much better than a xenograft. And they did not characterize well the Angiopoietin-2/Tie-2 in those MEN-X tumours. NFPA tumours express SF-1, GATA-3 and FSH beta subunit. No attempt to co-stain any of those markers with Angiopoietin-2 / Tie-2 in the initial description in MENX, or with Ki-67 / Annexin -V in the last experiments is observed.
I think, a better characterization of the MENX model, expanding the figures dedicated to this, should be a must in the manuscript.
Moreover, in the in vivo experiments of Figure 7 in MEN-X (f-k sections) ADC is used as an indication of less cellular density. I understand that reduction in tumour size is very difficult to see due to the poor quality of MR in rat heads. However, for those inoperable NFPA in older patients it should be enough if the tumour did not grow with a pharmacological therapy. Could it be measured the tumours from day 0 and day 14 and see if the "placebo" animals have a growing curve while the AMG386 treated animals do not?
Could they use sectioning/reconstruction to calculate MEN-X tumour volumes in those experiments?
Finally, regarding the human data. A nice correlation between Ki-67 and Angiopoietin-2 is established in the Figure 1. This would fit with the "classical" classification of pituitary tumours where ki-67 >3% indicated aggressiveness. However, in the 2018 PitNet classification, it is the combination of Ki-67 and macroscopic invasiveness (as observed by the surgeon, not MR/Knosp) what gives the indication of aggressiveness. In Table S1 both characteristics come together for a subset of tumours used in qRT-PCR. But I could not see the combination of invasion+Ki-67 correlated to Angiopoietin-2/Tie-2 RNA or protein expression.

Referee #3 (Remarks for Author):

In this manuscript, Pellegata and co-workers identify paracrine/autocrine ANGPT2-TIE2 signaling in cancer cells of rat and human pituitary tumors (PitNET). The study provides compelling evidence that cancer cells express both ANGPT2 and TIE2, and that cancer-cell TIE2 can be phosphorylated by ANGPT2. It seems that ANGPT2 supports survival of cancer cells that express TIE2. Genetic or pharmacological interception of the ANGPT2-TIE2 axis has some anti-tumoral activity in mice and rats carrying subcutaneous and autochthonous PitNETs, respectively.

The data are compelling and the conclusions are largely supported by the data presented. I only have a few suggestions and comments.

Suggested experimental revisions

- There is no convincing evidence that Tie2 is phosphorylated in the cancer cells of pituitary tumors (Fig S5). The image in Fig S5a shows clear phosphorylation in vascular cells, but only mild and scarce staining in non-vascular cells. This dataset should be improved by inclusion of additional data (human and rat tumors; quantification of the data in cancer cells versus vascular cells, etc.), and the results should be presented in the main figures, being a key message of the study.

- The data in Figure 7 show modest anti-tumoral activity of AMG386 in the autochthonous PitNET model. In order to strengthen the translational scope of the study, the authors may consider combining AMG386 with standard of care (eg, radiation).

- Quantification of the Proximity Ligation Assay (PLA) data is not shown in Figure 3 for panels 3f-g.

Text revisions

- Please adhere to formal gene/protein nomenclature. Gene or protein names do not have dashes, so it should be Tie2 (Italic) for the mouse/rat gene, TIE2 (Italic) for the human gene, and TIE2 for protein (both rat or human). Same for Ang2/ANG2, which should actually be Angpt2/ANGPT2.

- AMG386 is a peptibody that blocks both ANGPT1 and 2. Experiments were not conducted with specific ANGPT2 inhibitors. The authors should revise the discussion to empahasize this limitation. Obviously, it would be very informative if the authors could test specific ANGPT2 blockade (e.g., using LC06) in the Tie2-KO model, but I appreciate that it can be difficult to obtain the antibody and merge the results with the AMG386 data.

- The authors should clarify that the data in Figure 7 (autochthonous PitNET model) cannot distinguish effects of the peptibody on cancer cells versus other cellular compartments that express TIE2 and, therefore, are sensitive to ANGPT blockade. These include endothelial cells, pericytes and perivascular macrophages.

- Line 357, Figure 5. Please mention in the text the site of tumor injection.

- The rat PitNET model should be described as "autochthonous" rather than "endogenous".

- Please remove priority claims (e.g., "this is the firs time that we...")

REBUTTAL LETTER

We thank the Reviewers for their fair and constructive comments and for the opportunity they gave us to improve our manuscript.

We have endeavoured to address all the issues raised by the Reviewers and we here below reply point-by-point to their questions. The manuscript was modified following their suggestions, and changes/additions are in red in the revised manuscript for easy reference.

The format of the figures has been modified according to the reviewers' comments, and to accommodate the newly generated data. The gene/protein nomenclature has been changed throughout the text and in the figures according to the suggestion given by Reviewer 3 (ANG-1 → ANGPT1 etc.).

We realized that the quality of several images in the merged PDF that the Reviewers evaluated was sub-optimal (especially the immunofluorescences). We now provide higher quality pictures that should hopefully better convey the message. We kindly ask the Editor to allow the Reviewer access to the individual higher-quality figures.

According to Reviewer 1 (Points 5-7, 10-12), our original version contained data that was not absolutely essential, and figures that were too "busy". This made the article cumbersome to read and the figures difficult to interpret. Therefore, to afford a better understanding of the important results, we streamlined the text, we simplified the figures and several ancillary data have now been moved to Suppl. Material or eliminated, as suggested.

Reviewer 2 proposed that we concentrate on our MENX rat model and on primary cells instead of on GH3 cells. As we discuss in detail below, although GH3 cells are the only in vitro pituitary tumor model suitable to study Ang/Tie2 signaling, primary tumor cells/tissues and MENX rats themselves are certainly more relevant models of non-functioning pituitary tumors. Therefore, according to this Reviewer, we have condensed the data on GH3 cells, and expanded those concerning primary tumor cells/MENX rats.
Additionally, whenever possible, we have conducted the new experiments suggested by the all three Reviewers (e.g flow cytometry data and PLA), using primary tumors/cells from MENX rats. We hope that the Editor and the Reviewers will appreciate our efforts in using primary cells, given that they are technically challenging and their yield is limited. The need to use fresh rat tumor tissues for primary cell work has delayed the re-submission because we are limited in the number of homozygous mutant rats at the right age (8-9 months old) due to limited hosting capacity.

The revised version of the article has now 10 main Figures, 17 Suppl. Figures and 5 Suppl. Tables.

Point-to-point rebuttal to the reviewers' comments.

Referee #1:

Major comments:

1. Reviewer: *From the data shown it appears that Tie2 expression is heterogeneous in the tumors. Flow cytometry and comparison with Tie2 expression in ECs could resolve this issue and give an idea of the relative expression of Tie2 in tumor cells vs ECs. –*

After tumor treatment with the inhibitors, do the authors see treatment-escaped Tie2 or Ang2 negative cells?

Response: Following the Reviewers' comment, we have endeavored to setup the flow cytometry protocol. Given the suggestion of Reviewer 2 to concentrate on the rat tumors instead of the cell lines, we conducted flow cytometry using rat primary pituitary cells. We selected an anti-Tie2 antibody cross-reacting with the rat protein (CD202B, as reported in Mat&Meth). We also used an anti-CD31 antibody to detect the control endothelial cells (ECs), as requested by this Reviewer. To address the questions asked by this Reviewer, we used a protocol that would only detect surface proteins.

Preliminary feasibility experiments showed that from the rat pituitaries collected with the slow-freezing method that we usually employ, primary cultures of tumor cells could be established, whereas most of the ECs did not survive the procedure. Therefore, we sacrificed tumor-bearing MENX rats (n=7), and "freshly" isolated their primary pituitary cells for flow cytometry. These experiments showed that rat pituitaries contain Tie2+/CD31- cells (=tumor cells) and Tie2+/CD31+ cells (control ECs). Following gating for Tie2 expression, the percentage of these 2 cell populations is: Tie2+/CD31- cells = average 71.8%; Tie2+/CD31+ cells = average 23.7%. These data are now reported in the revised Figure 5a,b.
As requested by this Reviewer, we assessed the levels of the Tie2 fluorescence in these 2 populations, and found this level to be mostly overlapping (as shown in Figure 5c).

Concerning the second question, we assume that the Reviewer is referring to the treatment of tumor-bearing MENX rats with AMG386 in vivo, given that "tumor" is mentioned and not "cells". The response of the rats to the drug was evaluated by imaging during treatment, and by ex vivo analyses on tissues collected at the end of treatment. Given that we only have the post-treatment tissues we unfortunately cannot answer this question, as tumor cells in different rats express different levels of Angpt2 or Tie2. We will consider answering this question in a follow-up study, as another animal protocol approval by our local government will be necessary to perform the respective experiments.

2. Reviewer: *AMG386 inhibits both ANG1 and ANG2 and the tyrosine kinase inhibitor very likely also inhibits both Tie2 and Tie1. What is the evidence that the inhibition in vivo is Ang2 specific?*

Response: We thank the reviewer for raising this point. We indeed cannot claim that the inhibition of tumor growth in MENX rats treated with AMG386 is specific for Angpt2. While the stainings we performed on rat primary tumors demonstrated that they primarily express Angpt2 but very little Angpt1, in the tumor microenvironment there are ECs that are known to secrete both Angpt2 and Angpt1. We have clarified this point throughout the text (please see pages 9,12,15,19,20).

3. Reviewer: *At least in some of the signal transduction studies, it would be good to use Ang1 as control ligand for Tie2. - Is Tie1 or VE-PTP expressed in PitNET cells?*

Response: Originally, we focused our studies on Angpt2 because this is the angiopoietin that is most highly expressed by PitNET tumors and cells. However, considering the reviewer's comment we tested whether the Tie2 receptor on PitNET cells could be activated also by ANGPT1, its canonical ligand. We can now show that the Tie2 receptor on GH3 cells is phosphorylated in response to incubation with rhANGPT1 (Suppl. Figure S8).

Following the Reviewer's suggestion, we have included rhANGPT1 in the treatment of isolated rat primary tumor and endothelial cells, followed by western blotting to assess activation of Tie2 (Figure S11) (see also Point 4 below).

Although the characterization of the role of Tie1 and VE-PTP in PitNET cells is beyond the scope of our study, following the reviewer's comment we assessed the expression of Tie1 and VE-PTP in rat primary PitNET cells. The results are reported in the Figure here below intended for the Reviewers only. They show that these two genes are expressed in the tumors, with Tie1 being expressed at much lower levels in tumor cells versus normal rat pituitary cells.

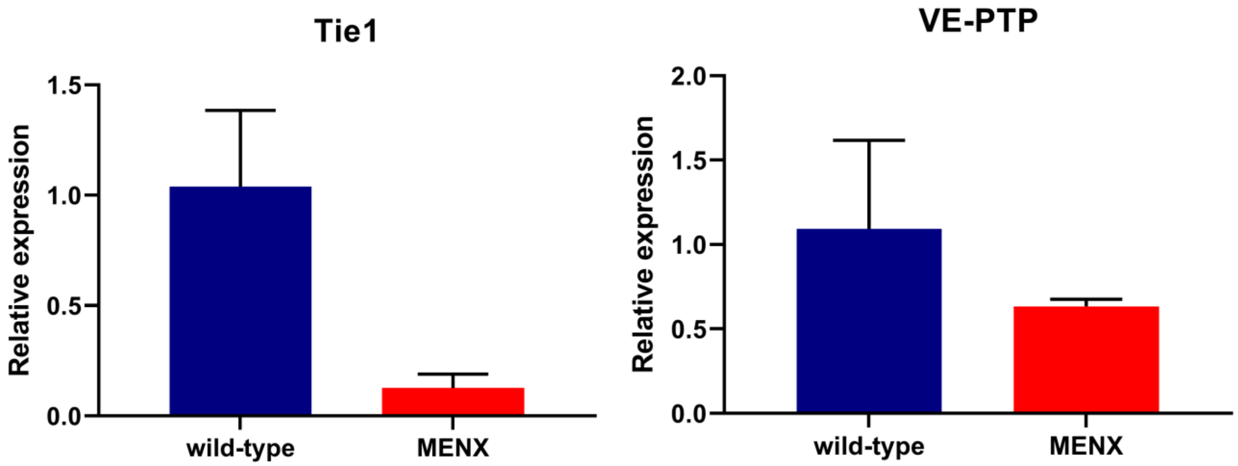

Figure A. Expression of Tie1 and Ptprb (encoding VE-PTP) genes in wild-type rat pituitaries versus MENX-associated pituitary tumors. RNA was extracted from 3 age-matched rats for each group. qRT-PCR was performed using TaqMan assays specific for the rat genes and normalized against the average of the wild-type rats.

4. Reviewer: *What exactly are the cells expressing Ang2 in the normal pituitary? Are they the origin of PitNETs? Are there Ang2 plus Ang1 positive cells and if so, what are their ratios and are there any coexpressing cells?*

Response: We thank the Reviewer for this question that allows us to clarify the issue of the Angpt2-positive cells. The cells expressing Angpt2 in human normal pituitary are somatotroph given that they are positive for GH, as demonstrated by co-IF with ANGPT2 and GH. The ANGPT2 positive cells do not express alpha-subunit, a marker of gonadotroph cells, which are the cell of origin of NF-PitNETs in both patients and MENX rats [PMID: 22524684].

In human NF-PitNETs, ANGPT1 is expressed at much lower level than ANGPT2 (see Figure 1) but sparse cells co-express both proteins, as illustrated in Suppl. Figure S2e.

5. Reviewer: *It seems that this manuscript has been submitted previously to another journal, and the authors have not had the energy to streamline their report before resubmission. The*

manuscript text should be shortened considerably, for example, the abstract is about twice the 175 word limit. The figure legends are much too long! They repeat material that should be presented in the materials and methods. The writing should be edited into a fluent text, crystallizing only the essential findings, which would be easier for the readers to digest. (The authors could perhaps use help from a professional editor).

Response: According to the reviewer's suggestion, we shortened the Abstract and reformatted the figures by enlarging the individual panels, providing higher resolution images and removing unessential data, or moving it to the Suppl. Material. The figure legends have been shortened.

6. Reviewer: *The figures are congested, forcing the miniaturization of many of the immunohistochemical panels, which are of variable quality. The authors should prune the material and transfer some of it to the Suppl. figures, concentrating only on the major and clear findings in those figures that should appear in print.*

Response: As also indicated in the Response to Point 5, we have endeavored to provide higher quality IHC and IF pictures. We have also deleted some data that was not absolutely critical to our story, and moved some figures to Suppl. Material.

7. Reviewer: *Figure 1 has miniaturized texts below the x-axis in panel c; and use much larger text in panel a, for example. In panel i, it is very difficult to find out where the mentioned vessel is located and the CD31 color code is misleading. A general recommendation is not to vary text size by more than a few points and have everything legible in the final reproduction-sized figure.*

Response: See also Response to Point 5. We have now reformatted Figure 2 and have enlarged the panels. Moreover, we increased the font size to make the text readable.

8. Reviewer: *In Figure 2b, it is impossible to say whether the Tie2 receptors are on the cell surface or internalized into some kind of vesicles near the plasma membrane. Flow cytometry of cell surface-stained control and tumor cells should be shown.*

Response: We thank the Reviewer for raising this issue. For our immunofluorescence (IF) experiments, we used an anti-Tie2 antibody raised against the N-terminal extracellular domain (ECD) of the receptor [PMID: 16849318]. This information has now been included in the text for clarification.

To address this point, and as suggested by this Reviewer (see also Point 1), we have conducted flow cytometry of rat primary PitNETs. We employed a protocol to detect only surface proteins. Upon gating for Tie2+, a sub-population of these cells was CD31+ (= control ECs) but the majority of these cells were CD31- (= tumor cells).These results suggest that indeed the tumor cells expose Tie2 on their membrane. This data is now reported in revised Figure 5a-d.

9**. **Reviewer: *Figure 3e could be moved to the end of the discussion. The few PLA signals in Figure f could be outside of the cells, and some may be in the cell-free area. A more convincing presentation should be shown with quantification of the signals.*

Response: In agreement with this remark, the graphical abstract depicting the angiopoietin/Tie2 signaling in PitNETs (old Figure 3e) is now the last figure of the manuscript (Figure 10) and is presented in the Discussion (Page 18).

We thank the Reviewer for the remark on the PLA data. Following this comment, we have repeated the PLA assay on additional rat NF-S8PitNET tissues and we now show better quality pictures. Additionally, as requested by this Reviewer (see Point 21), we now include PLA for Angpt2 and Tie2 done on control RAOECs to validate the antibodies and the procedure (revised Figure S8).
The number of positive interactions between Ang2 and Tie2 (=dots) in tumors was quantified and is now shown in Figure 5.

10. Reviewer: *Figure 4 panels g and h are not very convincing. For example, P-FAK signal is increased at 30 min, but how reproducible is this? Some of the data here is not critical for the main conclusions of the manuscript. Again, I would like to suggest that the authors prune some of the material in panels e-h out altogether or transfer it to the supplement.*

Response: We agree with the Reviewer that the indicated panels are not critical for our story and therefore we have now deleted them, as suggested.

11. Reviewer: *Because of the poor resolution of the very small text in panel a of Figure 5, a and b could be also moved to the supplement.*

Response: As suggested, we have moved panel **a** of the old Figure 5 to Suppl. Figure S7, and we have enlarged panel b (which is now panel **a** in revised Figure 5) as it helps interpreting the results reported in the other panels of the figure.

12. Reviewer: *The layout should be also improved in Figure 7. For example, panel d could be moved to the supplement, the schematics showing rodent injection could be removed from the figure, thus allowing expansion of panel a, where the text is too small.*

Response: We have now moved the schemes of the in vivo AMG386 treatments of the rats, and the ex vivo data on the short-term rat treatment to Suppl. Figure S9, and enlarged the remaining panels in revised Figure 7.

13. Reviewer: *Panel F in Suppl. Figure 2 is of unacceptable quality. Please show staining of EC islands or confluent EC layers. Panel d in the same figure is too small for the resolution of cellular detail. The text does not specify what alpha subunit is.*

Response: We thank the reviewer for this remark which allowed us to improve our data. Given that we extensively worked with rat tumor cells/cell lines, we decided to include in our study rat aortic endothelial cells (RAOECs) as additional control. These cells grow at higher density than HUVECs in our hands. Therefore, we used these cells to repeat the IF for Angpt2 and Tie2, as well as CD31 (control marker of ECs). The results are illustrated in Suppl. Figure S3b,c and show co-expression of Angpt2 and Tie2 in ECs.

We apologize for this oversight. We have now added a sentence explaining that the alpha-subunit of the gonadotropin hormones is a marker of pituitary gonadotroph cells, the cell of origin of NF-PitNETs in both patients and MENX rats (PMID: 22524684).

14. Reviewer: *The panel B in Suppl. figure 5 is supposed to show "Ang-2 (magenta), Tie-2 (green) and P-Tie-2 (red)", but the staining for Ang-2 and Tie-2 are not shown. The immunofluorescent staining in panel C of Suppl. figure 5 is impossible to see. The Tie-2 phosphorylation should be shown by western blotting.*

Response: We apologize for this oversight, the text of the figure legend was indeed incorrect.

We agree with the Reviewer that it is also important to show Tie2 phosphorylation by western blotting (WB). This is why we show the activation of Tie2 upon GH3 cell stimulation using the recombinant ANGPT2 at different doses or at different time points (Figure 6e,f).

We have also performed WB using extracts of isolated rat primary tumor and endothelial cells (following the suggestion of Reviewer 2 to concentrate on primary tumor cells). These cells were serum-starved and then stimulated with rhANGPT1 and rhANGPT2. As positive control, we included RAEOCs stimulated with rhANGPT1 or left untreated (Figure S11). Despite the technical challenges posed by isolated primary tumor and endothelial cells, we could observe an increase in P-Tie2 in both cell populations upon treatment with the ligands (Figure 6h).

15. Reviewer: *Suppl. figure S8 lacks a negative control.*

Response: We have now included the negative control of the TaqMan in the figure.

16. Reviewer: *In Table S5, the negative values lack an explanation.*

Response: We thank the reviewer for noticing the missing information. An explanation has now been added to the Materials and Methods paragraph on ELISA assays, which reads as follows:

..” To each measurement, the value of the serum-free medium alone (blank) was subtracted. The values of the supernatants of shAngpt2-infected cells were close to/lower than those of the blank, indicating no secretion of Angpt2.”

17. Reviewer: *Statistical analysis. P-values for Figure 2J and 4B are given by t-tests although there are 4 groups in both figures. It is not clear how the statistical analysis was done for Figures 6G and 6I.*

Response: We thank the reviewer for pinpointing this oversight. In the legend of the original Figure 2J there was an error. Indeed, it was first indicated that the statistical analysis was done by one-way ANOVA, and then later it was indicated by t-test. We apologize for this mistake. The first information was correct: the statistical analysis was done by one-way ANOVA. The text has now been amended.

In the original Figure 4B, comparisons were made between two conditions (shCtrl-transfected cells with versus without rhANG2; shAng2-transfected cells with versus without rhANG2; shCtrl-

transfected versus shAng2-transfected cells without rhANG2). For these comparisons, t-test was used as indicated.

Figures 6G and 6I show the response of the human primary cultures to AMG386 or Tie2-KI, respectively. The average cell viability value of the treated primary cultures was normalized against the average value of the untreated cultures for each patient, set to 1 to simplify the figure. This is why there is only 1 bar representing the control. Each patient’s culture was normalized against its own control and the statistical analysis was done by t-test. To clarify this issue, we describe the experimental approach of the treatments in more details in the Suppl. Materials and Methods.

18. Reviewer: *In Figure 1, the authors show that human PitNETs express both ANG-1 and ANG-2, but on page 12 the authors justify focusing on the interaction between Tie-2 and Ang-2 by saying that "PitNET cells express and secrete a bioactive Ang-2, but do not express Ang-1 ....". This is not correct. The putative role of Ang-1 should be considered and discussed especially since AMG-386 inhibits both Ang-1 and Ang-2 signaling.*

Response: We thank the reviewer for raising this point. We show that primary rat and human tumors express low levels of ANGPT1, lower than normal pituitary, whereas in GH3 cells we could not detect Angpt1 expression by western blotting. Anyway, we agree with the Reviewer that we cannot state that PitNET cells do not express Angpt1. We have now clarified this point throughout the article.

Also, the effect of AMG386 in vivo is not specifically mediated by the inhibition of Angpt2, given that in the tumor microenvironment there are ECs that secrete both Angpt1 and Angpt2. We have now clarified the text, and modified Figure 10 accordingly.

We originally focused on Angpt2 because in PitNET cells this cytokine was expressed at much higher levels than Angpt1. Moreover, it is known that ANGPT2 is highly expressed in the angiogenic tumor-associated vasculature [PMID:21576085; 33217955], which then promotes angiogenesis in te hpresence of VEGF. It has been reported that human NF-PitNET cells express and secrete VEGF [PMID:17395978; 22350942], and so by also secreting Angpt2 there was a parallel with ECs.

19. Reviewer: *The authors discuss cytoplasmic and membrane-bound protein expression in several places, but the low resolution of the IF images does not support such conclusions.*

Response: We thank the Reviewer for this comment. In the original version of the manuscript, due to the space limitation and to the incorporation of a lot of data, the pictures of the immunofluorescences (IF) were probably too small to really appreciate the staining pattern. Moreover, the quality of the images in the final merged PDF was rather low.

Co-staining with the membrane marker Na+K+ATPase showed that Tie2 co-localizes with this pump, thereby indicating that it localizes to the membrane. Most importantly, as suggested by this Reviewer, we performed flow cytometry experiments using rat primary pituitary cells (see also Point 1). We selected a protocol that only allowed to detect surface proteins. As shown in the revised Figure 5a-d, gating for Tie2+ cells demonstrated that the rat tumors include a Tie2+ cell population which is negative for the EC marker CD31 (=tumor cells).

Additionally, also the PLA results on the isolated NF-PitNET cells show that Tie2 is localized on the membrane (please see answer to Point 21). Therefore, we are confident that NF-PitNET cells expose Tie2 on their membrane.

20. Reviewer*: In the Suppl. figure 3A, the authors show Ang-1 and Ang-2 expression in GH3 and HUVEC cells. Obviously, the authors show cell lysates and not the supernatants. HUVECs show two bands for Ang-2: the premature 55 kDa form and the mature (to be secreted) form of about 70 kDa. GH3 cells do not have the mature form. Please, show the Ang-2 secretion from GH3 cell by analyzing the supernatants.*

Response: Thanks to the Reviewer's remark we now provide more complete information on Angpt2 secretion. Indeed, we previously only measured the secretion of Angpt2 by GH3, HUVECs or primary cells by ELISA. Following this suggestion, we have also analyzed the supernatant of GH3 and HUVEC cells for the secretion of Angot2 by western blotting.

We used as positive control a GH3-derived clonal population overexpressing ANG2 (GH3-ANG2OE cells). Similar to control HUVECs, both GH3 parental and the GH3-ANG2OE cells secrete the mature form of Angpt2, which is glycosylated, as demonstrated by the digestion with the PNGase enzyme (Suppl. Figure S6e,f). This data is now discussed in page 11.

21. Reviewer*: For proximity-ligation assay (PLA, Figure 3f, g) the authors should use a positive control, for example, cultured endothelial cells. This would show how much Ang2/Tie2 interaction (intra/extracellular?) occurs in cells with known expression of Tie2. The current panels show very few dots. Every dot should represent a single interaction. Does the result mean that the cells express very little or no Tie2?*

Response: Based on the Reviewer's suggestions (see also Point 9), we repeated PLA assays on cultivated rat aortic ECs (RAOECs) as positive control.

RAOECs were grown on coverslips, fixed and PLA was conducted using the two antibodies (against Angpt2 and Tie2) or the single antibodies (negative control). The results show that there are many interactions between Angpt2 and Tie2, occurring mainly at the membrane of the cells (Suppl. Figure S8b).

As the Reviewer correctly points out, PLA assays reveal the interaction between Angpt2 and Tie2. The aim of our PLA studies was to demonstrate that Tie2 on tumor cells can bind to Angpt2. This was meant as a "qualitative" assay and not as a quantitative one.

As also indicated in the Response to Point 9, it is important to consider that our initial PLA studies were conducted on sections of primary FFPE rat pituitary tissues. We speculate that the low number of interactions present in rat NF-PitNET tissues might be due to the experimental setting more than to a low number of Tie2 receptor molecules present on the tumor cells. Considering that the PLA was performed on tissue sections (=one layer), that the processing of the tissue could impact on the interaction between Angpt2 and Tie2, and that the amount of Angpt2 present in the tumor microenvironment (TME) is likely variable, we believe that we cannot state that there are few molecules of the receptor on the tumor cells, but only that with this approach we can visualize only part of the possible interactions between ligand and receptor. Again the flow cytometry data conducted upon the suggestion of this Reviewer confirmed the presence of Tie2 on the tumor cell membrane

Nevertheless, we understand the Reviewer's point: the presence of positive dots does not necessarily mean that the Tie2 receptor is exposed on the membrane of the tumor cells. To prove this, we followed a different approach.

Following the request of Reviewer 2, we concentrated our efforts on primary NF-PitNET cells. We eliminated the primary endothelial cells (using CD31-coated magnetic beads) from the whole pituitaries of two 9-month-old rats having extremely enlarged glands, indicating the presence of tumors effacing and replacing the gland, as previously reported [PMID: 22524684].
Upon serum-starvation, the CD31-negative primary tumor cells were incubated for 15 min with rhANGPT2 or vehicle, and then fixed and processed for PLA using the Angpt2 + Tie2 antibodies, or the single one as negative controls. We then quantified the number of interactions. We chose to count the dots instead of assessing the overall fluorescence because we believe it is more informative to know the number of dots/cell than a general positivity.

Angpt2 is not internalized with Tie2 upon binding. As illustrated in Figure 5c, the incubation with rhANGPT2 increased dramatically the number of tumor cells showing an interaction between Angpt2 and Tie2 (from 17.6% to 32.2%), but also, and more interestingly, the treatment increased the number of interactions per cell, which are distributed around the cells. Indeed, while 97% of the positive tumor cells in the absence of rhANGPT2 had between 1 and 3 dots, almost 50% of the cells incubated with rhANGPT2 had over 3 dots/cell, with some cells remarkably showing >10 dots/cell (Figure 5d). Primary NF-PitNET cells aggregate in clusters and therefore the pictures taken give an idea about the 3D distribution of the interactions (Suppl. Figure S8a).

These data suggest that indeed the Tie2 receptor is located at the membrane of the tumor cells and can interact with Angpt2. The amount and availability of the ligand is of key importance in order to detect interactions with Tie2 by PLA. This is something that in tumor tissues ex vivo cannot be estimated (as discussed above).

We sincerely hope that these data addresses the Reviewer's concerns.

22. Reviewer: *These PLA data should be co-stained for EC markers to show how much the Ang2/Tie2 interaction occurs on tumor cells vs. ECs of blood capillaries.*

Response: As also discussed in Points 9 and 21, for our PLA studies we used rats at >8-months of age, with large tumors replacing most of the gland, as confirmed by histology. PLA was performed on primary tumor tissues to offer additional evidence that Tie2 in tumor cells is able to interact with Angpt2. The pictures we show in Figure 5a were taken in the tumor areas.

Studies done on PitNET cells upon stimulation with rhANGPT2 (and rhANGPT1, upon this Reviewer's suggestion) showed that the receptor on these cells can indeed be activated and therefore it is functional. The role of Tie2 in these tumors is further supported by the in vivo studies engrafting cells with/without Tie2, with the latter growing more slowly in mice.

The high expression of Angpt2, Tie2 and P-Tie2 in the rat NF-PitNETs is supported by original and newly performed immunofluorescent stainings (Figures 2, S4). The flow cytometry data, performed following this Reviewer's suggestion, clearly show that rat NF-PitNET cells have an expression of Tie2 which mostly overlaps that of the ECs (Figure 4e,f). Based on the data illustrated in Figures 5c and S8, we know that Tie2 is indeed present on the membrane of primary

NF-PitNET cells and when the ligand is available then the interactions detected by the PLA assay increase.

In our PLA studies on rat tumor tissues, we of course also saw interactions between Angpt2 and Tie2 in tumor-associated ECs (an example is shown in Figure A below).

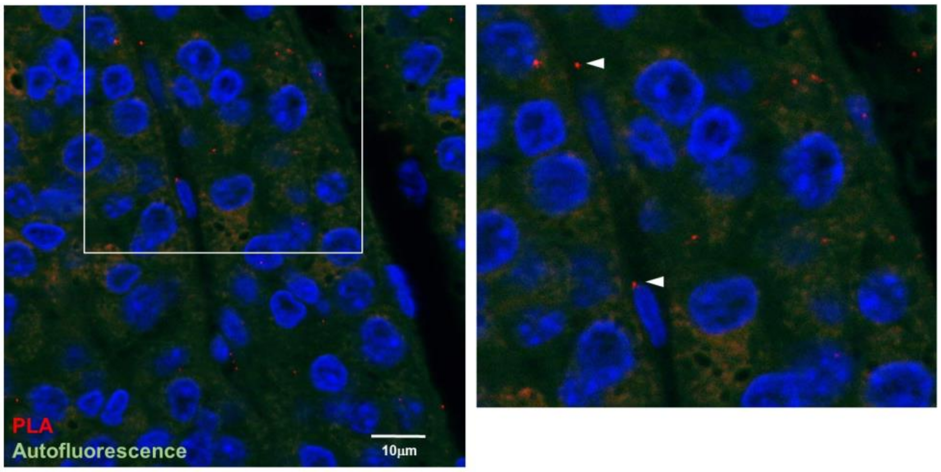

Figure A for Reviewer 1.

Unfortunately, the CD31 antibody that we optimized for the staining of rat cells could not be combined with the PLA (same species).

It should be kept in mind that the pituitary tumors of MENX rats are multifocal (as is the case in tumors of human patients affected by the MEN1 syndrome) and heterogeneous in terms of stage of progression and vessel density, as shown in the Figure B below (unpublished data). This heterogeneity reflects the situation in patients, and can only be recapitulated in an endogenous model, hence our interest on this particular animal model.

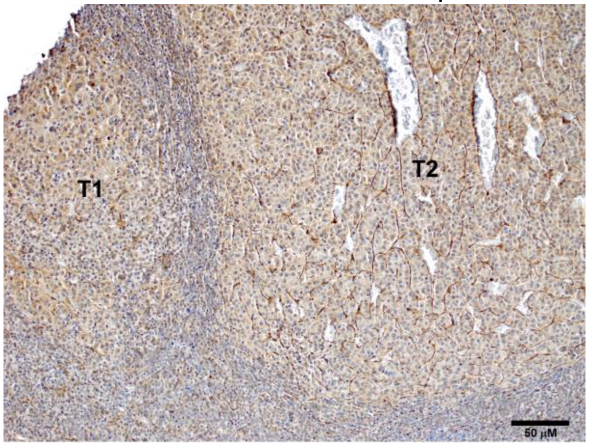

Figure B for Reviewer 1. CD31 immunohistochemistry of the pituitary gland of a 7-month old MENX rat having multifocal tumors with different vessel density (T1, T2).

Due to the variable vessel density in the various tumor areas, and to likely fluctuation of Angpt2 levels in the TME (that we cannot estimate), the number of interactions between Angpt2 and Tie2 in tumor-associated ECs is bound to be different in the different tumor areas. Based on the experiments on the isolated primary cells (see also Point 21 and Figure 5c, S8a), the availability of Angpt2 is of key importance for PLA positivity. Additionally, due to the limitations of applying PLA to FFPE tissue sections (see also Point 21), we would be most likely underestimating the

interactions between Angpt2 and Tie2 on ECs. Therefore, we believe that, the estimates of the number of Angpt2-Tie2 interactions on ECs and tumor cells obtained using this approach might not reflect the situation in vivo. Our main intention with this experiment was to determine whether the Tie2 receptor on the tumor cells is able to interact with Angpt2 in a “qualitative” manner. The issue of assessing the number of receptor molecules on the tumor cells vs. ECs is of great interest, and we may consider to conduct appropriate experiments in a follow-up study.

In any case, the PLA data obtained upon incubation of primary tumor cells with rhANGPT2 clearly show that the tumor cells have Tie2 on their membrane, which can interact with Angpt2 (Point 21).

23. Reviewer: *In Figure 4, the authors show that Tie2 kinase inhibitor surprisingly activates ERK1/2 phosphorylation. The effect of Tie2 kinase inhibitor in GH3 cells should be compared to its effect on ECs.*

Response: In order to follow the suggestion of Reviewer 2, we focused our attention mainly to primary tumors cells during the revision, therefore we did not study the effects on the GH3 cells in more detail, although the comparison of the GH3 effect compared to ECs would have helped to understand the process in GH3 cells better. Additionally, upon the suggestion of this Reviewer, some of the data previously shown in Figure 4 (panels g and h showing the treatment with Tie2-kinase inhibitor) were eliminated as they were deemed to be not essential to our story.

24. Reviewer: *Why does Ang2 treatment cause pERK1/2 phosphorylation in Figs. 4e and f, but not in Figure 4h? Both represent the 30 min timepoint (panels e and h) and the concentrations are the same (panels f and h).*

Response: In the original version of the manuscript, Figure 4 panel e was generated by treating GH3 cells with 800ng/ml of rhANG2, whereas in panel h cells were treated with 300ng/ml of rhANG2. In the dose-response experiment shown in panel f (where we tested different concentrations of the recombinant protein), at the concentration of 300ng/ml of rhANG2 there was a very small effect on P-ERK1/2. So, this is the reason why in panel h there was not a detectable increase in P-ERK1/2.

We agree with the Reviewer that the original panels g and h were not critical to our story (see also Point 10), and therefore we have now eliminated them, as suggested.

25. Reviewer: *It seems that intracellular signaling in the H-PitNET cells differs from that in GH3 cells. For example, pERK1/2 is not increased by the TKI treatment (Figure 6k vs. 4g, h). Is it possible that Tie2 kinase regulate distinct processes (e.g. proliferation, survival) in the GH3 vs H-PitNET cells?*

Response: The Reviewer is correct in saying that, upon treatment with the Tie2-KI, the downstream pathways inhibited in GH3 cells differ from those in human primary cultures. GH3 cells do not express P-Akt, whereas the human tumors seem to express it at detectable levels. Moreover, if GH3 cells proliferate, the primary human cells do not divide within the course of the treatment. Therefore, it is highly plausible that this inhibitor elicits a different response in the two types of tumor cells.

26. Reviewer: *Does the endogenous Ang2 activate Tie2 intracellularly, in secretory vesicles? This could compromise the efficacy of the anti-Ang2 treatment at least to some degree.*

Response: We thank the Reviewer for this interesting question. While we can state that Tie2 is exposed on the membrane of tumor cells based on flow cytometry data and on the stimulation with recombinant ligands, we cannot say whether Ang2 and Tie2 also interact in secretory vesicles.

We have however proven that the treatment with anti-angiopoietin biologicals (AMG386) is highly effective against PitNETs in vivo. Upon re-analysis of the MRI data obtained on MENX rats treated with the anti-angiopoietin AMG386 peptibody (as suggested by Reviewer 2, Point 5), we now show that the inhibition of this pathway in vivo even for only 14 days already reduces tumor growth, although it did not reach statistical significance. This data is anyway highly remarkable as we did not observe changes in tumor volume in our rat model upon treatment with a PI3K/mTOR inhibitor [PMID: 25838390].

Referee #2 (Remarks for Author):

1. *Reviewer: The nomenclature of PitNets for Pituitary Adenomas started in the last 3 years in an attempt to reclassification of all endocrine tumours together by the pathology committee in charge of the WHO tumor classifications. They wanted to include the pituitary in the same group as all the other neuroendocrine tumours. However, not everybody agrees with this name. In other organs with multiple epithelial populations, it has sense to point out that neuroendocrine tumours come from this tiny endocrine population within the whole organ, and called them NET. However, in the pituitary all the gland is composed of endocrine cells secreting different hormones. Thus, the importance for pituitary tumours is if they secrete or not, and which hormone (if), and not if they are neuroendocrine or not, because they are.*
NFPA is a tumour non-secreting any hormone. Due to this, the clinical follow-up is hard since there is not marker of therapy success. Only image studies (MR) indicate its course.
I understand that the group doing this work, is a firm believer in the proposed "recent" nomenclature. However, the disease NFPA is endocrine, it appears with this name in all databases and many previous publications and public patients' information websites. At least, once in the manuscript should be used as a synonym, in order to reach all medical specialities implicated and patient's associations.

Response: We agree with the Reviewer. Thus, we have now included the classical nomenclature “NFPA” at the beginning of the Introduction as suggested.

2. ***Reviewer:*** *And here comes my main claim: the term PitNet is equivalent to "pituitary adenoma". But the authors use it throughout the manuscript PitNet exclusively as NFPA sometimes, while other times as the whole group of pituitary tumours (Somatotropinoma, Prolactinoma, Corticotropinoma, Gonadotropinoma, and NFPA). While at the beginning (Abstract, Introduction) the focus is in aggressive NFPA, throughout the manuscript they change the focus and repeat constantly PitNet, as if they wanted to include all kind of pituitary adenomas. This is very confusing, and makes a chaotic comprehension.*

Response: The Reviewer raises an important point, which is related to the following comment and therefore we answer to both at the same time below.

3. Reviewer: *To this we must add the confusion of "models". The GH3 cell line was obtained in the last century as a "GH /PRL secreting pituitary adenoma cell line", together with GH1, and GH4C1. Some of this lines secret more GH and some more PRL, but all three are taken as a model of secreting tumour.*
Experiments in this manuscript use two kind of rodent models: i) Pituitary tumours from MENX rats. This the authors indicate are a good in vivo model of NFPA. ii) GH3 cell line with different inhibition or treatments. This second model does not fit at all with the main focus of the manuscript that is to study the role of Angiopoietin-2/Tie-2 in NFPA.
The experiments in GH3 lead to some confusion. Although from the mechanistic point of view they are useful, the insistence of using it in animal models changes the focus of the manuscript, since they are somatotroph cells, and the authors indicate in Supp Figure 2 that normal somatotroph (GH) cells express Angiopoietin-2. Thus, all those experiments may be are telling another story of the role of this Ang-2/Tie-2 system for normal somatotrophs.

Response: We thank the reviewer for this comment, which allows us to clarify the important issue of the choice of our experimental models.

As the Reviewer certainly knows, there are currently no cell lines derived NF-PitNETs/NFPAs.

As correctly pointed out by the Reviewer, these cells derive from a growth hormone (GH)- and prolactin (PRL)-producing rat PitNET. We have used extensively GH3 cells for our studies [PMID: 24532476; 25416039; 32856736], so we were aware of their origin.

However, we tested all the available pituitary cell lines (including αT3 and LβT2) for the expression of Ang-2 and Tie-2, as shown in Figure 3a, and GH3 cells were the only ones expressing both proteins at detectable levels. Moreover, similar to our MENX rat model, GH3 cells do not express p27 [PMID: 9811339].

We believe that GH3 cells currently represent the most suitable PitNET cell line model to conduct studies on Ang-2/Tie-2 in vitro. Our studies, however, also included primary NF-PitNET cells as additional and more physiological in vitro model. As recommended by the Reviewer, we specifically concentrated on MENX-associated tumors when conducting the experiments for the revision.

Given that the GH3 cells do not derive from gonadotroph cells, we could not refer to these cells as “NF-PitNETs” and therefore we used the word “PitNETs”. We apologize if this generated some confusion, but this is the only nomenclature that is not misleading.

4. Reviewer: *Thus, my recommendation is to put all GH3 experiments together in one figure (and Suppl. as needed), but move the focus in the manuscript to the nice data on human and rat NFPA tumours, while deepening its characterization. The authors have a perfect model in the MEN-X pituitary tumours, a model that includes all immune system and that is much better than a xenograft. And they did not characterize well the Angiopoietin-2/Tie-2 in those MEN-X tumours. NFPA tumours express SF-1, GATA-3 and FSH beta subunit. No attempt to co-stain any of those markers with Angiopoietin-2 / Tie-2 in the initial description in MENX, or with Ki-67 / Annexin -V in the last experiments is observed.*

I think, a better characterization of the MENX model, expanding the figures dedicated to this, should be a must in the manuscript.

Response: We thank the Reviewer for appreciating the potential and the utility of MENX rats as representative model of NFPAs. Moreover, thanks to this remark we have now novel information about the expression of some key proteins in the rat tumors.

The rat pituitary tumors were previously characterized for the expression of pituitary hormones and relevant pituitary transcription factors [PMID: 22524684; 23756599].

To address the Reviewer's comment, we have performed additional immunohistochemical staining of MENX rat pituitaries with antibodies against alpha-subunit of gonadotropin hormones (αGSU), the transcription factor SF1, and Tie2. We could observe a co-expression of αGSU and Tie2, as well as a co-expression of SF1 and Tie2 in the tumors of MENX rat, as illustrated in Suppl. Figure 4s. This co-expression is limited to the tumors, as in the adjacent non-tumor areas αGSU- and SF1-positive gonadotroph cells do not express Tie2.

5. Reviewer: *Moreover, in the in vivo experiments of Figure 7 in MEN-X (f-k sections) ADC is used as an indication of less cellular density. I understand that reduction in tumour size is very difficult to see due to the poor quality of MR in rat heads. However, for those inoperable NFPA in older patients it should be enough if the tumour did not grow with a pharmacological therapy. Could it be measured the tumours from day 0 and day 14 and see if the "placebo" animals have a growing curve while the AMG386 treated animals do not? Could they use sectioning/reconstruction to calculate MEN-X tumour volumes in those experiments?*

Response: We again thank the Reviewer for this question, which allowed us to expand the results of our in vivo studies. Given that in a previous study we did not see differences in tumor volume upon treatment of MENX rats with a PI3K7mTOR inhibitor for 14 days [PMID: 25838390], we were not expecting to see differences in tumor volume treatment with AMG386 for 14 days. This is why we used functional imaging (=DW-MRI) to detect early changes indicative of therapy response.

Following the Reviewer's suggestion, we went back to the MRI data and analyzed the changes in tumor volume upon treatment with AMG386 or placebo.
We are happy to report that the Reviewer was indeed correct: there is a trend for a slower tumor growth in AMG386-treated rats versus placebo-treated rats. Although the trend is not statistically significant, it is nevertheless a remarkable result after only 14 days of treatment. Therefore, these data further strengthen our hypothesis that in vivo treatment with angiopoietin-sequestering drugs should be beneficial against these tumors. These new results are illustrated in revised Figure 9d.

6. Reviewer: *Finally, regarding the human data. A nice correlation between Ki-67 and Angiopoietin-2 is established in the Figure 1. This would fit with the "classical" classification of pituitary tumours where ki-67 >3% indicated aggressiveness. However, in the 2018 PitNet classification, it is the combination of Ki-67 and macroscopic invasiveness (as observed by the surgeon, not MR/Knosp) what gives the indication of aggressiveness. In Table S1 both characteristics come together for a subset of tumours used in qRT-PCR. But I could not see the combination of invasion+Ki-67 correlated to Angiopoietin-2/Tie-2 RNA or protein expression.*

Response: We agree with the Reviewer that invasion, as determined by the neurosurgeon, is an important parameter in determining the aggressive potential of a pituitary tumor. Therefore, following this remark, we have checked for possible correlations between the expression of ANGPT1 and ANGPT2 at mRNA level and invasion or Ki67, as shown in the tables here below intended for the reviewers only. Unfortunately, when looking at the mRNA level, there was no correlation with invasion or Ki67 labelling index. We could not conduct these analyses using the IHC data because not for all the samples we had the data about invasion.

Ang1:

	X vs. invasion	X vs. Ki67
Pearson r		
r	0,3949	0,001410
95% confidence interval	-0,1717 to 0,7651	-0,5296 to 0,5316
R squared	0,1559	1,989e-006
P value		
P (two-tailed)	0,1623	0,9962
P value summary	ns	ns
Significant? (alpha = 0.05)	No	No

Ang2:

	X vs. invasion	X vs. Ki67
Pearson r		
r	0,2973	0,2657
95% confidence interval	-0,2770 to 0,7151	-0,3083 to 0,6979
R squared	0,08838	0,07060
P value		
P (two-tailed)	0,3020	0,3585
P value summary	ns	ns
Significant? (alpha = 0.05)	No	No

<u>Referee #3:</u>

1. ***Reviewer:*** *There is no convincing evidence that Tie2 is phosphorylated in the cancer cells of pituitary tumors (Fig S5). The image in Fig S5a shows clear phosphorylation in vascular cells, but only mild and scarce staining in non-vascular cells. This dataset should be improved by inclusion of additional data (human and rat tumors; quantification of the data in cancer cells versus vascular cells, etc.*), and the results should be presented in the main figures, being a key message of the study.

Response: We thank the Reviewer for raising the important point of the activation of Tie2 in tumor cells. In the original version of the manuscript, we had provided western blotting analyses of GH3 cells +/- stimulation with rhANGPT2 for the expression of Tie2 and P-Tie2, the latter found to increase in a time- and dose-dependent manner (as previously seen for ECs) (revised Figure 6c,d).

We had also shown images of immunofluorescence (IF) done on rat pituitary glands treated with/without AMG386. Indeed, we had stained rats of each group for P-Tie2 (total n=5/group) given that a reduction of P-Tie2 was an indication that the drug had reached the pituitary tumors and was acting as expected. A comment concerning this point is reported on Pages 17 and 18.

However, we realized that the IF panels in the original Figures might have been too small to really appreciate the staining results. Therefore, we have now a bit “decongested” the figures and enlarged the panels. We also provide higher resolution pictures of GH3 cells (parental or with the knockdown of Angpt2) treated with rhANGPT2 or left untreated (Figure 6c,d).

Following the Reviewer’s comment, we have endeavored to provide **additional data** supporting the phosphorylation of Tie2 in PitNET cells. As suggested by Reviewer 2, we concentrated on the primary NF-PitNETs more than on the GH3 cell line. We have performed co-staining for Angpt2 and P-Tie2 of the rat primary NF-PitNETs. These results show that there is a co-localization of Angpt2 and P-Tie2 in the tumor cells (Figure 6e and Suppl. Figure 10). It can be appreciated that the co-expression of Angpt2 and P-Tie2 occurs in the tumor areas, whereas in the adjacent non-tumor areas Angpt2-positive cells do not express P-Tie2. The positive staining of the tumor-associated ECs with the P-Tie2 antibody (Suppl. Figure 10) attests to the specificity of the antibody.
We also provide higher resolution pictures of GH3 cells (parental or with the knockdown of Angpt2) treated with rhANGPT2 or left untreated (Figure 6a,b).

Moreover, we have performed western blotting analyses using isolated primary rat tumor and ECs for the expression of Tie2, P-Tie2 upon incubation with vehicle, rhANGPT1 or rhANGPT2 (Figure S11). Control RAOEC cells were also stimulated and analyzed in parallel. Despite the technical challenges posed by isolated primary tumor and endothelial cells, we could observe an increase in P-Tie2 in both cell populations upon treatment with the ligands.

2. Reviewer: *The data in Figure 7 show modest anti-tumoral activity of AMG386 in the autochthonous PitNET model. In order to strengthen the translational scope of the study, the authors may consider combining AMG386 with standard of care (e.g. radiation).*

Response: We thank the Reviewer for this suggestion. Although these studies would certainly be interesting, we currently don’t have the permission to irradiate live rats and there is no appropriate equipment to perform these experiments at the Helmholtz Zentrum München. Thus, we may address this issue in a subsequent study.

Of interest to the Reviewer, we have analyzed the anatomical MRI data (as suggested by Reviewer 2) and we found that AMG386 treatment does suppress tumor growth, although not significantly. We had not originally checked for this possibility as we were not expecting to see differences in tumor volume upon treatment for only 14 days. We only concentrated on functional imaging parameters (e.g. DW-MRI, as surrogate marker of tumor cellularity).

Indeed, in a previous in vivo study evaluating the effect of a dual PI3K/mTOR inhibitor against rat pituitary tumors, a 14-day treatment regime had elicited changes in functional parameters (DW-MRI) but not yet in tumor volume as determined by MRI [PMID: 25838390]. This was the reason why we assessed DW-MRI also in the current study to detect early changes indicative of therapy response. The finding that AMG386 reduces tumor growth after only 14 days of treatment is remarkable. These data further strengthen our hypothesis that in vivo treatment with angiopoietin-

sequestering drugs should be beneficial against these tumors. These new results are illustrated in revised Figure 9d.

3. Reviewer: *Quantification of the Proximity Ligation Assay (PLA) data is not shown in Figure 3 for panels 3f-g.*

Response: Following the Reviewer's remark, as well as comments from Reviewer 1, we have repeated PLA on rat NF-PitNET tissues and we quantified the data, now reported in revised Figure 5a,b.

Text revisions:

- Please adhere to formal gene/protein nomenclature. Gene or protein names do not have dashes, so it should be Tie2 (Italic) for the mouse/rat gene, TIE2 (Italic) for the human gene, and TIE2 for protein (both rat or human). Same for Ang2/ANG2, which should actually be Angpt2/ANGPT2.

Response: According to the Reviewer's comment, we have amended the gene/protein nomenclature.

- AMG386 is a peptibody that blocks both ANGPT1 and 2. Experiments were not conducted with specific ANGPT2 inhibitors. The authors should revise the discussion to empahasize this limitation. Obviously, it would be very informative if the authors could test specific ANGPT2 blockade (e.g., using LC06) in the Tie2-KO model, but I appreciate that it can be difficult to obtain the antibody and merge the results with the AMG386 data.

Response: We thank the Reviewer for this remark, which allowed us to improve our manuscript. We now emphasize the fact that AMG386 blocks both angiopoietins and therefore the effects observed in vivo cannot be solely ascribed to Angpt2 (Page 20).

- The authors should clarify that the data in Figure 7 (autochthonous PitNET model) cannot distinguish effects of the peptibody on cancer cells versus other cellular compartments that express TIE2 and, therefore, are sensitive to ANGPT blockade. These include endothelial cells, pericytes and perivascular macrophages.

Response: Following the Reviewers remark, we have now added a comment to clarify this issue in the Discussion (Page 20).

- Line 357, Figure 5. Please mention in the text the site of tumor injection.

Response: The site of the injection has been included, as suggested.

- The rat PitNET model should be described as "autochthonous" rather than "endogenous".

Response: We have amended the text according to the Reviewer's comment.

- Please remove priority claims (e.g., "this is the firs time that we...")

Response: As suggested, the priority claim was removed.

Dear Prof. Pellegata,

Thank you very much for submitting your revised manuscript. The three referees have now provided their reports (copied below). As you will see, referees #1 and #2 are overall satisfied with the revisions pending text
modifications/clarifications. However, referee #3 remains unconvinced by the data due to the unclear phospho-Tie2 staining (also mentioned by referee #1), which affects the main message of the manuscript. I have consulted with the three referees on that particular point, and we are aware of the technical limitations due to the sub-optimal efficacy of pTie2 antibody. Therefore, I would like to give you the opportunity to indicate if and how you would like to address this point.

Referee #2 mentioned:
the authors need to show clear-cut pictures of NF-PitNET tumour sections, co-localizing for P-TIE2 and markers of Non-functioning pituitary adenoma cells (SF1, alpha-GSU, either in human NF-PitNETs or in rat MENX tumors, or in both. This could be done with a good confocal microscope with an excellent objetive and zoom, or even now improved with super-resolution.

Referee #3 suggested:
Maybe the authors could use flow cytometry of freshly dispersed tumors. It is possible that the anti-pTIE2 Ab would work better by flow (after fix and perm). Flow could use antibodies specific to the cancer cells and endothelial cells to identify (and indeed better quantify) pTIE2 signal. Again, not a trivial experiment, as pTIE2 may be labile.

Again, we are aware that such experiments might be difficult, and would therefore appreciate your input before making a final decision.

With my best wishes,

Lise

Referee #1:
Comments on Novelty/Model System for Author:
Lots of work, and I think there is really an interesting finding. The quality of some experiments was insufficient and the writing is still not optimally streamlined so that clarity would be better.

Remarks for Author:
The authors have done a respectful amount of work to improve the manuscript. I am mostly happy with the revised form.

Just a few nuances below:
1. I think the authors should let an experienced native English speaking person to streamline the text. For example, if this would be my manuscript, I would change the wording of the

title and abstract as indicated below:

Angpt2/Tie2 autostimulatory loop controls pituitary tumorigenesis

Abstract
Invasive nonfunctioning (NF) pituitary neuroendocrine tumors (PitNETs) are non-resectable neoplasms associated with frequent relapses and significant comorbidities. As the current therapies of NF-PitNETs often fail, new therapeutic targets are needed. The observation that circulating angiopoietin-2 (ANGPT2) is elevated in NF-PitNET patients and correlates with tumor aggressiveness prompted us to investigate the ANGPT2/TIE2 axis in NF-PitNETs in the GH3 PitNET cell line (GH3), primary human NF-PitNET cells, mouse xenografts in zebrafish, and in MENX rats, the only autochthonous NF-PitNET model. We show that PitNET cells express a functional TIE2 receptor and secrete bioactive ANGPT2, which promotes, besides angiogenesis, tumor cell growth in an autocrine and paracrine fashion.
ANGPT2 stimulation of TIE2 in the tumor cells activates downstream cell proliferation signals, as previously demonstrated in endothelial cells (ECs). Tie2 gene deletion blunts PitNETs growth in xenograft models, and pharmacological inhibition of Angpt2/Tie2 signaling antagonizes PitNETs in cell culture and in tumor xenografts and MENX rats. Thus the ANGPT2/TIE2 axis provides an exploitable therapeutic target in NF-PitNETs, and possibly in other tumors expressing ANGPT2/TIE2. The ability of tumor cells to coopt endothelial-specific angiogenic signals expands our view on the microenvironmental cues that are essential for tumor progression.

2. p. 4, line 4 from top: One should never claim something has never been investigated! Claims like this are unprofessional.
3. When something is said to be on the membrane, the authors probably mean plasma membrane. Cells are full of membranes...
4. p. 14 "...distributed mainly around the cells..." should probably be distributed on the cell surface...
5. P. 14: "Given that rhAngpt2 is not internalized..." Please provide a reference or modify the claim.
6. I would put little weight on the result shown in SFig. 10!!! Faithful detection of Tie2-P in tumors is problematic.
7. 7. In Fig. S11 a, the upper band is marked *glycosylated Tie2, but no evidence is provided. Could the lower band be Tie1? Based on my experience, something is wrong here. Tie2 should be the upper band, which is the one that should be tyrosyl phosphorylated. Maybe the authors should try another antibody?

The authors are almost there, but I think they, too, want that everything reported stands the test of time...

Referee #2:
Comments on Novelty/Model System for Author:
My main concern in V1 was the GH3 cell line (somatotrophs instead of Non-functioning pituitary cells). But in this 2nd version they have modulated the

importance of this model and performed better research using primary cultures of the NF-PitNETs from the MENX rats.
They also have interrogated better their results, with nice findings (double IF in the tumours, growth curve in the MENX rat treated with inhibitor...)

Remarks for Author:
The v2 of Ms. EMM-2021-14364 is much improved. The data have been deeply interrogated, and many concerns have been answered.
Now, I have a list of minor issues that need to be addressed by changes in the text, for the manuscript to be clearer, less confusing and more scientifically precise language.

1) Regarding the GH3 cells, the authors had agreed that is a pituitary cell line expressing Pit1 transcription factor and "secreting" growth hormone (GH). Thus, it would be a perfect model for a functioning secretory pituitary adenoma, called Somatotropinoma or in the nomenclature of this manuscript GH-PitNET. But it is not "a good model" of Non-functioning Pituitary Adenoma, or NF-PitNET. As listed in my comments to V1, I understand why it is used as an in vitro model to obtain some molecular information. But, since there are some normal somatotrophs (GH) expressing Angpt2 in the normal pituitary, there is the possibility that GH3 is a model of normal function of the Angpt2 in the pituitary, instead of a model of the tumour expression of the Angpt/Tie-2 in the pituitary adenomas.
Following the above, some sentences must be amended (Pages are called as in the Merged PDF):

-Page 2, Abstract: <<We employed a suitable PitNET cell line (GH3), primary...>>
Change to non-qualifying: For ex: <<We employed a rat pituitary cell line, ..>>
-Page 10 bottom, Results: <<Therefore GH3 cells best recapitulate the situation seen in primary NFPitNETs, and were selected for further studies.>>
Delete this sentence. The next sentence is clear enough: GH3 cells are not NF-PitNET cells, however...
-Page 17 middle, Results: <<In conclusion, abrogation of Tie2 function in PitNET cells reduced their growth in vivo likely because it impaired their response to angiopoietins present in the TME.>>
Misleading. Change "in PitNET cells" to "in this PitNET cell line"

2) Homogeneous nomenclature: The authors have chosen the pathological nomenclature over the endocrinology nomenclature for pituitary tumours. However, non-functioning pituitary adenomas have a different cell of origin, affect different kind of patients and have different course of the disease that each of the secretory endocrine tumours. Thus, it is important to keep a homogeneous name for the whole of the pituitary adenomas (PitNET in this manuscript), and a homogeneous name for the subset of non-functioning pituitary tumours, the main topic of this manuscript. The authors mix both names in many instances, confusing the reading.
At least, the following PitNETs should be changed to NF-PitNETS:

-page 9: TIE2 positivity was also observed in cytoplasm and membrane of human PitNET cells
-page 14: As outlined above, PitNET cells express high levels of Angpt2 and

secrete it as bioactive cytokine, whereas

-page 19 (2x): The data presented here supports the existence of an autocrine/paracrine signaling involving angiopoietins (mainly Angpt2) in PitNET cells (Fig. 10). We discovered that Angpt2 is highly expressed in PitNET cells, which...
-page 20 (2x): How important an active Tie2 receptor is in PitNET progression is further.. Remarkably, PitNETs in MENX rats showed virtually no...
-page 21, Conclusions: or Tie2 inhibitors not only in PitNETs but in general in tumors found to
-Page 41: Suppl Fig 3 legend: Expression of Angpt1, Angpt2 and Tie2 in human PitNETs and control ECs.
-page 44: Suppl Fig S5: Section a) (It is missing the letter in the figure): Change in this figure: PitNETs cells for NF-PitNET cells.
-page 78: Figure 8, sections g-h-i-j: It is very confusing, not only by the lack of NF- , but also for a different name in g/I compared to h/j-
Could you write a generic Human NF-PitNET over the four sections ?
-page 80, Figure 10: Summary Abstract: Change PitNET cell to NF-PitNET cell

3) TIE2 expression in "unaffected/normal" pituitaries: While expression of ANGPT2 and ANGPT1 in normal pituitary endocrine cells (immunohistochemistry/immunofluorescence) is well described, I cannot find the same for TIE2. In page 9, second paragraph, it is described TIE2 in ECs in normal pituitary, but nothing is explained regarding the endocrine cells. Please, state that endcrine cells are negative for TIE2 if this is the case, or alternatively describe any other positive staining in addition to EC cells.

4) Page 9, third paragraph: <<In the laboratory, we work with MENX rats, the only...>>
Casual phrasing. Delete. <<MENX rat is the only spontaneous, autochthonous...

5) Page 10, middle: <<.. we setout to investigate it.>>
Verb. Set out

6) Page 14, This peculiar sentence is confusing:
<<Discrete PLA spots were counted and the results showed that 34.5% (660/1914) of tumor cells had at least one positive interaction between Tie2 and Angpt2, with the majority of the cells showing 1 interaction (Fig. 5b).>>

7) Page 18: We validated the expression of ANGPT2 and Tie2 in few primary cultures
Change few to some

Referee #3:
Remarks for Author:
I feel that the new data provided to address my comment #1 (TIE2 activation in PNET cells - in vivo models) are not compelling. In both Figures 6e and S10, pTIE2 (and ANGPT2) staining appear restricted to vascular structures, while a

low and diffuse but potentially non-specific signal for both pTIE2 and ANGPT2 antibodies is observed in the cancer cells.

I do appreciate that antibodies against pTIE2 or ANGPT2 may work suboptimally, but the data seem to show reliable staining of vascular structures (which are known to express both TIE2 and ANGPT2) but not cancer cells. Also, I note non-specific pTIE2 staining of intravascular red blood cells in Figure S10. (The white arrows do not seem to point to pTIE2+ endothelial cells; actually, they point to ANGPT2+ cells of unclear nature.)

So, unfortunately I remain unconvinced of this dataset.

The paper provides additional evidence that TIE2 is expressed in PNET cancer cells, but I wonder if the authors stand strongly by the immunofluorescence data that, in my view, would be critical to support the main message of the paper.

Dear Dr. Roth,

We are very pleased that Reviewers #1 and #2 are satisfied with the revised version of our article, and are only asking for minor text revisions. We are happy to address the remaining issues, and modify and improve the article as suggested. We are especially grateful to Reviewer #1 who was very proactive, and provided us with an improved wording of both title and Abstract.

We are also pleased that both Reviewers #1 and #2 acknowledge the “respectful amount of work” (quoting Reviewer #1) that we included in the revised version of the manuscript thanks to their specific comments, and which significantly improved it.

Coming to the issue of Tie2 activation in pituitary tumor cells, I would like to stress the point that none of the three Reviewers has ever disputed that Tie2 is phosphorylated upon Angpt2-stimulation of pituitary tumor cells as shown by western blotting in Figure 6c,d, and by immunofluorescence in Figure 6a,b. The same applies for the phosphorylation of Tie2 found in rat tumor tissues and shown in Figure 9f. Importantly, the expression of P-Tie2 in rat tumors (but not of total Tie2) was lost upon treatment with the angiopoietin-sequestering peptibody AMG386, and not upon placebo treatment (as shown in Figure 9f and S17b), attesting to the specificity of the stainings.
All these results were obtained using an antibody against P-Tie2-Y1102/1108 from Millipore, which was often cited in the literature and worked really well on **rat** cells/tissues.

Moreover, none of the Reviewers disputed that Tie2 in pituitary tumor cells plays an important role in tumorigenesis, as demonstrated by the xenograft experiments using Tie2 KO tumor cells.

In this context, I find highly relevant that Reviewer #1, who is aware that a “..f*aithful detection of Tie2-P in tumors is problematic*”, is overall very positive about our study (“..*Lots of work, and I think there is really an interesting finding*”). This implies that, in his/her opinion, the issue of the detection of P-Tie2 in tumor tissues is not absolutely crucial for the main message of the article.

Reviewer #3 raised one main criticism: he/she was not convinced about Tie2 phosphorylation in pituitary tumor cells. The original comment read: “..*There is no convincing evidence that Tie2 is phosphorylated in the cancer cells of pituitary tumors (Fig S5). The image in Fig S5a shows clear phosphorylation in vascular cells, but only mild and scarce staining in non-vascular cells. This dataset should be improved by inclusion of additional data (human and rat tumors; quantification of the data in cancer cells versus vascular cells, etc.), and the results should be presented in the main figures, being a key message of the study*…“.

To address this point, we conducted additional stainings for P-Tie2 on the rat pituitary tumor tissues. These stainings (obtained using the validated antibody from Millipore) are now illustrated in Figure 6e and Figure S10, and clearly show that in the tumor areas there is a co-localization of Angpt2 and P-Tie2.

After reading the revised version, Reviewer #3 wrote: “….*I feel that the new data provided to address my comment #1 (TIE2 activation in PNET cells - in vivo models) are not compelling. In both Figures 6e and S10, pTIE2 (and ANGPT2) staining appear restricted to vascular structures, while a low and diffuse but potentially non-specific signal for both pTIE2 and ANGPT2 antibodies is observed in the cancer cells. I do appreciate that antibodies against pTIE2 or ANGPT2 may work suboptimally, but the data seem to show reliable staining of vascular structures (which are known to express both TIE2 and ANGPT2) but not cancer cells. Also, I note non-specific pTIE2 staining of intravascular red blood cells in Figure S10. (The white arrows do not seem to point to pTIE2+ endothelial cells; actually, they point to ANGPT2+ cells of unclear nature.)*

The paper provides additional evidence that TIE2 is expressed in PNET cancer cells, but I wonder if the authors stand strongly by the immunofluorescence data that, in my view, would be critical to support the main message of the paper....”

I realized that in the legend of Fig. S10, a few words are missing by mistake, and this may have affected the interpretation of the results. I apologize for this oversight.

The figure legend should read: "…(**b**) *The white arrows in the enlarged top panel indicate Angpt2-positive cells in the adjacent non-tumor area; the asterisks in the enlarged bottom panel indicates P-Tie2-positive ECs, used as positive control*…"

The correct interpretation of the stainings shown in Figure S10 is as follows: the white arrows indicate the sparse Angpt2-positive cells that we have always observed in the normal/non-tumorous pituitary parenchyma in rats and humans (also shown in Figures 1c, 2b, S2c, S2f). The asterisks indicate tumor-associated endothelial cells, used as positive control for the P-Tie2 antibody.
To note, erythrocytes have strong autofluorescence; this does not imply that the antibody is not specific.

In both Figures 6e and S10, expression of P-Tie2 is not only shown by vessels, but also by the pituitary tumor cells. This staining is actually specific: it is present in cells in the tumor area but it is absent in cells in adjacent non-tumor areas.

Finally, the fact that P-Tie2 and Angpt2 co-localize in the tumor areas further strengthen our hypothesis of an autocrine loop involving Angpt2 and Tie2 in these cells.

The last comment of Reviewer #3 refers to the immunofluorescence data on the co-localization of Tie2 with markers of pituitary gonadotroph/tumor cells (e.g. SF-1) (Figure S4 and panel attached here below for easy reference).
We really believe that the staining of Tie2 is specific and shows high expression in the tumor cells, especially in the large tumors.

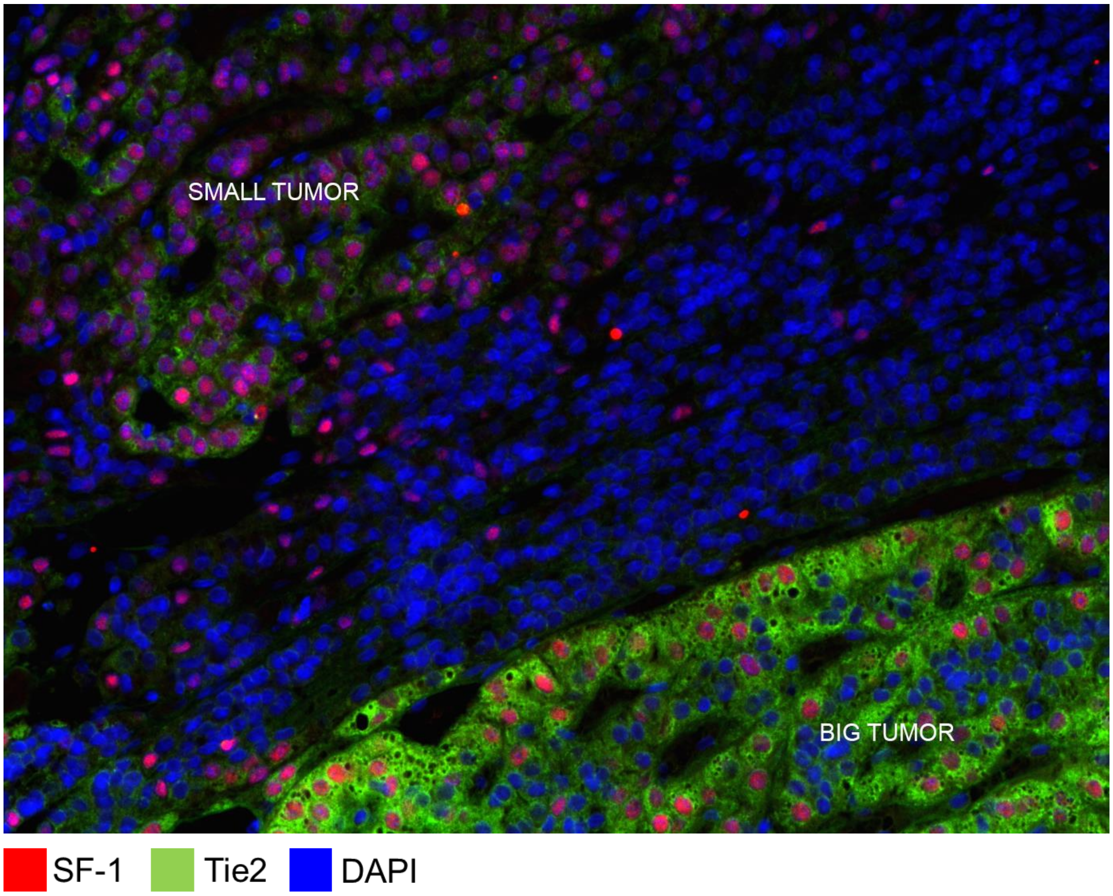

Despite the additional data provided, unfortunately, Reviewer 3 is still not fully convinced about the staining results, and suggests the following experiment:

"..*Maybe the authors could use flow cytometry of freshly dispersed tumors. It is possible that the anti-pTIE2 Ab would work better by flow (after fix and perm). Flow could use antibodies specific to the cancer cells and endothelial cells to identify (and indeed better quantify) pTIE2 signal. Again, not a trivial experiment, as pTIE2 may be labile*".

During the revision of the manuscript, we run out of the original antibody from Millipore, now discontinued. We tested several anti-P-Tie2 antibodies: SAB5403999 (Y1108 from SIGMA), AF3909 (Y1102/1100 from R&D Systems), AF2720 (Y992 from R&D Systems), ABF131 (Y992 from Merck-Sigma). These antibodies, with the exception of AF2720, gave no specific bands by western blotting of rat tumor cells or rat endothelial cells (RAOECs) used as positive control. We even used a more sensitive method (i.e. capillary electrophoresis), as well as angiopoietin-stimulated RAOECs, but we did not obtain specific bands.
With the anti-P-Tie2 AF2720, we obtained a band at the correct size in RAOECs and in primary rat tumor cells upon Angpt1/2 stimulation (now shown in Fig. S11). None of these antibodies ever worked in immunofluorescence of RAOECs control cells (here attached are exemplary negative results from currently available antibodies).
These anti-P-Tie2 antibodies were validated in human samples, and eventually in mouse samples. Rat samples are not routinely tested. This is why in the revised article we included RAOECs (in addition to HUVECs) to have a positive control for our target species.

Reviewer #3 now suggests a difficult experiment that, although theoretically interesting, he/she also says that it might not work. Even having a working antibody, the phosphorylation of the receptor would likely not be stable enough to stand the extensive processing of the tissues to obtain the cell suspension. In addition, we could not find a suitable anti-P-Tie2 antibody on the market that works reliably on rat cells. Therefore, we agree with the Reviewer him/herself that this approach will not guarantee that we gain additional information. Finally, this experiment requires the use of many 8-month-old mutant rats, which, due to the Corona-restricted breeding capacity, will take months to obtain.
The low efficacy of the current anti-P-Tie2 antibodies also impacts the staining experiments of the rat tumors suggested by Reviewer #2.

We feel that the potential results of these additional experiments will not provide new insights, but will mainly be confirmatory, given that the main message of the study (= the existence of a pathway that supports the growth of pituitary tumor cells, which can be exploited as therapeutic target) is demonstrated by data already provided:

a) pituitary tumor cells secrete a bioactive Angpt2 (Figure 4a,b);
b) silencing of Angpt2 expression reduces proliferation/viability of pituitary tumor cells in vitro, rescued by rhANGPT2 (Figure 3c-h);
c) interaction of Angpt2 and Tie2 receptor on the plasma membrane of tumor cells (Figures 5, S8);
e) stimulation of pituitary tumor cells with rhAngpt2 activates Tie2 phosphorylation and downstream signaling (Figures 6c-e, S11, S13);
f) treatment of primary pituitary tumor cells with AMG386 or Tie2-kinase inhibitor inhibits their viability (Figure 8);
g) sequestering angiopoietins with AMG386 in mouse xenografts or in MENX rats (with Tie2-expressing tumors) suppresses tumor growth and tumor cell proliferation in vivo (Figure 9).

Finally, I would like to reiterate that proof of the important role of Tie2 in pituitary tumorigenesis is provided by the xenograft experiments using Tie2 KO pituitary tumor cells (Figure 7).

As competition is high, we would thus respectfully request to omit the proposed extra experiments.

With my best regards,

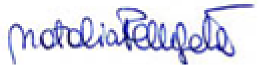

Natalia Pellegata

Figures for reviewers removed.

RAOEC anti-P-Tie2 SAB5403999

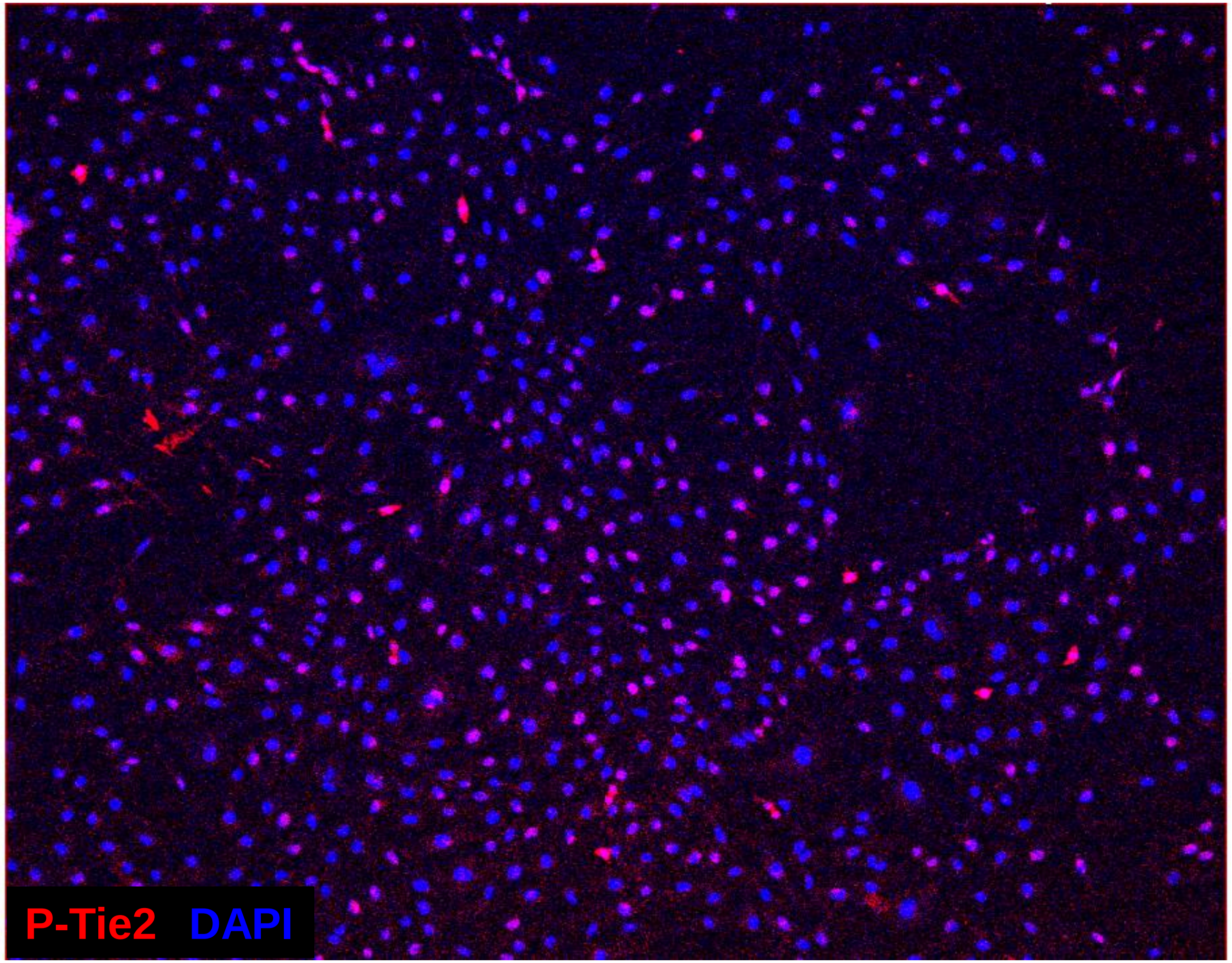

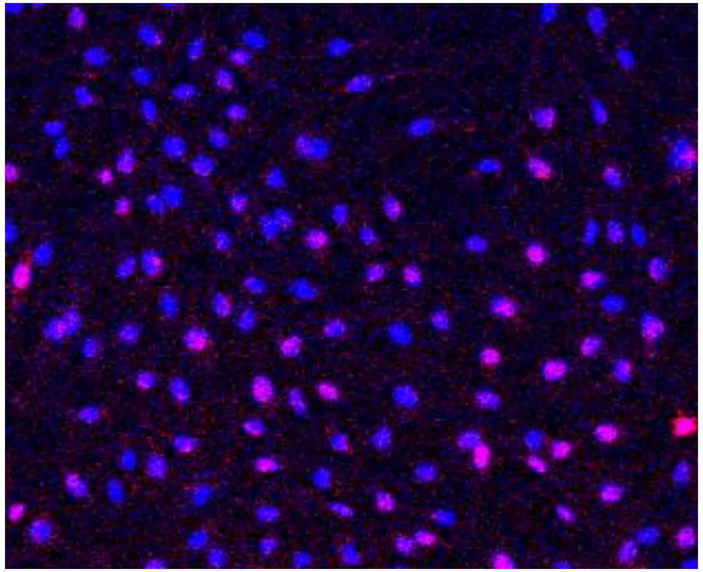

Unspecific signal in the nucleus

RAOECs anti-P-Tie2 AF3909 | RAOEC anti-P-Tie2 AF2720 | RAOEC + rhANGPT2 anti-P-Tie2 AF2720

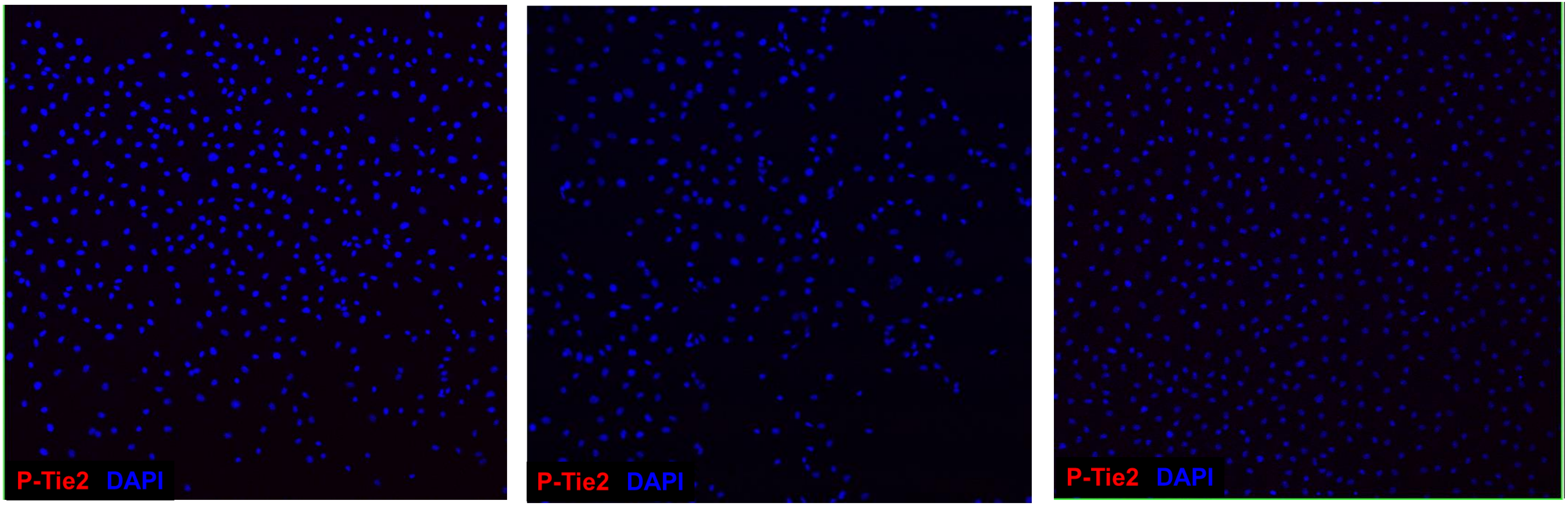

No staining

Stainings of Rat Tissues

P-Tie2 (AF2720)

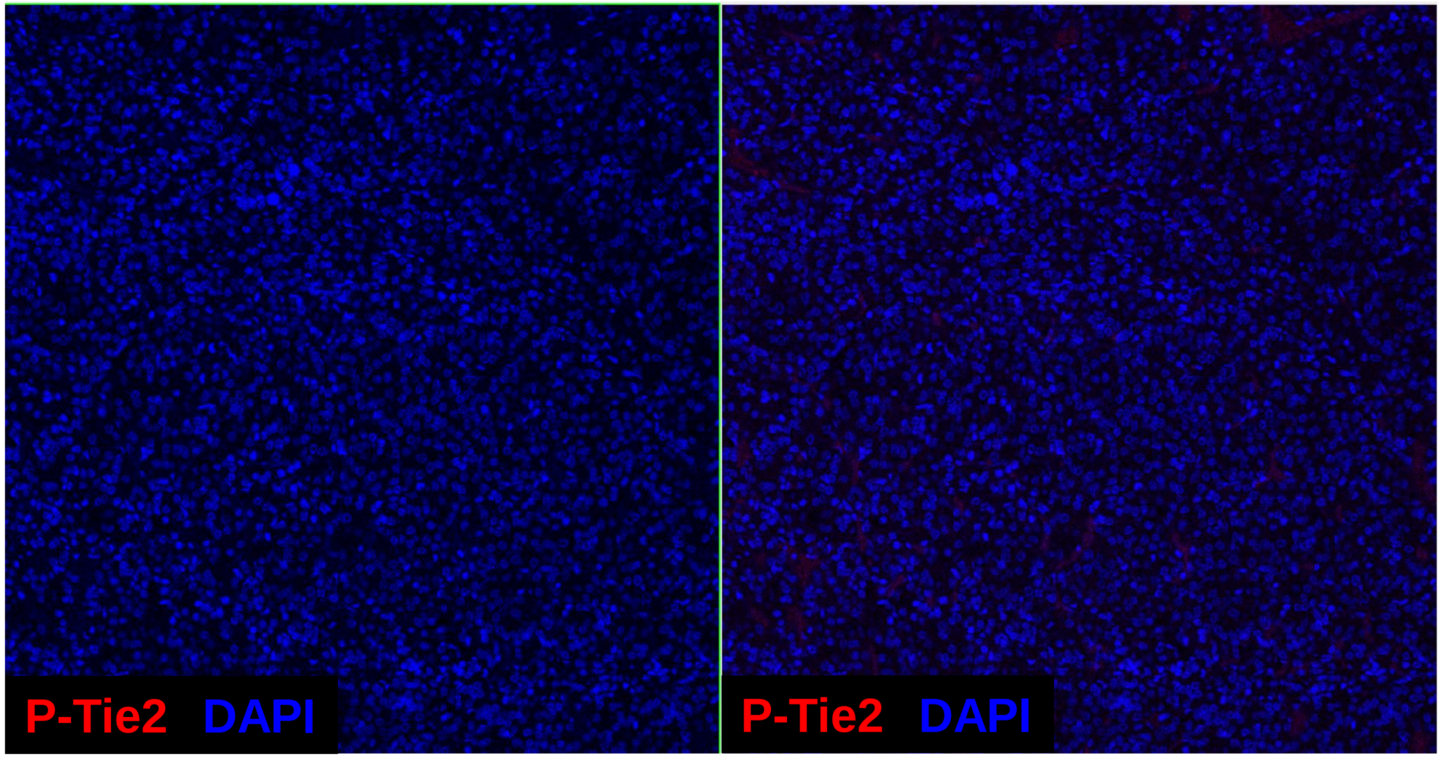

No staining

11th Jan 2022

Dear Prof. Pellegata,

Thank you for providing a provisional point-by-point letter to address the concern raised by referee #3. We have now heard back from this referee, who stated:
"I read the rebuttal letter and recommend publication of the study without the addition of flow cytometry data (I agree with the authors that the bulk of data support the conclusion that an autostimulatory ANGPT2/TIE2 loop exists in cancer cells, at least ex vivo).
However, I would encourage the authors to make appropriate text revisions that inform the reader of some of the uncertainties and technical limitations surrounding the use of the pTIE2 antibody for staining of the tumors."

Therefore, we would like you to make the text revisions suggested by this referee, and to also address the remaining minor concerns from referees #1 and #2.

Additionally, please address the following editorial points:

- Please provide a .docx formatted version of the manuscript text (including legends for main figures, EV figures and tables). Please make sure that the changes are highlighted to be clearly visible.
- Please include a .docx formatted letter INCLUDING the reviewers' reports and your detailed point-by-point responses to their comments.
- We do not have abbreviation list, please introduce the abbreviations as they appear in the text.
- The Material and Methods section should be placed after the Discussion. As we do not have size restrictions, please include the supplemental M&M in the main manuscript text. The "ethics approval" paragraph should be included in the M&M.
- Kindly remove "Conclusions" from the Discussion.
- A Data Availability section should follow the Material and Methods section. Primary datasets produced in this study need to be deposited in an appropriate public database, and the accession numbers and database listed under 'Data Availability'. Please remember to provide a reviewer password if the datasets are not yet public (see https://www.embopress.org/page/journal/17574684/authorguide#dataavailability). In case you have no data that requires deposition in a public database, please state so in this section. Note that the Data Availability Section is restricted to new primary data that are part of this study.
- The "Declarations" section should be removed. Please also delete the consent for publication.
- You currently have 10 figures, which is on the high side. Would you consider including Expanded View figures? Expanded View (EV) Figures and Tables are collapsible/expandable online. A maximum of 5 EV Figures can be typeset. EV Figures should be cited as 'Figure EV1, Figure EV2" etc... in the text and their respective legends should be included in the main text after the legends of regular figures.
- The Suppl. figures, legends and tables should be removed from the main manuscript, and compiled in a PDF labelled "Appendix". The nomenclature needs correcting: Appendix Figure S1, Appendix Table S1 etc. The appendix file needs a table of content. The reagent table could be included in the Appendix file.
- Please carefully check that all figures and figure panels are referenced in the main text. A callout is currently missing for figure 5D.
- Please provide a "Paper Explained" section: EMBO Molecular Medicine articles are accompanied by a summary of the articles to emphasize the major findings in the paper and their medical implications for the non-specialist reader. Please provide a draft summary of your article highlighting
- the medical issue you are addressing,
- the results obtained and
- their clinical impact.
This may be edited to ensure that readers understand the significance and context of the research. Please refer to any of our published articles for an example.
- Please rename the "Conflict of Interest" section.
- Every published paper now includes a 'Synopsis' to further enhance discoverability. Synopses include a short stand first (maximum of 300 characters, including space) as well as 2-5 one-sentences bullet points that summarize the paper. They should be designed to be complementary to the abstract - i.e. not repeat the same text. We encourage inclusion of key acronyms and quantitative information (maximum of 30 words / bullet point). Please use the passive voice.
Kindly also suggest a striking image or visual abstract to illustrate your article as a PNG file 550 px wide x 300-600 px high.
- Please format the references to have them in alphabetical order, with 10 authors listed before "et al".
- Please merge the Funding with Acknowledgements.
- As part of the EMBO Publications transparent editorial process initiative (see our Editorial at http://embomolmed.embopress.org/content/2/9/329), EMBO Molecular Medicine will publish online a Review Process File (RPF) to accompany accepted manuscripts.
This file will be published in conjunction with your paper and will include the anonymous referee reports, your point-by-point

response and all pertinent correspondence relating to the manuscript. Let us know whether you agree with the publication of the RPF and as here, if you want to remove or not any figures from it prior to publication.
Please note that the Authors checklist will be published at the end of the RPF.

I look forward to receiving your revised manuscript.

With my best wishes,

Lise

Lise Roth, PhD
Editor
EMBO Molecular Medicine

To submit your manuscript, please follow this link:

Link Not Available

*Additional important information regarding Figures
Each figure should be given in a separate file and should have the following resolution:
Graphs 800-1,200 DPI
Photos 400-800 DPI
Colour (only CMYK) 300-400 DPI"

Figures are not edited by the production team. All lettering should be the same size and style; figure panels should be indicated by capital letters (A, B, C etc). Gridlines are not allowed except for log plots. Figures should be numbered in the order of their appearance in the text with Arabic numerals. Each Figure must have a separate legend and a caption is needed for each panel.

*Additional important information regarding figures and illustrations can be found at https://bit.ly/EMBOPressFigurePreparationGuideline. See also figure legend preparation guidelines: https://www.embopress.org/page/journal/17574684/authorguide#figureformat

The system will prompt you to fill in your funding and payment information. This will allow Wiley to send you a quote for the article processing charge (APC) in case of acceptance. This quote takes into account any reduction or fee waivers that you may be eligible for. Authors do not need to pay any fees before their manuscript is accepted and transferred to our publisher.

***** Reviewer's comments *****

Referee #1 (Comments on Novelty/Model System for Author):

Lots of work, and I think there is really an interesting finding. The quality of some experiments was insufficient and the writing is still not optimally streamlined so that clarity would be better.

Referee #1 (Remarks for Author):

The authors have done a respectful amount of work to improve the manuscript. I am mostly happy with the revised form.

Just a few nuances below:

1. I think the authors should let an experienced native English speaking person to streamline the text.

For example, if this would be my manuscript, I would change the wording of the title and abstract as indicated below:

Angpt2/Tie2 autostimulatory loop controls pituitary tumorigenesis

Abstract

Invasive nonfunctioning (NF) pituitary neuroendocrine tumors (PitNETs) are non-resectable neoplasms associated with frequent relapses and significant comorbidities. As the current therapies of NF-PitNETs often fail, new therapeutic targets are needed. The observation that circulating angiopoietin-2 (ANGPT2) is elevated in NF-PitNET patients and correlates with tumor aggressiveness prompted us to investigate the ANGPT2/TIE2 axis in NF-PitNETs in the GH3 PitNET cell line (GH3), primary human NF-PitNET cells, mouse xenografts in zebrafish, and in MENX rats, the only autochthonous NF-PitNET model. We show that PitNET cells express a functional TIE2 receptor and secrete bioactive ANGPT2, which promotes, besides angiogenesis, tumor cell growth in an autocrine and paracrine fashion. ANGPT2 stimulation of TIE2 in the tumor cells activates downstream cell proliferation signals, as previously demonstrated in endothelial cells (ECs). Tie2 gene deletion blunts PitNETs growth in xenograft models, and pharmacological inhibition of Angpt2/Tie2 signaling antagonizes PitNETs in cell culture and in tumor xenografts and MENX rats. Thus the ANGPT2/TIE2 axis provides an exploitable therapeutic target in NF-PitNETs, and possibly in other tumors expressing ANGPT2/TIE2. The ability of tumor cells to coopt endothelial-specific angiogenic signals expands our view on the microenvironmental cues that are essential for tumor progression.

2. p. 4, line 4 from top: One should never claim something has never been investigated! Claims like this are unprofessional.
3. When something is said to be on the membrane, the authors probably mean plasma membrane. Cells are full of membranes...
4. p. 14 "...distributed mainly around the cells..." should probably be distributed on the cell surface...
5. P. 14: "Given that rhAngpt2 is not internalized..." Please provide a reference or modify the claim.
6. I would put little weight on the result shown in SFig. 10!!! Faithful detection of Tie2-P in tumors is problematic.
7. 7. In Fig. S11 a, the upper band is marked *glycosylated Tie2, but no evidence is provided. Could the lower band be Tie1? Based on my experience, something is wrong here. Tie2 should be the upper band, which is the one that should be tyrosyl phosphorylated. Maybe the authors should try another antibody?

The authors are almost there, but I think they, too, want that everything reported stands the test of time...

Referee #2 (Comments on Novelty/Model System for Author):

My main concern in V1 was the GH3 cell line (somatotrophs instead of Non-functioning pituitary cells). But in this 2nd version they have modulated the importance of this model and performed better research using primary cultures of the NF-PitNETs from the MENX rats.
They also have interrogated better their results, with nice findings (double IF in the tumours, growth curve in the MENX rat treated with inhibitor...)

Referee #2 (Remarks for Author):

The v2 of Ms. EMM-2021-14364 is much improved. The data have been deeply interrogated, and many concerns have been answered.
Now, I have a list of minor issues that need to be addressed by changes in the text, for the manuscript to be clearer, less confusing and more scientifically precise language.

1) Regarding the GH3 cells, the authors had agreed that is a pituitary cell line expressing Pit1 transcription factor and "secreting" growth hormone (GH). Thus, it would be a perfect model for a functioning secretory pituitary adenoma, called Somatotropinoma or in the nomenclature of this manuscript GH-PitNET. But it is not "a good model" of Non-functioning Pituitary Adenoma, or NF-PitNET.
As listed in my comments to V1, I understand why it is used as an in vitro model to obtain some molecular information. But, since there are some normal somatotrophs (GH) expressing Angpt2 in the normal pituitary, there is the possibility that GH3 is a model of normal function of the Angpt2 in the pituitary, instead of a model of the tumour expression of the Angpt/Tie-2 in the pituitary adenomas.
Following the above, some sentences must be amended (Pages are called as in the Merged PDF):
-Page 2, Abstract: <>
Change to non-qualifying: For ex: <>

-Page 10 bottom, Results: <>

Delete this sentence. The next sentence is clear enough: GH3 cells are not NF-PitNET cells, however...

-Page 17 middle, Results: <>
Misleading. Change "in PitNET cells" to "in this PitNET cell line"

2) Homogeneous nomenclature: The authors have chosen the pathological nomenclature over the endocrinology nomenclature for pituitary tumours. However, non-functioning pituitary adenomas have a different cell of origin, affect different kind of patients and have different course of the disease that each of the secretory endocrine tumours. Thus, it is important to keep a homogeneous name for the whole of the pituitary adenomas (PitNET in this manuscript), and a homogeneous name for the subset of non-functioning pituitary tumours, the main topic of this manuscript. The authors mix both names in many instances, confusing the reading.
At least, the following PitNETs should be changed to NF-PitNETS:
-page 9: TIE2 positivity was also observed in cytoplasm and membrane of human PitNET cells
-page 14: As outlined above, PitNET cells express high levels of Angpt2 and secrete it as bioactive cytokine, whereas
-page 19 (2x): The data presented here supports the existence of an autocrine/paracrine signaling involving angiopoietins (mainly Angpt2) in PitNET cells (Fig. 10).
We discovered that Angpt2 is highly expressed in PitNET cells, which...
-page 20 (2x): How important an active Tie2 receptor is in PitNET progression is further..
Remarkably, PitNETs in MENX rats showed virtually no...
-page 21, Conclusions: or Tie2 inhibitors not only in PitNETs but in general in tumors found to
-Page 41: Suppl Fig 3 legend: Expression of Angpt1, Angpt2 and Tie2 in human PitNETs and control ECs.
-page 44: Suppl Fig S5: Section a) (It is missing the letter in the figure): Change in this figure: PitNETs cells for NF-PitNET cells.
-page 78: Figure 8, sections g-h-i-j: It is very confusing, not only by the lack of NF- , but also for a different name in g/I compared to h/j-
Could you write a generic Human NF-PitNET over the four sections ?
-page 80, Figure 10: Summary Abstract: Change PitNET cell to NF-PitNET cell

3) TIE2 expression in "unaffected/normal" pituitaries: While expression of ANGPT2 and ANGPT1 in normal pituitary endocrine cells (immunohistochemistry/immunofluorescence) is well described, I cannot find the same for TIE2. In page 9, second paragraph, it is described TIE2 in ECs in normal pituitary, but nothing is explained regarding the endocrine cells. Please, state that endcrine cells are negative for TIE2 if this is the case, or alternatively describe any other positive staining in addition to EC cells.

4) Page 9, third paragraph: <>
Casual phrasing. Delete. <

5) Page 10, middle: <<.. we setout to investigate it.>>
Verb. Set out

6) Page 14, This peculiar sentence is confusing:
<>

7) Page 18: We validated the expression of ANGPT2 and Tie2 in few primary cultures
Change few to some

Referee #3 (Remarks for Author):

I feel that the new data provided to address my comment #1 (TIE2 activation in PNET cells - in vivo models) are not compelling. In both Figures 6e and S10, pTIE2 (and ANGPT2) staining appear restricted to vascular structures, while a low and diffuse but potentially non-specific signal for both pTIE2 and ANGPT2 antibodies is observed in the cancer cells.

I do appreciate that antibodies against pTIE2 or ANGPT2 may work suboptimally, but the data seem to show reliable staining of vascular structures (which are known to express both TIE2 and ANGPT2) but not cancer cells. Also, I note non-specific pTIE2 staining of intravascular red blood cells in Figure S10. (The white arrows do not seem to point to pTIE2+ endothelial cells;

actually, they point to ANGPT2+ cells of unclear nature.)

So, unfortunately I remain unconvinced of this dataset.

The paper provides additional evidence that TIE2 is expressed in PNET cancer cells, but I wonder if the authors stand strongly by the immunofluorescence data that, in my view, would be critical to support the main message of the paper.

Manuscript #: EMM_2021_14364_V2

Point-to-point rebuttal to the reviewers' comments.

Referee #1:

1. Reviewer: I think the authors should let an experienced native English speaking person to streamline the text.

Answer: *According to the Reviewer's comment, a native speaker co-author (GE) has corrected the English.*

2. Reviewer: For example, if this would be my manuscript, I would change the wording of the title and abstract as indicated below:

- Angpt2/Tie2 autostimulatory loop controls pituitary tumorigenesis

- Abstract

  Invasive nonfunctioning (NF) pituitary neuroendocrine tumors (PitNETs) are non-resectable
  neoplasms associated with frequent relapses and significant comorbidities. As the current therapies of NF-PitNETs….

Answer: *We have now changed both title and Abstract accordingly.*

3. Reviewer: p. 4, line 4 from top: One should never claim something has never been investigated! Claims like this are unprofessional.

Answer: *The sentence has been re-written.*

4. Reviewer: When something is said to be on the membrane, the authors probably mean plasma membrane. Cells are full of membranes...

Answer: *We agree with the Reviewer. We have now added "plasma" or "cell" next to the word "membrane" to specify which membrane we refer to.*

5. Reviewer: p. 14 "...distributed mainly around the cells..." should probably be distributed on the cell surface...

Answer: *The text has been amended.*

6. Reviewer: P. 14: "Given that rhAngpt2 is not internalized..." Please provide a reference or modify the claim.

Answer: *We have modified the sentence and added the appropriate reference.*

7. Reviewer: I would put little weight on the result shown in SFig. 10!!! Faithful detection of Tie2-P in tumors is problematic.

Answer: *As also suggested by Reviewer #3, we have now added a comment concerning the staining of tumor tissues using the anti-P-Tie2 antibody on page 12, which reads as follows:*

*..”*Albeit staining tumor tissues with anti-P-Tie2 antibodies has technical limitations, we found that P-Tie2 and Angpt2 co-localize in the tumors,…”

We also added a comment on page 15:

..” While acknowledging possible technical limitations, these staining results make P-Tie2 a useful readout of drug response.”

8. Reviewer: In Fig. S11 a, the upper band is marked *glycosylated Tie2, but no evidence is provided. Could the lower band be Tie1? Based on my experience, something is wrong here. Tie2 should be the upper band, which is the one that should be tyrosyl phosphorylated. Maybe the authors should try another antibody?

Answer*: The Reviewer is correct: although Tie2 has been reported to be glycosylated in endothelial cells (reviewed in Saharinen P et al. 2015 The TIE Receptor Family. In: Wheeler D., Yarden Y. (eds) Receptor Tyrosine Kinases: Family and Subfamilies. Springer, Cham), we did not offer experimental proof that it occurs in our rat aortic endothelial cells. Therefore, we deleted this mark.*
We have used two different anti-Tie2 antibodies for western blotting (see figure below). The one from Millipore was used for Fig. S11. We obtained two main bands using both antibodies (see figure below). Two bands were also seen by capillary electrophoresis (not shown). The lower Tie2 band corresponds to the band that is detected by the anti-P-Tie2 antibody (figure below, right panel). In the literature, the Tie2 receptor has an apparent molecular mass that goes from ca. 120 kDa to 160 kDa depending on the samples/experimental conditions/glycosylation. So, the lower band is compatible with the native Tie2. To avoid confusion, we revised Figure S11 to highlight the relevant band.

Figure for referees removed.

Referee #2:

1. Reviewer: Regarding the GH3 cells, the authors had agreed that is a pituitary cell line expressing Pit1 transcription factor and "secreting" growth hormone (GH). Thus, it would be a perfect model for a functioning secretory pituitary adenoma, called Somatotropinoma or in the nomenclature of this manuscript GH-PitNET. But it is not "a good model" of Non-functioning Pituitary Adenoma, or NF-PitNET. As listed in my comments to V1, I understand why it is used as an in vitro model to obtain some molecular information. But, since there are some normal somatotrophs (GH) expressing Angpt2 in the normal pituitary, there is the possibility that GH3 is a model of normal function of the Angpt2 in the pituitary, instead of a model of the tumour expression of the Angpt/Tie-2 in the pituitary adenomas. Following the above, some sentences must be amended (Pages are called as in the Merged PDF):

1a: Page 2, Abstract: <<We employed a suitable PitNET cell line (GH3), primary...>>
Change to non-qualifying: For ex: <<We employed a rat pituitary cell line, ..>>

Answer: *The text has been amended according to the Reviewer’s suggestion.*

1b: Page 10 bottom, Results: <>

Delete this sentence. The next sentence is clear enough: GH3 cells are not NF-PitNET cells, however...

Answer: *The sentence has been deleted.*

1c: Page 17 middle, Results: <>

Misleading. Change "in PitNET cells" to "in this PitNET cell line"

Answer: *The text has been amended.*

2. Reviewer: Homogeneous nomenclature: The authors have chosen the pathological nomenclature over the endocrinology nomenclature for pituitary tumours. However, non-functioning pituitary adenomas have a different cell of origin, affect different kind of patients and have different course of the disease that each of the secretory endocrine tumours. Thus, it is important to keep a homogeneous name for the whole of the pituitary adenomas (PitNET in this manuscript), and a homogeneous name for the subset of non-functioning pituitary tumours, the main topic of this manuscript. The authors mix both names in many instances, confusing the reading.

At least, the following PitNETs should be changed to NF-PitNETS:
-page 9: TIE2 positivity was also observed in cytoplasm and membrane of human PitNET cells
-page 14: As outlined above, PitNET cells express high levels of Angpt2 and secrete it as bioactive cytokine, whereas
-page 19 (2x): The data presented here supports the existence of an autocrine/paracrine signaling involving angiopoietins (mainly Angpt2) in PitNET cells (Fig. 10).
We discovered that Angpt2 is highly expressed in PitNET cells, which...

-page 20 (2x): How important an active Tie2 receptor is in PitNET progression is further.. Remarkably, PitNETs in MENX rats showed virtually no...
-page 21, Conclusions: or Tie2 inhibitors not only in PitNETs but in general in tumors found to
-Page 41: Suppl Fig 3 legend: Expression of Angpt1, Angpt2 and Tie2 in human PitNETs and control ECs.
-page 44: Suppl Fig S5: Section a) (It is missing the letter in the figure): Change in this figure: PitNETs cells for NF-PitNET cells.
-page 78: Figure 8, sections g-h-i-j: It is very confusing, not only by the lack of NF- , but also for a different name in g/I compared to h/j-
Could you write a generic Human NF-PitNET over the four sections ?
-page 80, Figure 10: Summary Abstract: Change PitNET cell to NF-PitNET cell

Answer: *We thank the Reviewer for pointing out these inconsistences. We modified the text according to these suggestions. We have also modified Figure 8 (now Figure EV4) as suggested to improve clearity.*

3. Reviewer: TIE2 expression in "unaffected/normal" pituitaries: While expression of ANGPT2 and ANGPT1 in normal pituitary endocrine cells (immunohistochemistry/immunofluorescence) is well described, I cannot find the same for TIE2. In page 9, second paragraph, it is described TIE2 in ECs in normal pituitary, but nothing is explained regarding the endocrine cells. Please, state that endcrine cells are negative for TIE2 if this is the case, or alternatively describe any other positive staining in addition to EC cells.

Answer: *Following the Reviewer's comment, we have included a sentence about the expression of TIE2 on page 6, which reads as follows:*

..” Pituitary cells in the control tissues showed no Tie2 staining.”

4. Reviewer: Page 9, third paragraph: <>
Casual phrasing. Delete. <<MENX rat is the only spontaneous, autochthonous...

Answer: *The sentence has been rephrased.*

5. Reviewer: Page 10, middle: <<.. we setout to investigate it.>>
Verb. Set out

Answer: *We corrected the spelling of this verb.*

6. Reviewer: Page 14, This peculiar sentence is confusing:
<<Discrete PLA spots were counted and the results showed that 34.5% (660/1914) of tumor cells had at least one positive interaction between Tie2 and Angpt2, with the majority of the cells showing 1 interaction (Fig. 5b).>>

Answer: *The sentence has been rephrased.*

7. Reviewer: Page 18: We validated the expression of ANGPT2 and Tie2 in few primary

cultures
Change few to some

Answer: *The word has been changed.*

Referee #3:

1. **Reviewer:**…I would encourage the authors to make appropriate text revisions that inform the reader of some of the uncertainties and technical limitations surrounding the use of the pTIE2 antibody for staining of the tumors.

Answer: *To address the Reviewer's point, we have now added a comment concerning the staining of tumor tissues using the anti-P-Tie2 antibody on page 12, which reads as follows:*

*..”*Albeit staining tumor tissues with anti-P-Tie2 antibodies has technical limitations, we found that P-Tie2 and Angpt2 co-localize in the tumors,…”

We also added a comment on page 15:

..” While acknowledging possible technical limitations, these staining results make P-Tie2 a useful readout of drug response.”

26th Jan 2022

Dear Prof. Pellegata,

Thank you for providing your revised files. I am pleased to inform you that we will be able to accept your manuscript once the following minor editorial concerns will be addressed:

1/ Main manuscript text
- Please remove the red text.
- Material and methods:
o Human samples: please include the full sentence that that the experiments conformed to the principles set out in the WMA Declaration of Helsinki and the Department of Health and Human Services Belmont Report.
o Please indicate the gender and origin of the rats used in the study.
- Please add a "conflict of interest" section. We updated our journal's competing interests policy in January 2022 and request authors to consider both actual and perceived competing interests (https://www.embopress.org/competing-interests).
- Please remove "Data not shown" (p. 13). As per our guidelines, all data referred to in the paper should be displayed in the main or Expanded View figures.

2/ Checklist:
Section F18: please indicate "This study includes no data deposited in external repositories."

3/ We would also encourage you to include the source data for figure panels that show essential data. Numerical data should be provided as individual .xls or .csv files (including a tab describing the data). For blots or microscopy, uncropped images should be submitted (using a zip archive if multiple images need to be supplied for one panel). Additional information on source data and instruction on how to label the files are available at
.

4/ Thank you for providing "The paper explained". I added minor modifications, please let me know if you agree with the following or amend as you see fit:

Problem:
Non-functioning pituitary neuroendocrine tumors (NF-PitNETs) are the second most common type of PitNETs and affect patients' functional status and mortality. Given the lack of symptoms linked to hormone hypersecretion, NF-PitNETs are often diagnosed upon tumor mass detection. At this stage, 50% of the tumors have invaded surrounding structures and cannot be completely removed by surgery. Relapse is thus frequent, causing significant comorbidities. These tumors do not respond to standard-of-care treatments, which makes the identification of novel therapeutic targets mandatory to improve patients' management.
Results:
Circulating levels of the angiopoietin 2 (ANGPT2) cytokine are elevated in NF-PitNET patients, and correlate with tumor aggressiveness. NF-PitNET cells express and secrete ANGPT2, which stimulates the proliferation/survival of tumor cells in vitro, and angiogenesis in PiNET cell xenografts in zebrafish embryos in vivo. Noteworthy, NF-PitNET cells possess a functional TIE2 receptor, which is activated by ANGPT2 and further stimulates downstream mitogenic signals. This establishes an autocrine/paracrine stimulatory loop in NF-PitNET cells, as demonstrated in ECs. Deletion of TIE2 in PitNET cells suppresses their growth in mouse xenografts in vivo. Proof-of-principle pharmacological inhibition of ANGPT2/TIE2 signaling antagonizes NF-PitNETs in primary tumor cultures, as well as in mouse xenografts and in MENX rats, the only model of spontaneous NF-PitNETs.
Impact:
Our study identifies an active ANGPT2/TIE2 signaling cascade in NF-PitNET cells. The role of this axis in sustaining tumor cell growth and mediating a cross-talk between tumor and endothelial cells in the tumor microenvironment makes it an attractive therapeutic target for the treatment of NF-PitNETs.

5/ Thank you for providing a synopsis text. I added minor modifications to fit our style and format, please amend as you see fit:
"There is currently no treatment for non-functioning pituitary neuroendocrine tumors (NF-PitNETs), which are intracranial tumors associated with frequent relapse and severe comorbidities. This study establishes the ANGPT2/Tie2 signaling axis as a novel therapeutic target for NF-PitNETs.
- Circulating ANGPT2 levels were elevated in NF-PitNET patients and correlated with tumor proliferation rate, an indicator of tumor aggressiveness
- Bioactive ANGPT2 was expressed and secreted by NF-PitNET cells, and promoted angiogenesis and tumor cell growth in an autocrine and paracrine fashion
- Functional TIE2 receptor was expressed by NF-PitNET cells, and was directly activated by angiopoietins
- PitNET cell growth was suppressed upon TIE2 knockout in mouse xenografts in vivo

- ANGPT2/TIE2 pharmacological inhibition reduced the growth of NF-PitNET primary tumor cells in vitro, as well as tumor xenografts and autochthonous NF-PitNETs in MENX rats."

Please also suggest a striking image or visual abstract to illustrate your article as a PNG file 550 px wide x 300-600 px high.

6/ As part of the EMBO Publications transparent editorial process initiative (see our Editorial at http://embomolmed.embopress.org/content/2/9/329), EMBO Molecular Medicine will publish online a Review Process File (RPF) to accompany accepted manuscripts.
This file will be published in conjunction with your paper and will include the anonymous referee reports, your point-by-point response and all pertinent correspondence relating to the manuscript. Let us know whether you agree with the publication of the RPF or if you want to remove any figures from it prior to publication.
Please note that the Authors checklist will be published at the end of the RPF.

I look forward to receiving your revised manuscript.

With kind regards,

Lise Roth

Lise Roth, PhD
Editor
EMBO Molecular Medicine

To submit your manuscript, please follow this link:

Link Not Available

The authors performed the requested editorial changes.

8th Feb 2022

Dear Prof. Pellegata,

Thank you for sending your revised manuscript files. I am pleased to inform you that your manuscript is accepted for publication and is now being sent to our publisher to be included in the next available issue of EMBO Molecular Medicine.

While checking your Source Data, I noticed that two slides were labeled Figure 3A, left panel (slides 1 and 3). Please correct if needed (right panel on slide 3?) and send us the corrected file via email.

Please read below for additional IMPORTANT information regarding your article, its publication and the production process.

Congratulations on your interesting work!

With kind regards,

Lise Roth

Lise Roth, Ph.D
Scientific Editor
EMBO Molecular Medicine

Follow us on Twitter @EmboMolMed

*** *** *** IMPORTANT INFORMATION *** *** ***

SPEED OF PUBLICATION
The journal aims for rapid publication of papers, using using the advance online publication "Early View" to expedite the process: A properly copy-edited and formatted version will be published as "Early View" after the proofs have been corrected. Please help the Editors and publisher avoid delays by providing e-mail address(es), telephone and fax numbers at which author(s) can be contacted.

Should you be planning a Press Release on your article, please get in contact with embomolmed@wiley.com as early as possible, in order to coordinate publication and release dates.

LICENSE AND PAYMENT:

All articles published in EMBO Molecular Medicine are fully open access: immediately and freely available to read, download and share.

EMBO Molecular Medicine charges an article processing charge (APC) to cover the publication costs. You, as the corresponding author for this manuscript, should have already received a quote with the article processing fee separately. Please let us know in case this quote has not been received.

Once your article is at Wiley for editorial production you will receive an email from Wiley's Author Services system, which will ask you to log in and will present you with the publication license form for completion. Within the same system the publication fee can be paid by credit card, an invoice, pro forma invoice or purchase order can be requested.

Payment of the publication charge and the signed Open Access Agreement form must be received before the article can be published online.

PROOFS

You will receive the proofs by e-mail approximately 2 weeks after all relevant files have been sent o our Production Office. Please return them within 48 hours and if there should be any problems, please contact the production office at

embopressproduction@wiley.com.

Please inform us if there is likely to be any difficulty in reaching you at the above address at that time. Failure to meet our deadlines may result in a delay of publication.

All further communications concerning your paper proofs should quote reference number EMM-2021-14364-V4 and be directed to the production office at embopressproduction@wiley.com.

EMBO PRESS

YOU MUST COMPLETE ALL CELLS WITH A PINK BACKGROUND ↓

PLEASE NOTE THAT THIS CHECKLIST WILL BE PUBLISHED ALONGSIDE YOUR PAPER

Corresponding Author Name: Natalia S. Pellegata
Journal Submitted to: EMBO MM
Manuscript Number: EMM-2021-14364-V2

Reporting Checklist For Life Sciences Articles (Rev. June 2017)

This checklist is used to ensure good reporting standards and to improve the reproducibility of published results. These guidelines are consistent with the Principles and Guidelines for Reporting Preclinical Research issued by the NIH in 2014. Please follow the journal's authorship guidelines in preparing your manuscript.

A- Figures
1. Data
The data shown in figures should satisfy the following conditions:

- the data were obtained and processed according to the field's best practice and are presented to reflect the results of the experiments in an accurate and unbiased manner.
- figure panels include only data points, measurements or observations that can be compared to each other in a scientifically meaningful way.
- graphs include clearly labeled error bars for independent experiments and sample sizes. Unless justified, error bars should not be shown for technical replicates.
- if n< 5, the individual data points from each experiment should be plotted and any statistical test employed should be justified
- Source Data should be included to report the data underlying graphs. Please follow the guidelines set out in the author ship guidelines on Data Presentation.

2. Captions
Each figure caption should contain the following information, for each panel where they are relevant:

- a specification of the experimental system investigated (eg cell line, species name).
- the assay(s) and method(s) used to carry out the reported observations and measurements
- an explicit mention of the biological and chemical entity(ies) that are being measured.
- an explicit mention of the biological and chemical entity(ies) that are altered/varied/perturbed in a controlled manner.

- the exact sample size (n) for each experimental group/condition, given as a number, not a range;
- a description of the sample collection allowing the reader to understand whether the samples represent technical or biological replicates (including how many animals, litters, cultures, etc.).
- a statement of how many times the experiment shown was independently replicated in the laboratory.
- definitions of statistical methods and measures:
  - common tests, such as t-test (please specify whether paired vs. unpaired), simple χ2 tests, Wilcoxon and Mann-Whitney tests, can be unambiguously identified by name only, but more complex techniques should be described in the methods section;
  - are tests one-sided or two-sided?
  - are there adjustments for multiple comparisons?
  - exact statistical test results, e.g., P values = x but not P values < x;
  - definition of 'center values' as median or average;
  - definition of error bars as s.d. or s.e.m.

Any descriptions too long for the figure legend should be included in the methods section and/or with the source data.

In the pink boxes below, please ensure that the answers to the following questions are reported in the manuscript itself. Every question should be answered. If the question is not relevant to your research, please write NA (non applicable). We encourage you to include a specific subsection in the methods section for statistics, reagents, animal models and human subjects.

USEFUL LINKS FOR COMPLETING THIS FORM

http://www.antibodypedia.com
http://1degreebio.org
http://www.equator-network.org/reporting-guidelines/improving-bioscience-research-repor
http://grants.nih.gov/grants/olaw/olaw.htm
http://www.mrc.ac.uk/Ourresearch/Ethicsresearchguidance/Useofanimals/index.htm
http://ClinicalTrials.gov
http://www.consort-statement.org
http://www.consort-statement.org/checklists/view/32-consort/66-title
http://www.equator-network.org/reporting-guidelines/reporting-recommendations-for-tumo
http://datadryad.org
http://figshare.com
http://www.ncbi.nlm.nih.gov/gap
http://www.ebi.ac.uk/ega
http://biomodels.net/
http://biomodels.net/miriam/
http://jjj.biochem.sun.ac.za
https://osp.od.nih.gov/biosafety-biosecurity-and-emerging-biotechnology/
http://www.selectagents.gov/

B- Statistics and general methods

Please fill out these boxes ↓ (Do not worry if you cannot see all your text once you press return)

1.a. How was the sample size chosen to ensure adequate power to detect a pre-specified effect size?	The sample size was determined at the time of ethics board application, specifically to obtain power greater than or equal to 80% to detect effect sizes deemed clinically relevant and observable based on prior published studies from this group and others. Sample sizes were specifically calculated for two-sample t-tests of differences between groups at the 0.05 Type I error rate using published formulas that are also available via online calculators as well as in the R statistical package.
1.b. For animal studies, include a statement about sample size estimate even if no statistical methods were used.	We have included a statement in the Statistical Results section on pg. 7, describing the power calculations as stated in the answer to the previous question.
2. Describe inclusion/exclusion criteria if samples or animals were excluded from the analysis. Were the criteria pre-established?	We did not exclude animals from our calculations once they entered the study. Only animals in good health conditions entered the studies.
3. Were any steps taken to minimize the effects of subjective bias when allocating animals/samples to treatment (e.g. randomization procedure)? If yes, please describe.	The mouse xenografts and MENX rats treated with AMG386 were randomized before being treated.
For animal studies, include a statement about randomization even if no randomization was used.	The mouse xenografts and MENX rats treated with AMG386 were randomized before being treated.
4.a. Were any steps taken to minimize the effects of subjective bias during group allocation or/and when assessing results (e.g. blinding of the investigator)? If yes please describe.	The results of the imaging were analyzed by scientists blind to the different animal groups.
4.b. For animal studies, include a statement about blinding even if no blinding was done	The results of the imaging were analyzed by scientists blind to the different animal groups.
5. For every figure, are statistical tests justified as appropriate?	For each Figure panel the statistical tests used are indicated, and were considered appropriate by a statistician, co -author of the article (Prof. D. Ankerst). Sample sizes were based on assumption of the tests performed, hence are adequate for use of the tests.
Do the data meet the assumptions of the tests (e.g., normal distribution)? Describe any methods used to assess it.	Yes, sample sizes were large enough (> 25) to meet the assumptions of the test based on the Central Limit Theorem, hence Normal probability plots were not necessary.
Is there an estimate of variation within each group of data?	Yes, all boxplots in the figures are accompanied with bars out to one standard error of the mean (SEM), and the captions of Fig 6, Table S5, S9 and S12 explicitly list the SEM's.

Is the variance similar between the groups that are being statistically compared?	Yes this can be seen by the presentations noted throughout the paper as in the response in the previous question.

C- Reagents

6. To show that antibodies were profiled for use in the system under study (assay and species), provide a citation, catalog number and/or clone number, supplementary information or reference to an antibody validation profile. e.g., Antibodypedia (see link list at top right), 1DegreeBio (see link list at top right).	Antibodies used in our studies are all commercially available and the relative information is reported in Supplementary Table S4.
7. Identify the source of cell lines and report if they were recently authenticated (e.g., by STR profiling) and tested for mycoplasma contamination.	All cell lines used are of rodent origin therefore authentication via STR profiling is not possible. The rat origin of the GH3 cell line was confirmed by TaqMan RT-PCR with rat-specific primers/probes for 10 genes. Cell lines were tested routinely for mycoplasma contamination using the MycoAlertTM Mycoplasma Detection Kit from Lonza and always found negative. as reported in Materials and Methods.

* for all hyperlinks, please see the table at the top right of the document

D- Animal Models

8. Report species, strain, gender, age of animals and genetic modification status where applicable. Please detail housing and husbandry conditions and the source of animals.	We used for our studies female CD1 foxn1 nu/nu mice purchased from Charles River, Germany; we use MENX rats which have been previously described in Wiedemann T & Pellegata NS. Mol Cell Endocrinol. 2016 Feb 5;421:49-59. Strains used, age and husbandry conditions are indicated in Materials and Methods.
9. For experiments involving live vertebrates, include a statement of compliance with ethical regulations and identify the committee(s) approving the experiments.	Animal studies were approved by the government of Upper Bavaria, Germany and the protocol numbers are provided in the Materials and Methods section.
10. We recommend consulting the ARRIVE guidelines (see link list at top right) (PLoS Biol. 8(6), e1000412, 2010) to ensure that other relevant aspects of animal studies are adequately reported. See author guidelines, under 'Reporting Guidelines'. See also: NIH (see link list at top right) and MRC (see link list at top right) recommendations. Please confirm compliance.	All the relevant information about our in vivo studies has been included in the article in compliance with the ARRIVE guidelines.

E- Human Subjects

11. Identify the committee(s) approving the study protocol.	The responsible Committees are indicated in the Materials and Methods.
12. Include a statement confirming that informed consent was obtained from all subjects and that the experiments conformed to the principles set out in the WMA Declaration of Helsinki and the Department of Health and Human Services Belmont Report.	This statement is included in the Materials and Methods section.
13. For publication of patient photos, include a statement confirming that consent to publish was obtained.	Not applicable.
14. Report any restrictions on the availability (and/or on the use) of human data or samples.	No restrictions apply.
15. Report the clinical trial registration number (at ClinicalTrials.gov or equivalent), where applicable.	Not applicable.
16. For phase II and III randomized controlled trials, please refer to the CONSORT flow diagram (see link list at top right) and submit the CONSORT checklist (see link list at top right) with your submission. See author guidelines, under 'Reporting Guidelines'. Please confirm you have submitted this list.	Not applicable.
17. For tumor marker prognostic studies, we recommend that you follow the REMARK reporting guidelines (see link list at top right). See author guidelines, under 'Reporting Guidelines'. Please confirm you have followed these guidelines.	Not applicable.

F- Data Accessibility

18: Provide a "Data Availability" section at the end of the Materials & Methods, listing the accession codes for data generated in this study and deposited in a public database (e.g. RNA-Seq data: Gene Expression Omnibus GSE39462, Proteomics data: PRIDE PXD000208 etc.) Please refer to our author guidelines for 'Data Deposition'.<br><br>Data deposition in a public repository is mandatory for:<br>a. Protein, DNA and RNA sequences<br>b. Macromolecular structures<br>c. Crystallographic data for small molecules<br>d. Functional genomics data<br>e. Proteomics and molecular interactions	This study includes no data deposited in external repositories
19. Deposition is strongly recommended for any datasets that are central and integral to the study; please consider the journal's data policy. If no structured public repository exists for a given data type, we encourage the provision of datasets in the manuscript as a Supplementary Document (see author guidelines under 'Expanded View' or in unstructured repositories such as Dryad (see link list at top right) or Figshare (see link list at top right).	Not applicable.
20. Access to human clinical and genomic datasets should be provided with as few restrictions as possible while respecting ethical obligations to the patients and relevant medical and legal issues. If practically possible and compatible with the individual consent agreement used in the study, such data should be deposited in one of the major public access-controlled repositories such as dbGAP (see link list at top right) or EGA (see link list at top right).	Not applicable.
21. Computational models that are central and integral to a study should be shared without restrictions and provided in a machine-readable form. The relevant accession numbers or links should be provided. When possible, standardized format (SBML, CellML) should be used instead of scripts (e.g. MATLAB). Authors are strongly encouraged to follow the MIRIAM guidelines (see link list at top right) and deposit their model in a public database such as Biomodels (see link list at top right) or JWS Online (see link list at top right). If computer source code is provided with the paper, it should be deposited in a public repository or included in supplementary information.	Not applicable.

G- Dual use research of concern

22. Could your study fall under dual use research restrictions? Please check biosecurity documents (see link list at top right) and list of select agents and toxins (APHIS/CDC) (see link list at top right). According to our biosecurity guidelines, provide a statement only if it could.	Not applicable.
