# Supplementary material for: Angpt2/Tie2 autostimulatory loop controls tumorigenesis
Source: EMBO Mol Med. 2022 Mar 10;14(5):e14364. doi: 10.15252/emmm.202114364 (PMC9081903; doi:10.15252/emmm.202114364)
Supplement: Supplementary file 3 — Source Data for Expanded View/Appendix [file EMMM-14-e14364-s005.zip › EMM_2021_14364_Uncropped_App_Figs.zip!/EMM_2021_14364_Uncropped_App_Fig_S11.pdf]

Full unedited gel for Appendix Figure S11A:

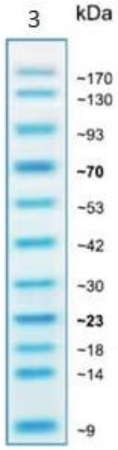

Prestained marker used for Appendix Fig. S11A and B

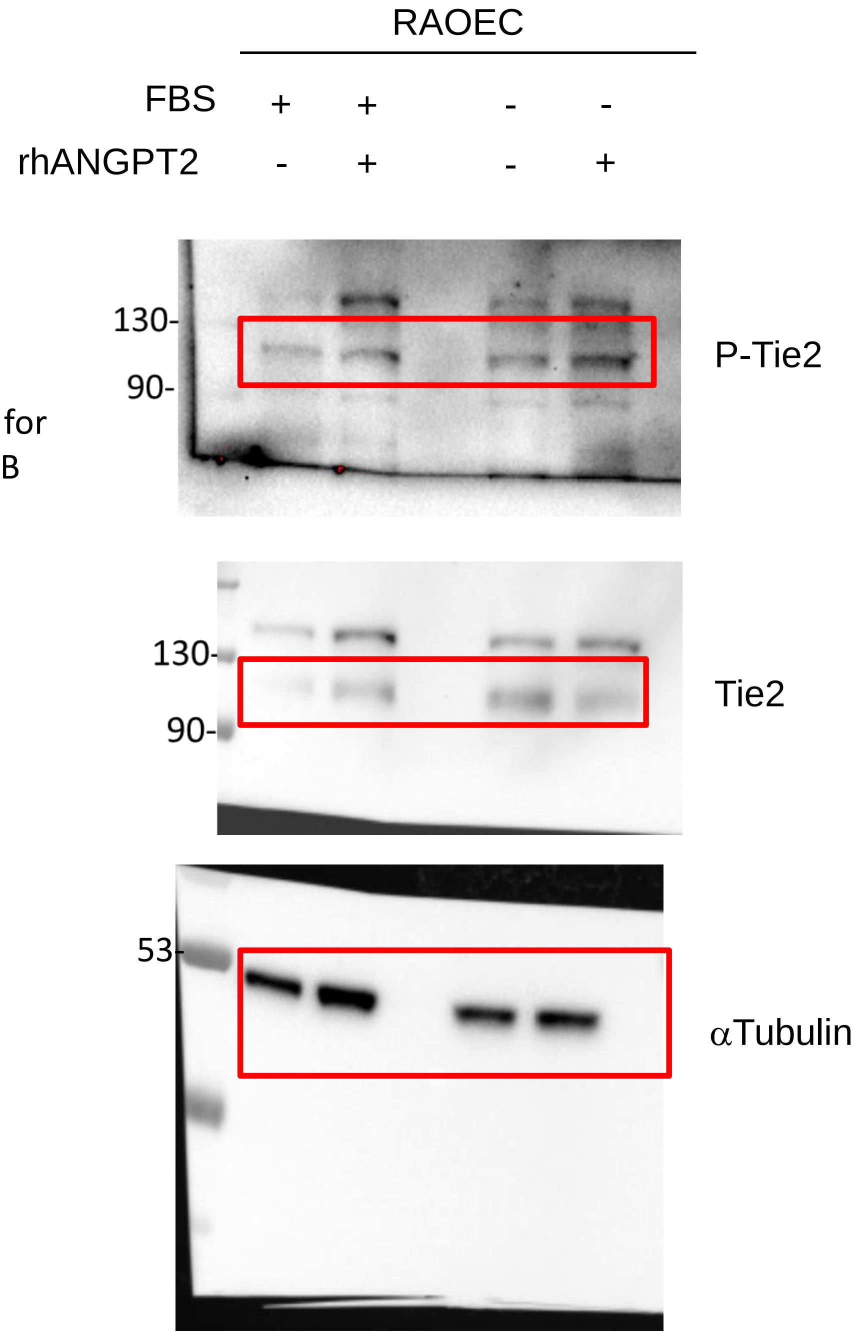

The membrane was cut at 70kDa and the upper half was probed for pTie2, the lower for Tubulin. The upper membrane was then stripped and re-probed for Tie2

Full unedited gel for Appendix Figure S11B:

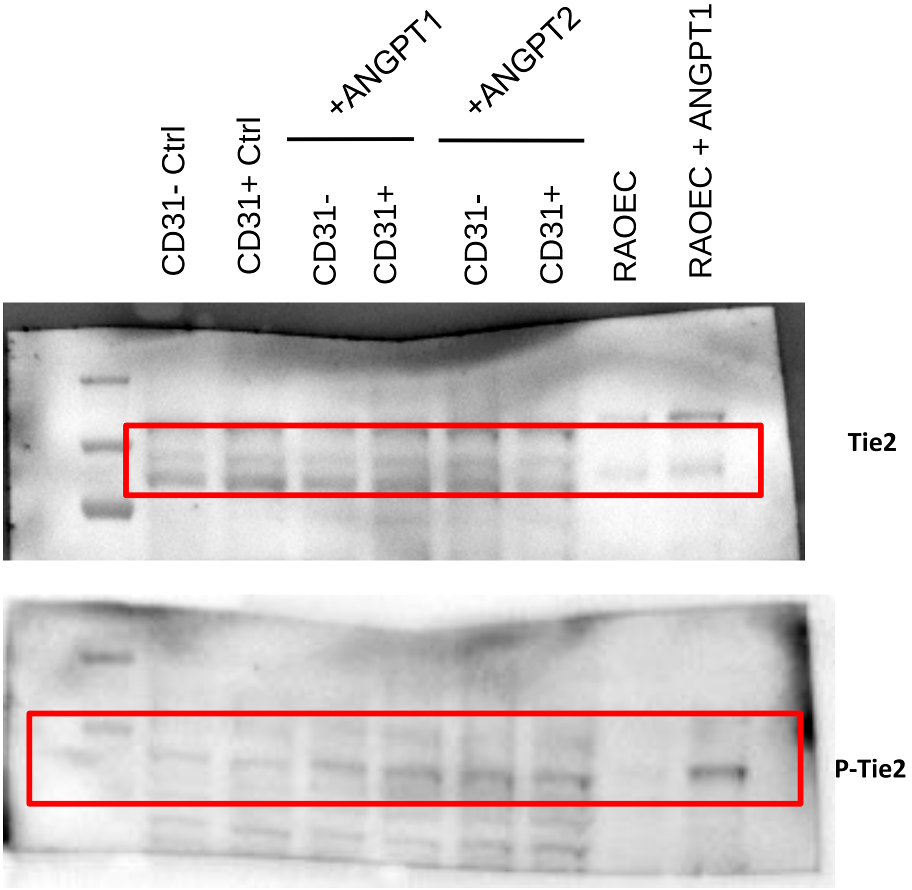

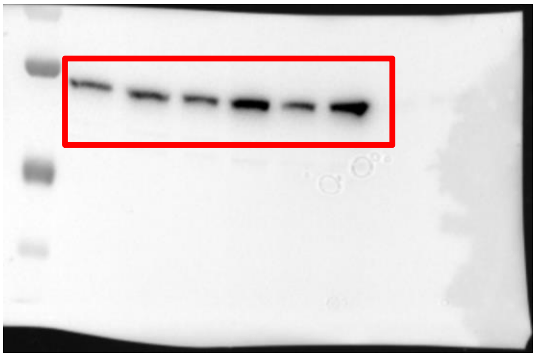

Alpha Tubulin (short exposure)

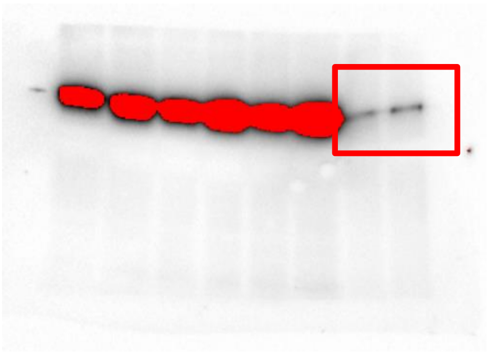

Alpha Tubulin (long exposure)
