# Supplementary material for: Angpt2/Tie2 autostimulatory loop controls tumorigenesis
Source: EMBO Mol Med. 2022 Mar 10;14(5):e14364. doi: 10.15252/emmm.202114364 (PMC9081903; doi:10.15252/emmm.202114364)

# Full unedited gel for Appendix Figure S13A:

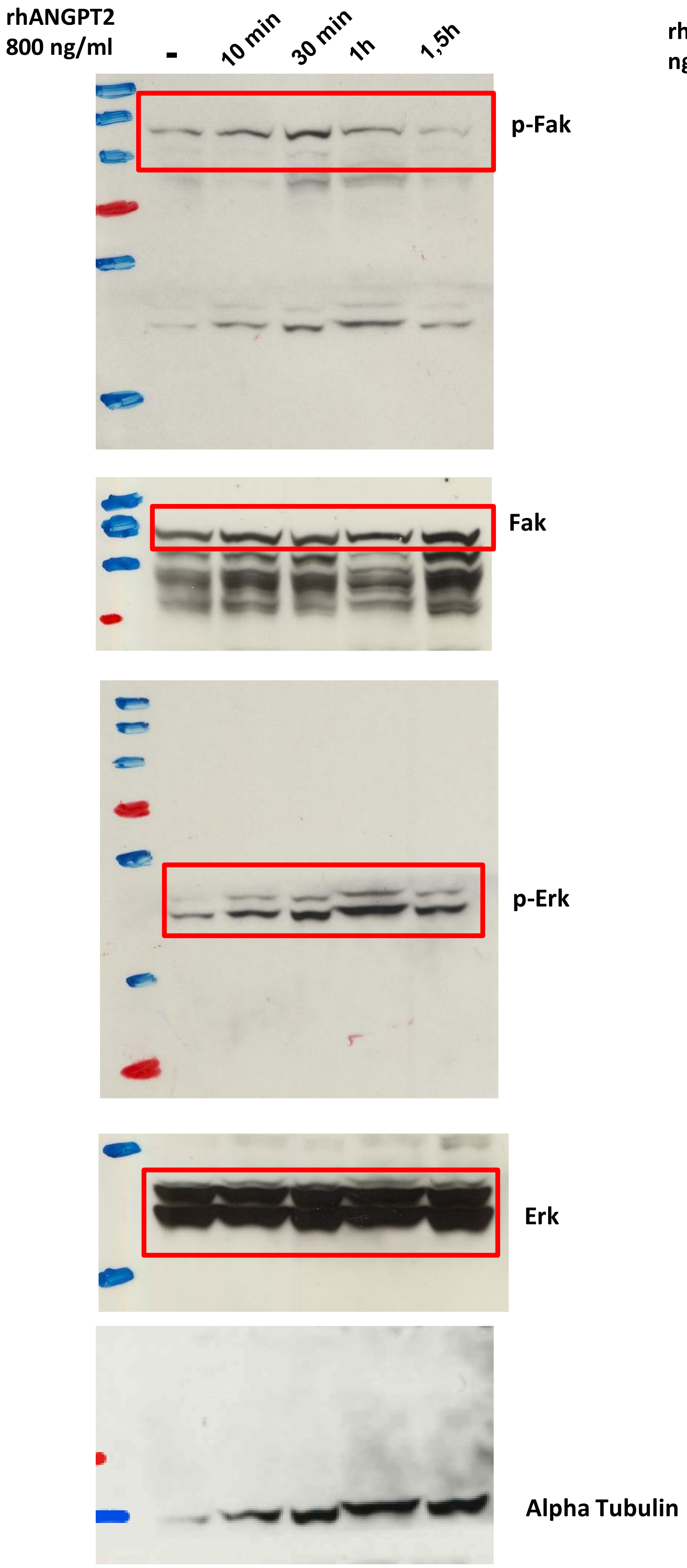


The membrane was stripped and cut and each half was treated with the corresponding antibodies

# Full unedited gel for Appendix Figure S13B:

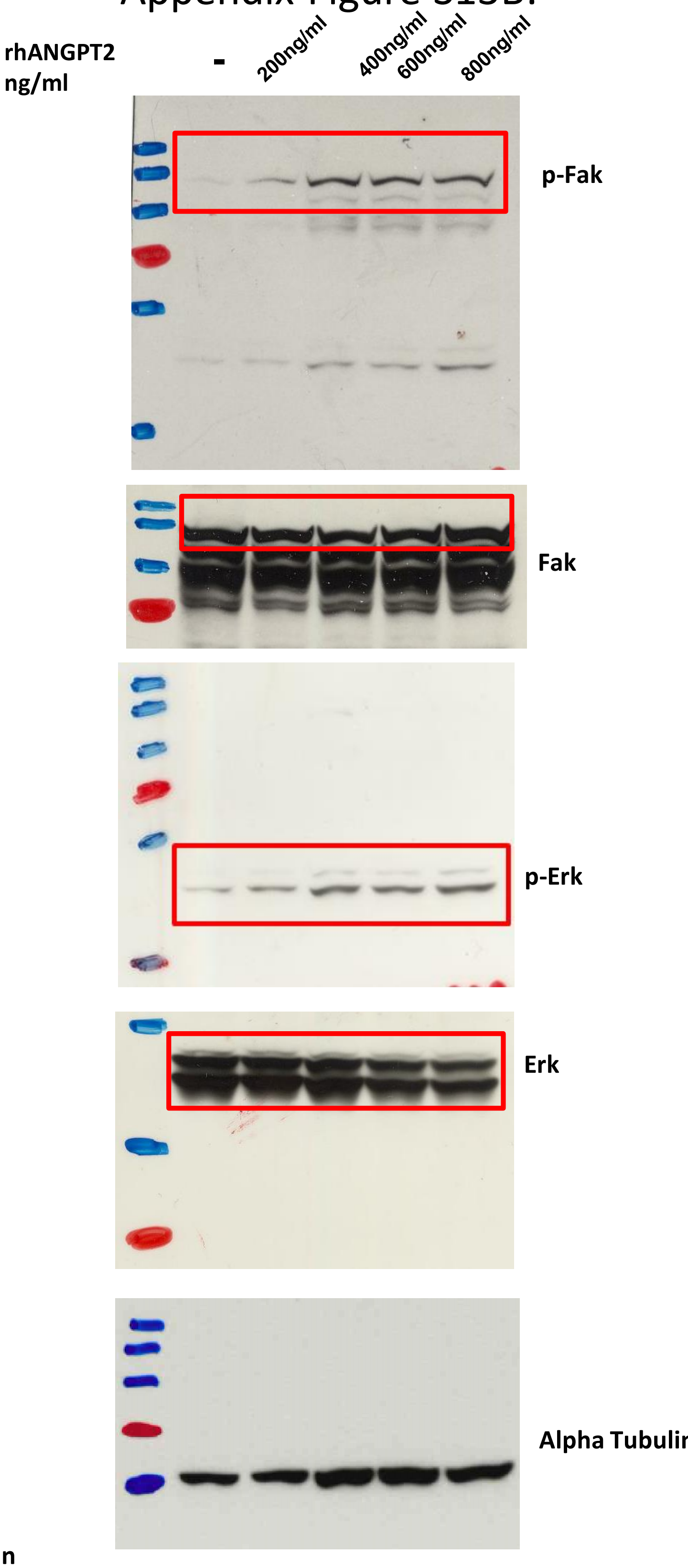


The membrane was stripped and cut and each half was treated with the corresponding antibodies

# Full unedited gel for Appendix Figure S13C:

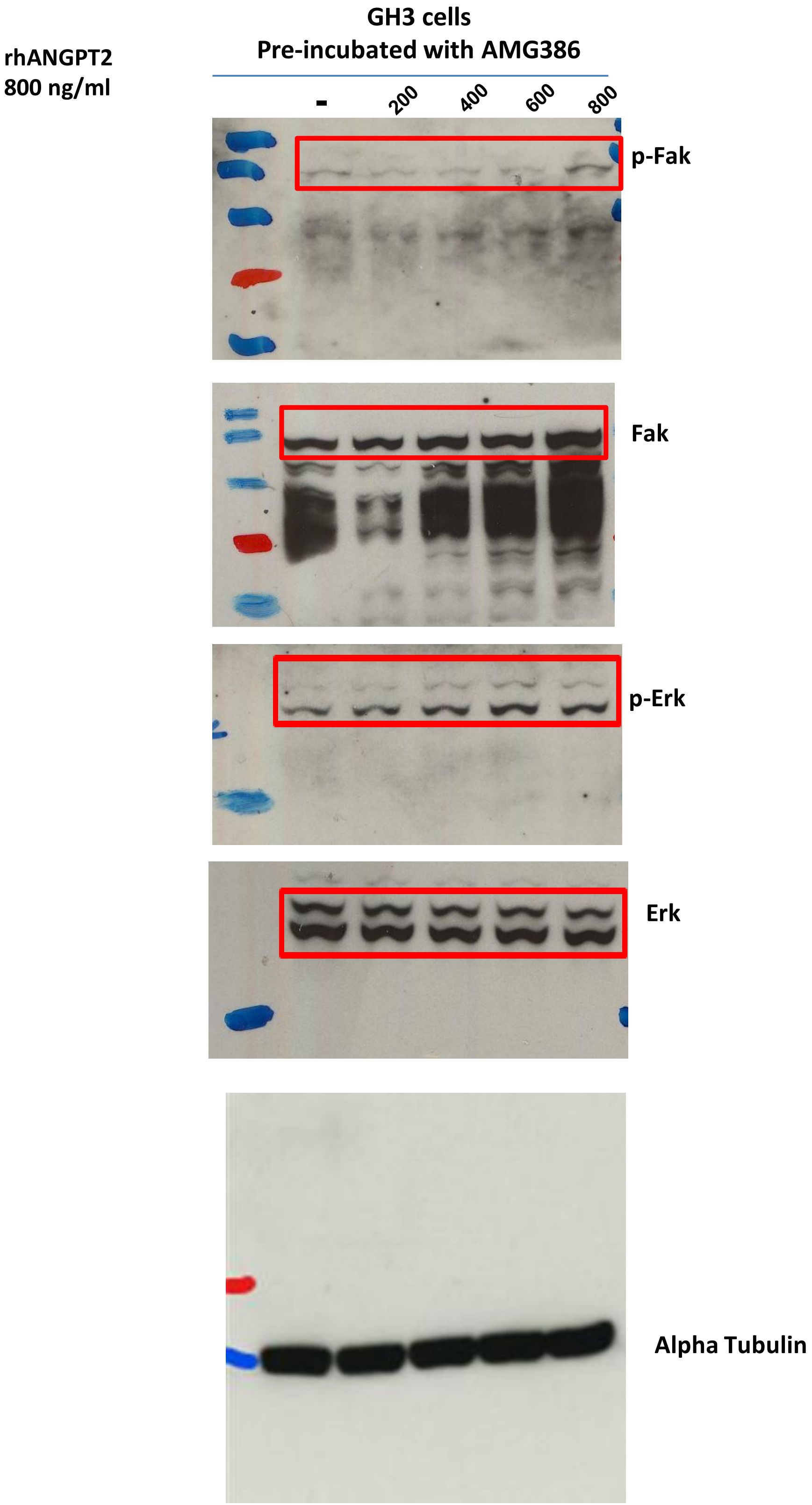

Supplement: Supplementary file 3 — Source Data for Expanded View/Appendix [file EMMM-14-e14364-s005.zip › EMM_2021_14364_Uncropped_App_Figs.zip!/EMM_2021_14364_Uncropped_App_Fig_S13.pdf]
