# Supplementary material for: Angpt2/Tie2 autostimulatory loop controls tumorigenesis
Source: EMBO Mol Med. 2022 Mar 10;14(5):e14364. doi: 10.15252/emmm.202114364 (PMC9081903; doi:10.15252/emmm.202114364)

Full unedited gel for Figure EV4E:

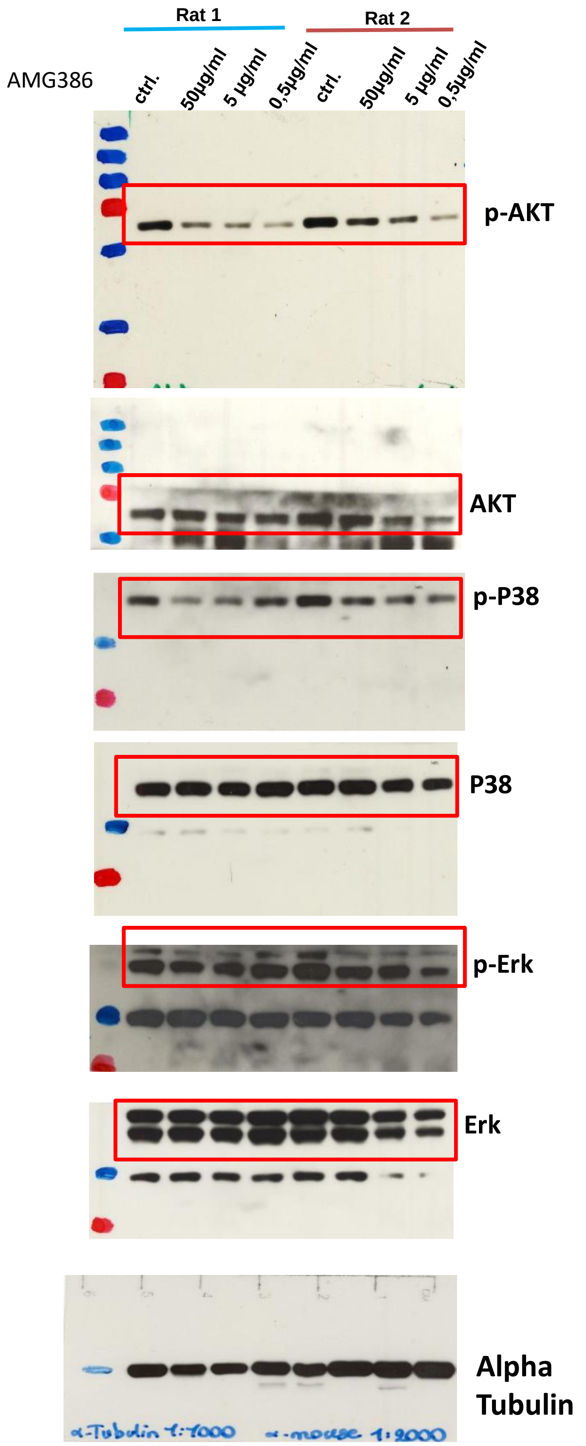

After treatment with p-Akt, the membrane was stripped and cut in the middle to avoid multiple stripping steps and each half was treated with the corresponding antibodies

Full unedited gel for Figure EV4F:

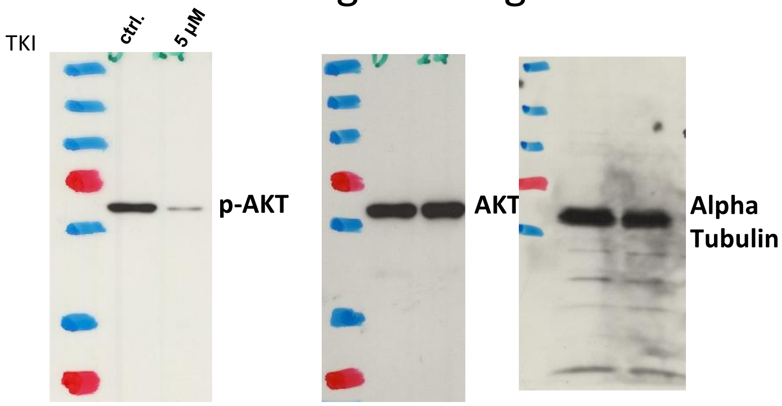

Full unedited gel for Figure EV4K:

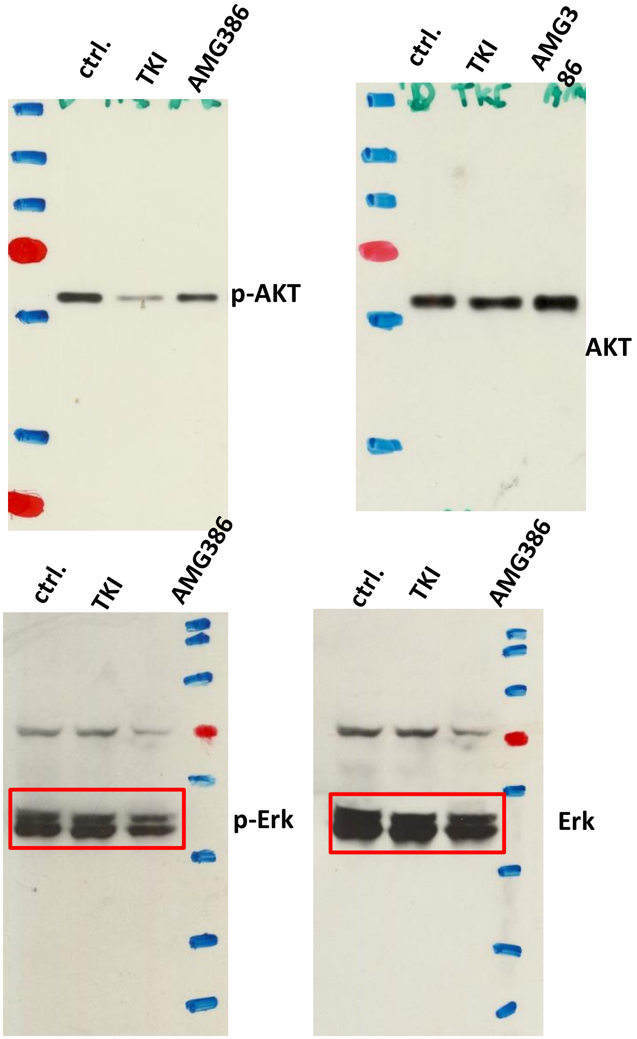

Proteins were loaded again to detect pErk and Erk

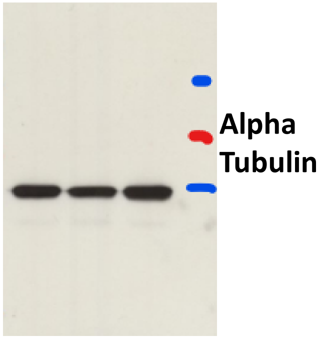

Supplement: Supplementary file 3 — Source Data for Expanded View/Appendix [file EMMM-14-e14364-s005.zip › EMM_2021_14364_Uncropped_EV_Figures_.zip!/EMM_2021_14364_Uncropped_Fig_EV4E_F_K.pdf]
