# Supplementary material for: Angpt2/Tie2 autostimulatory loop controls tumorigenesis
Source: EMBO Mol Med. 2022 Mar 10;14(5):e14364. doi: 10.15252/emmm.202114364 (PMC9081903; doi:10.15252/emmm.202114364)

EV figures

Full unedited gel for Figure EV2A:

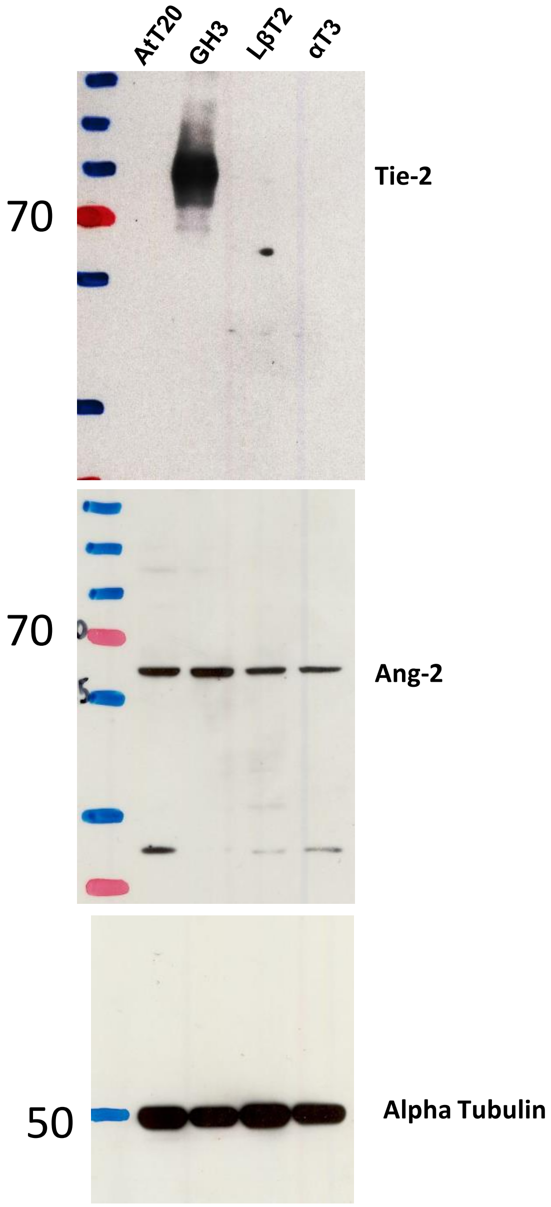

Supplement: Supplementary file 3 — Source Data for Expanded View/Appendix [file EMMM-14-e14364-s005.zip › EMM_2021_14364_Uncropped_EV_Figures_.zip!/EMM_2021_14364_Uncropped_Fig_EV2.pdf]
